# Longitudinal relationships across sleep, physical activity, and mental wellbeing in early-to-mid-adolescence: a developmental cascades investigation

**DOI:** 10.1007/s11136-025-03894-2

**Published:** 2025-01-28

**Authors:** Jose Marquez, Margarita Panayiotou, Reihaneh Farzinnia, Qiqi Cheng, Neil Humphrey

**Affiliations:** Manchester Institute of Education, University of Manchester, Manchester, M13 9PL UK

**Keywords:** Mental health, Wellbeing, Adolescence, Sleep, Physical activity, Developmental cascades

## Abstract

**Purpose:**

Sleep (SL), physical activity (PA), and wellbeing (WB) are three factors linked to positive development in adolescence. Despite theoretical support and some empirical evidence of developmental associations between these factors, few studies have rigorously investigated reciprocal associations over time separating between-person and within-person effects, and none have investigated all three in concert. Thus, it remains unclear how the interplay between SL, PA and WB unfolds across time within individuals. This study examines this question in the crucial early-to-mid-adolescence developmental transition.

**Method:**

Separating between- and within-person effects, a random-intercept cross-lagged panel model was fitted to a dataset of *N* = 27,949 adolescents (age 12/13 at first timepoint) from Greater Manchester, England, using a three-by-three design (three annual timepoints: T1, T2, T3; three variables: SL, PA, WB).

**Results:**

Analyses revealed gender-specific developmental cascade pathways. Specifically, we found positive reciprocal associations between SL and WB for girls (at T1→T2), whereas for boys, SL positively predicted WB (at both T1→T2 and T2→Τ3) but WB did not predict SL. We also found that WB predicted PA for boys (at T2→T3) but this finding was sensitive to model specification and yielded a smaller effect than other cross-lagged pathways.

**Conclusion:**

Our results highlight the importance of sleep as a driver of adolescent wellbeing, and the role of gender in developmental cascade processes. Study strengths, limitations, and implications are discussed.

**Supplementary Information:**

The online version contains supplementary material available at 10.1007/s11136-025-03894-2.

## Introduction

Developmental cascades theory [1] proposes that functioning within and across domains, levels, and systems is developmentally intertwined, and that specific functions and behaviours ‘spread’ over time into other functions and behaviours through complex chain reactions. The current study focuses on developmental cascades involving adolescent sleep, physical activity, and mental wellbeing, each of which is known to deteriorate throughout adolescence, particularly so among girls [2–5], and each of which is concurrently and prospectively associated with better health, social, and economic outcomes [6–8].

Multiple neurobiological, psychosocial and behavioural processes have been proposed to explain how and why sleep, physical activity and wellbeing may be reciprocally related across time within individuals [9, 10]. For example, physical activity may increase wellbeing via its impact on the functioning of the hypothalamus-pituitary-adrenal axis, which in turn reduces cortisol levels [10], while it may also improve sleep via its effects on the circadian system [11]. Similarly, poor sleep may be associated with more frequent use of maladaptive emotion regulation strategies, which in turn negatively impact wellbeing [12]. Despite these insights, no prior longitudinal investigation has comprehensively examined the interconnectedness of sleep, physical activity, and wellbeing in concert. Such a holistic examination is critical given the increasing acknowledgement of the principles of cascades theory in developmental science [13] and holds the potential to offer invaluable insights for devising effective interventions that interrupt negative cascades and foster positive reciprocal effects, leading to favourable outcomes [1].

**Fig. 1:**
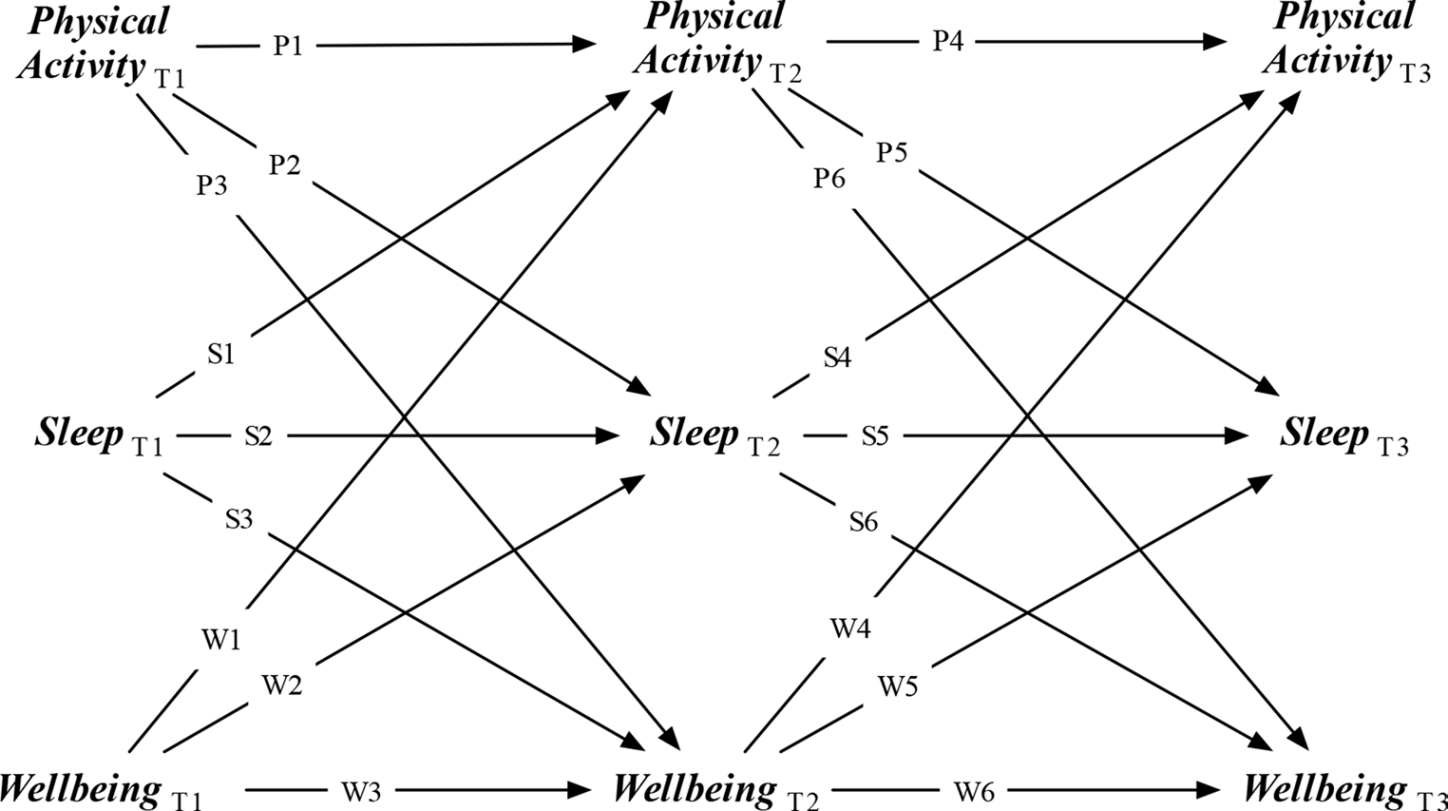
Conceptual diagram

Building on these theoretical perspectives, the current study uses a Random-Intercept Cross-Lagged Panel Model (RI-CLPM) to investigate reciprocal longitudinal relationships between sleep quality, physical activity, and mental wellbeing in early-to-mid-adolescence (see Fig. 1 for conceptual model). RI-CLPM are considered superior to traditional CLPM and group-based regression as they distinguish between-person variance (i.e., stable differences between individuals) and within-person variance (i.e., situational changes within individuals) [14–17]. This distinction is crucial for accurately delineating cascading effects within individuals and preventing estimates confounded by between-person relationships (which can lead to flawed conclusions about the presence, prevalence, and direction of within-person causal influences) [14, 16, 18]. It also has substantial practical implications: within-person associations prompt suitable targets for intervention, while between-person associations help to determine who needs intervention [17, 19]. Existing research has yet to provide robust evidence of within-person cascade effects between sleep quality, physical activity, and mental wellbeing in early-to-mid-adolescence. By addressing this research gap, the present study offers a significant contribution to our understanding of the interplay between health behaviours and mental health, which can in turn prompt evidence-informed policy and practice responses that can foster positive outcomes during a critical developmental phase.

### The relationships between sleep, physical activity, and wellbeing in adolescence: what do we know?

In the subsections that follow, we review existing evidence on the relationships between our three domains of interest in adolescence. Given the sparsity of research focusing specifically on wellbeing (i.e., feeling good and functioning well) [20], the review also draws on evidence pertaining to demonstrably related constructs, including mental health difficulties (e.g., depression) and health-related quality of life [21]. However, while such constructs are related to wellbeing, we acknowledge that they should not be treated as completely synonymous (i.e., the jingle-jangle fallacy) [22]. Accordingly, for clarity, we note the specific construct measured in our reference to existing studies.

### Physical activity (PA) and wellbeing (WB)

Systematic reviews and meta-analyses have consistently found small positive cross-sectional associations between physical activity and mental health [23], and psychological health indicators in children and young people [24]. In Granger et al.’s [25] systematic review, stronger effects for boys than for girls were observed in two studies [26, 27]. In another systematic review of physical activity interventions in schools, no gender differences in the improved outcomes (such as positive wellbeing, mental health, increased resilience, and reduced anxiety) were found [28].

Where longitudinal evidence is available, a meta-analysis and a systematic review found small temporal associations between physical activity and fewer depressive symptoms (PA→WB; P3 and P6 Fig. 1) [29, 30]. Studies exploring effects in the opposite direction (WB→PA; W1 and W4 in Fig. 1) are rare, but there is evidence that young adults who reported high or escalating levels of depression symptoms during adolescence tend to engage in less moderate physical activity and are also less inclined to participate in team sports [31].

Through our search, we found two studies that explored reciprocal longitudinal associations (PA↔WB) in adolescence using regression analysis. There is evidence of reciprocal longitudinal associations between physical activity and health-related quality of life (HRQoL) (i.e., an individual’s satisfaction in various life domains affected by health) [32]. Reciprocal longitudinal links between exercise and depression were also found in a sample of female adolescents [33]. Using latent growth modelling to analyse a longitudinal sample covering the ages from 10 to 21 years, Gunnell et al. [34] found higher initial symptoms of depression predicted greater decreases in physical activity (WB→PA), but not the other way around.

Using CLPM, Jensen et al. [35] found higher initial levels of HRQoL to predict future physical activity engagement in preadolescent children, but not the other way around. Only three studies to date have used RI-CLPM. Groß et al.’s [36] study of 8–14-year-olds found a medium-to-strong reciprocal relationship between HRQoL and physical activity (PA↔WB), as well as a longitudinal unidirectional association between higher emotional symptoms and later sport participation (WB→PA). Similarly, Graupensperger et al. [37] observed reciprocal effects between emotional symptoms and team sports involvement (WB↔PA). Finally, focusing on the transition from adolescence to early adulthood, Ames et al. [38] found that within-person increases in physical activity did not predict changes in depressive symptoms after two years (PA→WB), but that within-person increases in depressive symptoms did predict reductions in physical activity (WB→PA).

### Sleep (SL) and wellbeing (WB)

In a systematic review, Ong et al. [39] reported a connection between positive affect and improved sleep in population studies (including but not limited to adolescence), but noted that the majority were classified as weak or having high risk of bias. The authors noted that the majority of longitudinal studies have primarily focused on unidirectional effects, where (for example) sleep difficulties in adolescence predict later psychiatric difficulties (SL→WB, S3 and S5 in Fig. 1) [40] and sleep duration predicts subjective well-being [41]. Findings from two studies suggest that gender may moderate the SL→WB association. For example, Nunes et al. [42] found that high persistent difficulties sleeping predicted later internalizing problems in adolescent females, but not males. In another adolescent study, gender and depressive symptoms did moderate the effects of sleep on affect but not the other way around [43].

Reciprocal longitudinal associations between sleep disturbances or duration, and anxiety and/or depression in adolescents (SL↔WB, S3, S5, W2, and W5 in Fig. 1) have been found in four longitudinal studies [44–47]. However, in their systematic review of the evidence base, Alvaro et al. [48] noted that drawing firm conclusions about these relationships is challenging due to methodological limitations and the small number and heterogeneity of samples.

More recently, using CLPM, Kelly & El-Sheikh [49] found reciprocal associations between children’s sleep and internalising and externalising symptoms over time (SL↔WB), although effects were stronger for SL→WB pathways. Using RI-CLPM, Narmandakh et al., [50] found no evidence of anxiety symptoms predicting the onset of sleep problems (WB→SL), though they did report that insufficient sleep during early and middle adolescence may contribute to later increases in anxiety (SL→WB).

### Physical activity (PA) and sleep (SL)

Compared to the above, the links between physical activity and sleep have received less empirical scrutiny, and existing systematic reviews and meta-analyses report mixed findings among children and adolescents. For example, Lang et al. [51] found medium to large associations, while Antczack et al. [52] found little evidence of an overall association, with some weak positive associations between vigorous physical activity and sleep derived almost exclusively from cross-sectional evidence. To our knowledge, only two studies to date have assessed reciprocal longitudinal effects (PA↔SL, P2, P5, S1, and S4 in Fig. 1). Using CLPM to analyse a sample of university students, Semplonius and Willoughby [53] found support for such a relationship (at least for moderate physical activity), but only indirectly through emotion regulation. Using RI-CLPM, Pesonen et al. [54] identified a small reciprocal association between sleep duration and vigorous physical activity in adults.

### The current study

There is theoretical support for potential reciprocal longitudinal associations between sleep, physical activity and wellbeing in adolescence. Research to date has provided some evidence of these associations, including potential gender differences. However, the current evidence base is limited. First, studies investigating *reciprocal* longitudinal associations are scarce, particularly so when considering gender moderation effects. Second, most existing studies have used approaches such as group-based regression or CLPM, which do not allow separation of between-person and within-person effects. Third, among the few studies using RI-CLPM to investigate these associations, none have considered sleep, physical activity and wellbeing in concert (i.e., they have only considered two of the three constructs). Fourth, studies to date have almost exclusively focused on related constructs (e.g. depression) rather than mental wellbeing specifically. The latter arguably has greater utility in population mental health research, given that relatively few adolescents in non-clinical samples would meet diagnostic criteria for disorder, and dimensional symptom measures typically show substantial floor effects [21, 55, 56].

As a result, how the interplay between sleep, physical activity and wellbeing unfolds across time within individuals during the critical transition from early to middle adolescence– and how this potentially varies by gender - remains unclear. Using RI-CLPM to distinguish within-person from between-person effects, our study addresses this important developmental cascade research problem. In our analysis, we control socio-demographic characteristics (gender, free school meal eligibility (FSM), special educational needs (SEN), and ethnicity) known to explain variance in sleep, physical activity, and wellbeing [57–59]. Moreover, we examine gender differences given the evidence presented in the literature review indicating that this (but not the other socio-demographic variables we use as covariates) may be an important moderator of the associations between our constructs of interest. Table 1 summarises our study hypotheses, possible outcomes, and interpretation.

**Table 1:** Study hypotheses, possible outcomes, and interpretation

Hypothesis and underpinning theory/model		Prediction(s) (path directions in Fig. 1 where applicable)	Possible outcomes (*p* <.05)	Interpretation
H1: Physical activity has positive consequences for later mental wellbeing and sleep quality	H1A: Higher weekly estimates of physical activity predicts, a year later, more positive reports of mental wellbeing Theoretical support: Developmental cascades model [1] Empirical support: [17–19,21]	P3 > 0, P6 > 0	P3 > 0, P6 > 0 P3 > 0, P6 ≤ 0 (or, P3 ≤ 0, P6 > 0) P3 ≤ 0, P6 ≤ 0	H1A supported H1A partially supported H1A rejected
	H1B: Higher weekly estimates of physical activity predicts, a year later, that they are also more likely to think they are getting enough sleep Theoretical support: Developmental cascades model [1]	P2 > 0, P5 > 0	P2 > 0, P5 > 0 P2 > 0, P5 ≤ 0 (or, P2 ≤ 0, P5 > 0) P2 ≤ 0, P5 ≤ 0	H1B supported H1B partially supported H1B rejected
H2: Sleep quality has positive consequences for later physical activity and mental wellbeing	H2A: Thinking that they are getting enough sleep predicts, a year later, higher weekly estimates of physical activity Theoretical support: Developmental cascades model [1] Empirical support: [45]	S1 > 0, S4 > 0	S1 > 0, S4 > 0 S1 > 0, S4 ≤ 0 (or, S1 ≤ 0, S4 > 0) S1 ≤ 0, S4 ≤ 0	H2A supported H2A partially supported H2A rejected
	H2B: Thinking that they are getting enough sleep predicts, a year later, more positive reports of their mental wellbeing Theoretical support: Developmental cascades model [1] Empirical support: [33,34,43]	S3 > 0, S6 > 0	S3 > 0, S6 > 0 S3 > 0, S6 ≤ 0 (or, S3 ≤ 0, S6 > 0) F3 ≤ 0, F6 ≤ 0	H2B supported H2B partially supported H2B rejected
H3: Mental wellbeing has positive consequences for later physical activity and sleep quality	H3A: More positive reports of their mental wellbeing predicts, a year later, higher weekly estimates of physical activity Theoretical support: Developmental cascades model [1] Empirical support: [24,27,28,31]	W1 > 0, W4 > 0	W1 > 0, W4 > 0 W1 > 0, W4 ≤ 0 (or W1 ≤ 0, W4 > 0) W1 ≤ 0, W4 ≤ 0	H3A supported H3A partially supported H3A rejected
	H3B: More positive reports of their mental wellbeing predicts, a year later, that they are also more likely to think that they are getting enough sleep Mental wellbeing positively predicts later sleep quality Theoretical support: Developmental cascades model [1]	W2 > 0, W5 > 0	W2 > 0, W5 > 0 W2 > 0, W5 ≤ 0 (or, W2 ≤ 0, W5 > 0) W2 ≤ 0, W5 ≤ 0	H3B supported H3B partially supported H3B rejected
H4: Physical activity, sleep and mental wellbeing are stable within-persons over time	H4A: Higher weekly estimates of physical activity predicts, a year later, higher weekly estimates of physical activity Theoretical support: Developmental cascades model [1]	P1 > 0, PB4 > 0	P1 > 0, P4 > 0 P1 > 0, P4 ≤ 0 (or P1 ≤ 0, P4 > 0) P1 ≤ 0, P4 ≤ 0	H4A supported H4A partially supported H4A rejected
	H4B: Thinking that they are getting enough sleep predicts, a year later, thinking that they are getting enough sleep. Theoretical support: Developmental cascades model [1]	S2 > 0, S5 > 0	S2 > 0, S5 > 0 S2 > 0, S5 ≤ 0 (or S21 ≤ 0, S5 > 0) S2 ≤ 0, S5 ≤ 0	H4B supported H4B partially supported H4B rejected
	H4C: More positive reports of their mental wellbeing predicts, a year later, more positive reports of their mental wellbeing. Theoretical support: Developmental cascades model [1]	W3 > 0, W6 > 0	W3 > 0, W6 > 0 W3 > 0, W6 ≤ 0 (or W3 ≤ 0, W6 > 0) W3 ≤ 0, W6 ≤ 0	H4C supported H4C partially supported H4C rejected
H5: Physical activity, sleep quality, and mental wellbeing share a reciprocal relationship over time Theoretical support: Developmental cascades model [1] Empirical support: SL↔WB: [37–40] WB↔PA: [25,26,31,30] PA↔SL: [44,47]			H1, H2, *and* H3, supported or partially supported Any other outcome	H5 supported H5 rejected
H6: Developmental cascades between physical activity, sleep, and mental wellbeing vary by gender Empirical support: [2–5,36]		Multi-group RI-CLPM analyses (chi-square difference tests and the test of small difference in fit) will indicate structural variance by gender	Multi-group RI-CLPM analyses indicate structural variance by gender Multi-group RI-CLPM analyses indicate structural invariance by gender	H6 supported, RI-CLPM conducted separately for girls and boys H6 rejected, single RI-CLPM conducted

## Method

### Design

Our analysis uses the three annual data points of the longitudinal cohort in the #BeeWell study in Greater Manchester, England [60], using a three-by-three panel design (three annual data points: T1, autumn 2021; T2, autumn 2022; and T3, autumn 2023, for our three focal variables: sleep quality, SL; physical activity, PA; and, mental wellbeing, WB). Ethical approval for #BeeWell was granted by the University of Manchester ethics committee (Ref: 2021-11133-18965).

### Participants

To optimise sample size, we used a ‘drop in’ design [19] in which any participant in the longitudinal #BeeWell cohort with data for at least one time point was included in the analysis (Model A; *N* = 27,949). As a pre-planned sensitivity analysis, an additional model was estimated excluding those respondents with data at only one time point (Model B, *N* = 17,552; see Appendix 5).

The main analytical sample (Model A) noted above is comprised of the following: (i) At T1 (age 12–13), a total of *N* = 20,130 completed the #BeeWell survey; (ii) at T2 (age 13–14), *N* = 17,700 completed it, of whom *N* = 12,100 also participated at T1, and *N* = 5,600 joined at T2; (iii) at T3 (age 14–15), a total of *N* = 17,005 completed the #BeeWell survey, of whom *N* = 11,556 also participated at T1, and *N* = 11,513 also participated at T2; and, (iv) a total of *N* = 8,298 students participated in all the three waves. The main analytical sample are drawn from k = 176 schools that participated in at least one time point.

The socio-demographic characteristics of the sample in Model A are as follows: 13,982 (50.03%) boys, and 13,967 (49.97%) girls; 9,649 (35.26%) ethnic minority, and 17,718 (64.74%) white; 7,678 (28.32%) eligible for Free School Meals (FSM), and 19,433 (71.68%) not eligible for FSM; 4,504 (16.51%) identified as having special educational needs or disabilities (SEND), and 22,773 (83.49%) not identified as having SEND. For reference, Greater Manchester population data for students aged 12–15 between 2021/2022 and 2023/2024 [61, 62] shows 51.23% boys, 37.60% ethnic minority, 29.48% eligible for FSM, and 19.03% identified as having SEND.

### Measures

#### Mental wellbeing

The Short Warwick Edinburgh Mental Wellbeing Scale (SWEMWBS), derived from seven items (I’ve been feeling optimistic about the future; I’ve been feeling useful; I’ve been feeling relaxed; I’ve been dealing with problems well; I’ve been thinking clearly; I’ve been feeling close to other people; I’ve been able to make up my own mind about things) that use a five-point response format (None of the time; Rarely; Some of the time; Often; All of the time) [63], draws on both subjective and eudaimonic wellbeing perspectives and has favourable psychometric properties [64]. This was modelled as a latent variable to account for measurement error. Internal consistency was good (T1: α = 0.85, T2: α = 0.87, T3: α = 0.89) and confirmatory factor analysis indicated good fit to the data (CFI = 0.983, TLI = 0.981, RMSEA = 0.035, SRMR = 0.028).

#### Sleep quality

A binary (yes, no) item derived from the Health Behaviours in School-Aged Children (HBSC) [65] survey asked respondents if the amount of sleep they normally get is sufficient for them to feel awake and concentrate on their schoolwork during the day.

#### Physical activity

Following a definition of physical activity, two items derived from HBSC [65] asked respondents (i) how many days in a usual week they are physically active; and, (ii) on those days, how long on average they spend being physically active (Around 0.5 h; Around 1 h; Around 1.5 h; Around 2 h or more). The resultant data were combined to produce a quasi-continuous estimate of weekly physical activity.

#### Covariates

Gender (boys, girls)^1^, ethnicity (White, UK minority ethnic group), FSM (eligible, not eligible), and SEND (identified as having SEND, no SEND) were used as covariates.

### Data analyses

Analyses were conducted using Mplus version 8.6. The syntax used for the analysis is presented in the supplementary materials (Appendix 1).

Prior to the estimation of the RI-CLPM, we reviewed the data to examine multicollinearity (see Table 2; mean VIF = 1.50; Highest VIF (PAT2) = 1.74), missingness and normality (see supplementary materials, Appendix 2), covariate effects (see Appendix 3), and measurement invariance (see Appendix 4). Upon confirming in our analysis of missingness that it was required (see Appendix 2), we employed multiple imputation using 100 imputed data sets, as planned in Stage 1 submission^2^ and in line with guidance by Graham et al. [66]. Any variables found to predict missingness (see Appendix 2) were utilised as auxiliary variables.

We used the robust weighted least squares (WLSMV) estimator due to the use of a binary variable (sleep quality). Satisfactory model fit was indicated by Tucker–Lewis index (TLI) and comparative fit index (CFI) values above 0.95 (0.99 for both), root mean square error of approximation (RMSEA) values below 0.06 (0.02), and standardized root mean squared residual (SRMR) values below 0.08 (0.02) [67]. The goodness-fit-statistics and the standard errors of the parameters were adjusted (using type = complex) to account for the clustered nature of the data.

**Fig. 2:**
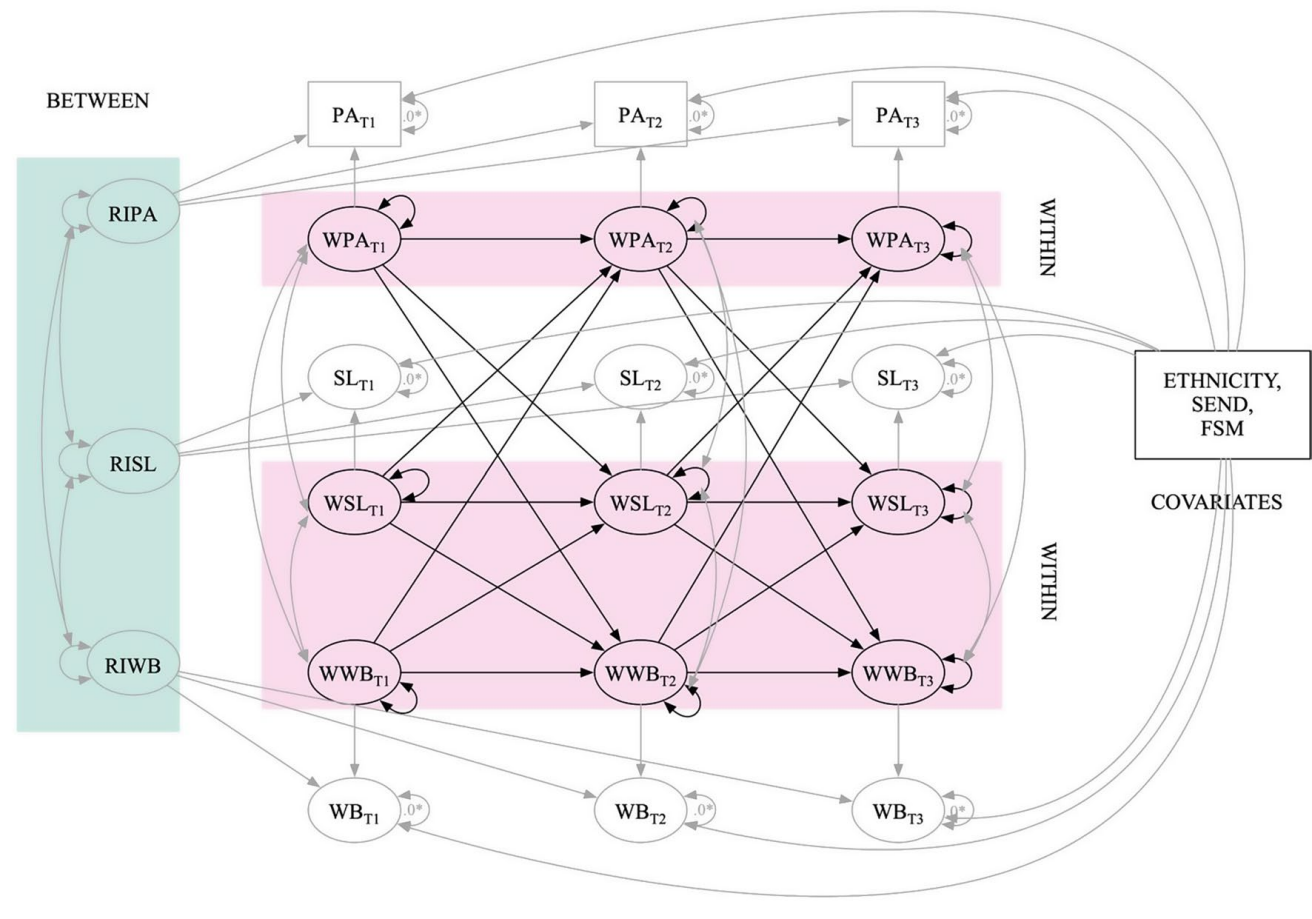
Statistical model for main RI-CLPM developmental cascades analysis. Note: PA, SL and WB represent physical activity, sleep and mental wellbeing respectively. RI and W before PA, SL and WB represent random intercepts and within components of physical activity, sleep and mental wellbeing, respectively

Following Hamaker et al.‘s [16] recommended procedure, RI-CLPM was used to examine autoregressive and reciprocal associations between physical activity, sleep quality and mental wellbeing over three waves of measurement while controlling for time-invariant covariates as described in Fig. 2. Mental wellbeing was modelled as a latent variable to account for measurement error. This allows for the estimation of autocorrelations among residual variances and the constraining of factor loadings and thresholds to equality–assuming longitudinal measurement invariance is established (see Appendix 4). These analytical techniques provide more accurate estimates of the cross-lagged paths [68].

As planned in the Stage 1 submission, we assessed structural variance by gender by comparing a multiple group RI-CLPM with no constraints across the two groups (boys and girls) with another model in which the regression coefficients were constrained to be the same for boys and girls [69] using the Wald chi-square test. Upon finding evidence of structural variance by gender (see Appendix 4), we conducted RI-CLPM separately for boys and girls, and gender was therefore not used as a covariate. To assess gender differences in specific autoregressive and cross-lagged paths, we could not use chi-square difference test as this is not possible when using imputed data in MPlus. Therefore, we used Wald chi-square test to compare, for each path of interest, the unconstrained model with the model where the regression coefficient of the path was constrained to be the same for girls and boys. As noted earlier, we estimated two models. Our main analytical Model A included respondents who took the survey at least once, and our sensitivity analysis Model B (reported in Appendix 5) included those who took it in at least two of the three waves.

### Interpretation of between and within person effects

When analysing distinct pathways and associations among variables in the RI-CLPM (noted in Fig. 1), we originally (at Stage 1) considered an alpha level below 0.01 as indicative of statistical significance when drawing conclusions regarding each of the hypotheses outlined in Table 1. This initial choice aimed to address the issue of multiple comparisons and our substantial sample size, both of which impact p-values [70, 71]. However, we ultimately decided to set al.pha levels below 0.05 as indicative of statistical significance, for two reasons. First, it has been argued that, in the context of conjunctive hypothesis testing in which all relevant results must be significant in order to fully reject the null hypothesis (as is the case in the current study), alpha adjustment is not appropriate [72]. Second, RI-CLPM produces standard errors that are 1.3 to 2.6 times larger than CLPM, leading to a lower power to detect a substantial and relevant effect. In part, this is because models that separate between- and within-person effects reduce bias, but at the cost of efficiency [73–75]. Nevertheless, in the interests of transparency, we report exact p-values for cross-lagged and autoregressive effects in the tables, and whether they are < 0.05, <0.01, or < 0.001 in the main text. In the discussion section, we comment on differences between effects where evidence of statistical significance is stronger (*p* <.01) than others (*p* <.05), calling for caution when interpreting results where alpha levels are in the 0.01 and 0.05 range. Ultimately, however, all effects discussed meet or exceed the smallest effect size of interest noted below.

Cohen’s [76] r thresholds were used to assess between-person effect sizes (i.e., 0.2 to 0.49 = small, 0.5 to 0.79 = medium, ≥ 0.8 = large). For within-person cross-lagged effects, we adopted the empirical benchmark values recommended for RI-CLPM by Orth et al. [77] following their analysis of a quasi-representative sample of 174 psychological study samples (> 300 effect sizes) using this method of analysis: 0.03 (small effect, 25th percentile), 0.07 (medium effect, 50th percentile), and 0.12 (large effect, 75th percentile). This enabled us to consider the relative size of said effects in the context of the broader psychological literature. However, we are mindful that a range of factors (e.g., size of between-person effects, number and nature of covariates, length of lag between data points, and the use of manifest vs. latent measures), can influence the magnitude of cross-lagged and autoregressive effects, meaning that indiscriminate application of empirically-derived thresholds is inadvisable. Moreover, Adachi and Willoughby [78] argue that the degree to which small longitudinal effects are meaningful largely depends on the size of the bivariate correlations for these effects and the stability of the outcome. Thus, we interpret the cross-lagged parameters dynamically in the context of the wider model considering the size of their bivariate correlations and the outcome stability.

Building on the above, we considered any within-person cross-lagged effects equal to or greater than 0.03 to be meaningful (i.e., the smallest effect size of interest in the current study), because seemingly ‘small’ effects can have important cumulative consequences across time; are more likely to be ‘real’ (i.e. robustly and reliably estimated); and, are more consistent with contemporary theoretical perspectives on the multidetermined nature of complex psychological phenomena (i.e., a multitude of diverse proximal and distal factors drive a given psychological construct or health behaviour; accordingly, the influence of any single factor in isolation is likely to be small) [79, 80].

## Results

In this section, we report findings for Model A, noting any instances where these were sensitive to sample inclusion criteria (Model B; full details reported in Appendix 5).

In the data screening step preceding the estimation of the RI-CLPM, we ruled out multicollinearity (see Table 2; Mean VIF = 1.50; Highest VIF (PAT2) = 1.74), and highly-skewed data (see Table A2.1 in Appendix 2). Our analysis of missingness (see Appendix 2) showed that data were missing at random, indicating the need to employ multiple imputation, which was executed as described above (see *Data Analyses*). We also confirmed the longitudinal and gender measurement invariance of the latent mental wellbeing construct (see Table A4.1 in Appendix 4).

**Table 2:**
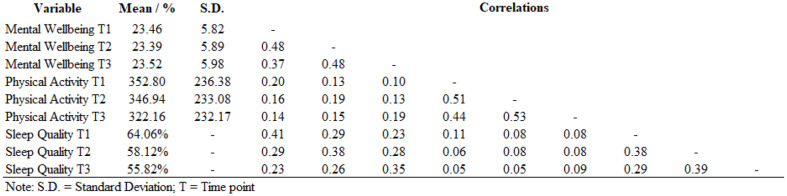
Descriptive statistics and bivariate associations between physical activity, sleep quality, and mental wellbeing

### Structural (in)variance by gender (hypothesis H6)

We found evidence of structural variance by gender (Δ𝜒2 = 75, Δ𝑑𝑓= 30, *p* <.001), indicating that the within-person associations among the three variables of interest and autoregression relationship within each varied between boys and girls, supporting H6. Consequently, RI-CLPMs were conducted separately by gender. The multigroup RI-CLPM model showed good fit to the data: 𝜒2 = 6989.63; *df* = 841; RMSEA = 0.023; CFI = 0.986, TLI = 0.986; SRMR = 0.023. Results are reported in Table 3, which shows the standardised coefficients and p-values for each cross-lagged and autoregressive path for girls and boys. Model fit was not sensitive to sample inclusion criteria, with no substantively different results produced in Model B (see Appendix 5).

### Autoregressive effects (hypotheses H4A, H4B, H4C)

We found evidence of stability for all within-person constructs, meaning that H4A, H4B, and H4C were supported. For boys, the largest effects were observed for sleep quality (H4B: β = 0.45 (*p* <.001) at T1 → T2 and β = 0.48 (*p* <.001) at T2 → T3) followed by mental wellbeing (H4C: β = 0.18 (*p* <.001) at T1 → T2 and β = 0.23 (*p* <.001) at T2 → T3) and physical activity (H4A: β = 0.16 (*p* <.001) at T1 → T2 and β = 0.19 (*p* <.001) at T2 → T3).

For girls, autoregressive effects for sleep quality (and, consequently, differences between this and mental wellbeing and physical activity) were smaller than for boys, as indicated by the Wald test results comparing the unconstrained model with the model where these paths were constrained to be equal across girls and boys (Δ𝜒2 = 13.776, Δ𝑑𝑓= 1, *p* =.000 in T1→T2; and Δ𝜒2 = 14.88, Δ𝑑𝑓= 1, *p* =.000 in T2→ T3). Specifically, for girls, effects for sleep quality (H4B: β = 0.16 (*p* <.001) at T1 → T2 and β = 0.23 (*p* <.001) at T2 → T3) were similar in size to those for mental wellbeing (H4C: β = 0.19 (*p* <.001) at T1 → T2 and β = 0.22 (*p* <.001) at T2-T3). In contrast, autoregressive effects for physical activity were slightly smaller (H4A: β = 0.15 (*p* <.001) at T1 → T2 and β = 0.17 (*p* <.001) at T2 → T3). Findings for boys and girls were not sensitive to sample inclusion criteria, with no substantively different results produced in Model B.

### Concurrent relationships (no hypotheses)

Concurrent relationships across the three constructs within individuals were statistically significant (Table 4). At each timepoint, mental wellbeing exhibited positive correlations with sleep quality for both boys and girls. Mental wellbeing also exhibited positive correlations with physical activity at each time point for both boys and girls. Finally, physical activity exhibited positive correlations with sleep quality for boys at T3 only, and girls at T1 and T3 only. Again, for all these effects, results were not substantially different in Model B.

Regarding between-person differences, we found statistically significant associations between the random intercept latent factors. The relationship between sleep quality and mental wellbeing was especially pronounced (for boys, *r* =.91, *p* <.001; for girls, *r* =.70, *p* <.001). Other associations among random intercepts also showed significant but smaller effect sizes for both genders: sleep quality and physical activity (for boys, *r* =.27, *p* =.004; for girls, *r* =.17, *p* <.001); and physical activity and mental wellbeing (for boys, *r* =.31, *p* <.001; for girls, *r* =.27, *p* <.001). These results were not sensitive to sample inclusion criteria (Model B).

### Cross-lagged effects (hypotheses H1A, H1B, H2A, H2B, H3A, H3B)

Several cross-lagged pathways were statistically significant. This includes the following:

#### Sleep quality predicts later mental wellbeing (H2B)

This hypothesis was fully supported for both boys (β = 0.07 (*p* <.05) at T1→T2, and β = 0.08 (*p* <.01) at T2→T3) and girls (β = 0.08 (*p* <.05) at T1→T2, and β = 0.05 (*p* <.05) at T2→T3), with Wald test results suggesting no gender differences at T1→T2 (Δ𝜒2 = 0.049, Δ𝑑𝑓= 1, *p* =.824) and T2→T3 (Δ𝜒2 = 0.562, Δ𝑑𝑓= 1, *p* =.454).

These findings were somewhat sensitive to sample inclusion criteria. In Model B, only the T2→T3 path was statistically significant for boys (β = 0.08, *p* <.05), and only the T1→T2 path was statistically significant for girls (β = 0.09, *p* <.05), although Wald test results indicated no gender differences at T1→T2 (Δ𝜒2 = 0.102, Δ𝑑𝑓= 1, *p* =.750) or T2→T3 (Δ𝜒2 = 0.913, Δ𝑑𝑓= 1, *p* =.339).

#### Mental wellbeing predicts later sleep quality (H3B)

This was partially supported for girls, with statistically significant effects observed only at T1→T2 (β = 0.11, *p* <.05). Consistent with this, the Wald test results showed that constraining this path to be equal for girls and boys resulted in worse fit (Δ𝜒2 = 6.77, Δ𝑑𝑓= 1, *p* =.009), suggesting that it differed by gender. This finding was not sensitive to sample inclusion criteria.

#### Mental wellbeing predicts later physical activity (H3A)

No statistically significant effects were observed in Model A, but partial support for the hypothesis was observed for boys at T2→T3 (β = 0.05, *p* <.05) in Model B (i.e., those who participated in at least two survey waves). However, Wald test results in Model B showed no evidence of gender differences (Δ𝜒2 = 2.487, Δ𝑑𝑓= 1, *p* =.115).

All other cross-lagged effects were non-significant, meaning that the remaining hypotheses for said effects (H1A, H1B, H2A, H3A) were rejected.

#### Physical activity, sleep quality, and mental wellbeing share a reciprocal relationship over time (H5)

This hypothesis was partially supported in girls, for whom we found a reciprocal relationship over time between sleep quality and mental wellbeing at T1→Τ2. Thus, sleep quality predicted later mental wellbeing, and mental wellbeing predicted later sleep quality (SL→WB β = 0.08, *p* <.05; WB→SL β = 0.11, *p* <.01). These findings were not sensitive to sample inclusion criteria.

**Table 3:**
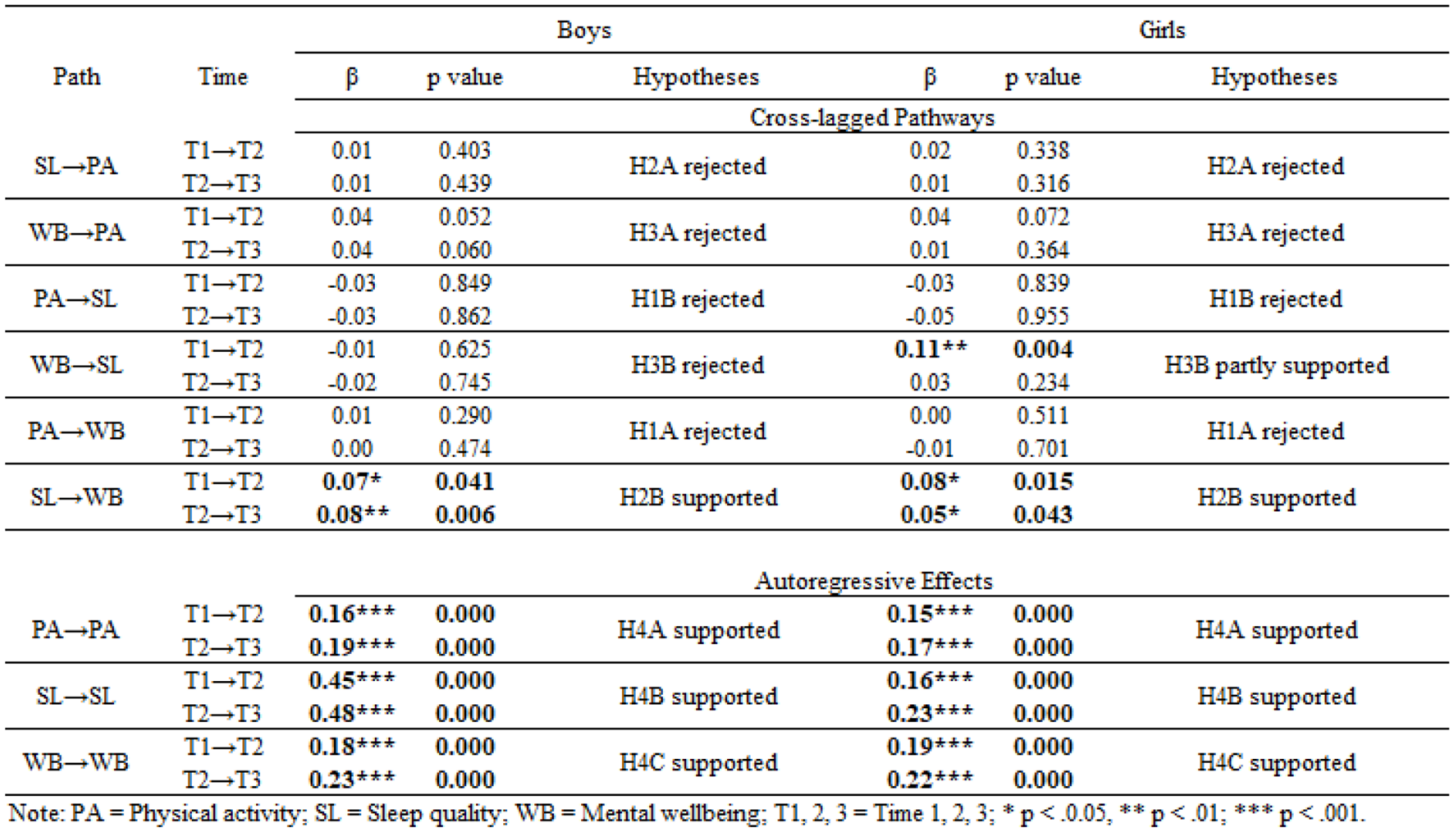
Developmental cascades model showing longitudinal reciprocal relationships between physical activity, sleep quality, and mental wellbeing (model A)

**Table 4:**
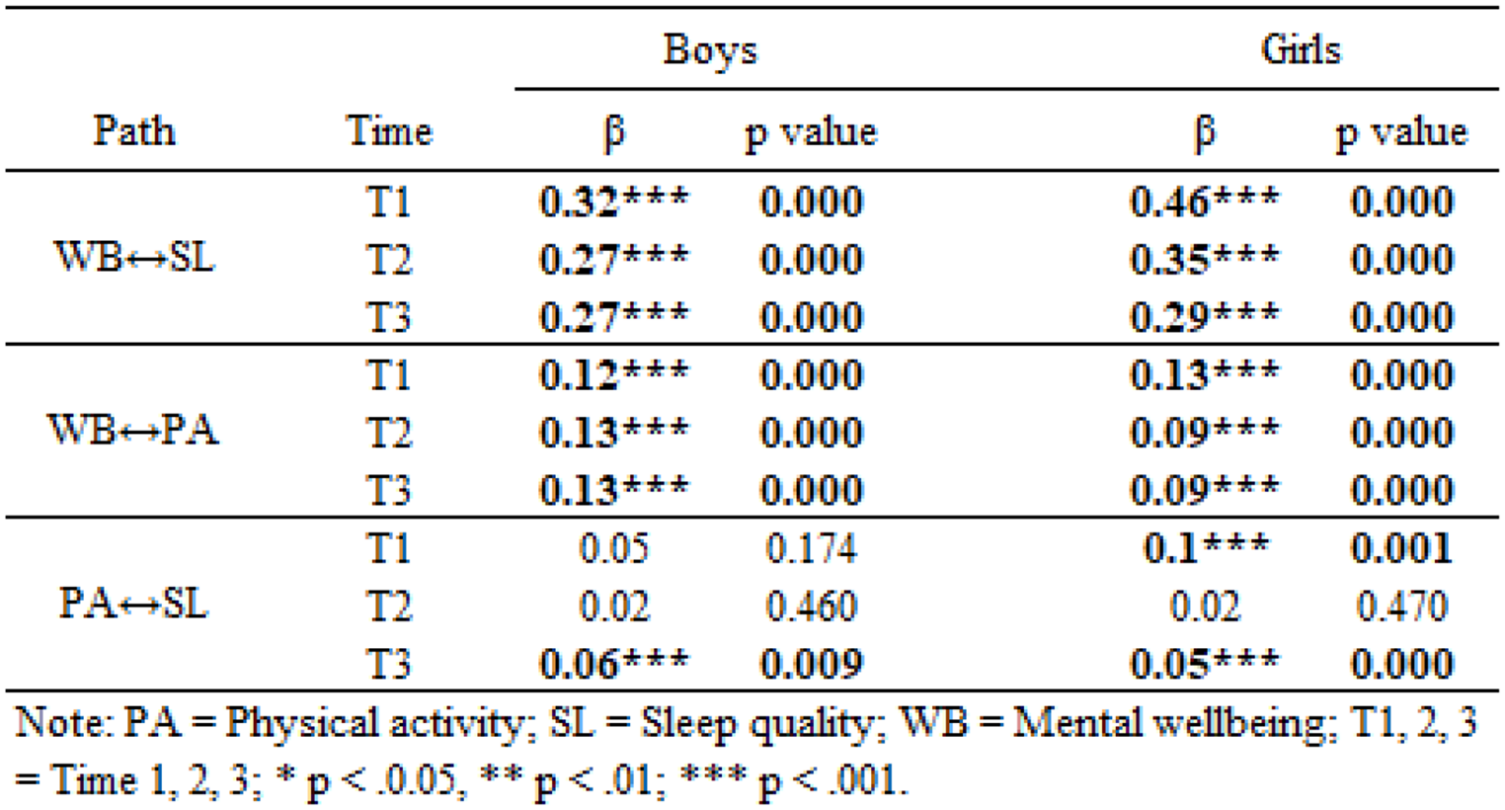
Model A. Concurrent associations across mental wellbeing, physical activity, and sleep quality

## Discussion

The aim of this study was to investigate the longitudinal associations between sleep, physical activity and mental wellbeing during the critical transition from early to middle adolescence, and to determine whether these relationships varied by gender. To address this important developmental cascade research problem, we used RI-CLPM to distinguish between- from within-person effects, while controlling for socio-demographic characteristics (FSM, SEND, and ethnicity) known to explain variance in our focal outcomes [57–59]. RI-CLPM was applied to three annual waves of data from a sample of *N* = 27,949 adolescents (aged 12/13 at T1) in Greater Manchester, England.

We found that developmental cascade pathways varied by gender, with stronger evidence for some effects (*p* <.01) than others (*p* <.05). Specifically, better sleep quality led to higher mental wellbeing for both girls (*p* <.05 at T1→T2 and T2→T3) and boys (*p* <.05 at T1→T2 and *p* <.01 at T2→T3), including small-to-moderate effects for both genders; higher mental wellbeing led to better sleep quality for girls only (at T1→T2; *p* <.01; moderate effects); and sleep quality and mental wellbeing were reciprocally associated over time at T1→Τ2 for girls (*p* < 05; moderate effects). Most findings were not sensitive to sample inclusion criteria. A noteworthy exception is that, in contrast to our main analysis (Model A), higher mental wellbeing led to higher levels of physical activity for boys (*p* <.05 at T2→T3; small effects) in Model B (i.e., participants with at least two waves of data), although Wald test results show no gender differences in this association, meaning that this finding should be interpreted with caution.

### Gender-specific developmental cascades between sleep, physical activity and mental wellbeing (hypothesis 6)

We start our discussion with Hypothesis 6 as this affects how all other findings are reported. We found evidence that the within-person cross-lagged effects between our three focal variables and autoregression relationships within each varied by gender. To date, this issue has not been consistently explored or reported in the prior literature, with very few studies having formally tested for structural variance by gender (e.g., via multigroup analyses). One notable exception, Graupensperger, Sutcliffe, and Vella’s study of longitudinal relations between sports participation and mental health in Australian adolescents [37], reported findings in accordance with our own. Thus, the accumulated evidence to date supports the idea of structural gender differences in the temporal relationships between wellbeing and health behaviours/outcomes. However, this needs to be replicated and confirmed in future research, given the current reliance of most studies on simply assuming gender differences exist and therefore analysing data for boys and girls separately without clear theoretical or empirical justification, leading to inconsistent findings [26, 81, 82].

More broadly, our findings contribute to the growing evidence base regarding the role of gender in developmental cascades during childhood and adolescence. For example, research by Panayiotou and Humphrey [83] examined longitudinal pathways between internalising symptoms, externalising problems, and academic attainment in middle childhood, finding evidence of structural variance by gender. This emergent evidence base supports the proposition that gender socialisation and intensification processes alter developmental cascade process, such that the incoming and outgoing influences pertaining to mental health and wellbeing are of a different nature and/or magnitude between boys and girls. In the subsections that follow, we explore this notion in more detail.

### The influence of physical activity on later sleep and mental wellbeing (hypothesis 1)

The prediction that higher levels of physical activity would lead to higher mental wellbeing (H1A) was not supported either for girls or boys, which contrasts with findings from a systematic review and a meta-analysis that found small longitudinal associations between physical activity and fewer depressive symptoms [29, 30]. This may reflect our focus on mental wellbeing as opposed to depression, for which physical activity perhaps yields a stronger beneficial effect (i.e., the endorphins hypothesis, which posits that exercise increases the secretion of endogenous opioid peptides in the brain, reducing pain and generating euphoria) [84].

We also did not find support for the prediction that higher levels of physical activity would lead to better sleep quality either for boys or girls (H1B). This contrasts with findings by Pesonen et al. in their adult study of the relationship between physical activity and sleep. Those authors found a small within-person effect demonstrating that higher vigorous physical activity on a given day was associated with shorter sleep duration the next night [54]. This divergence of findings may be due to differences in age group (adults vs. adolescents), sleep measure (i.e. objective measures of hours of sleep vs. the feeling of having had enough sleep to be awake and concentrate at school), and/or physical activity measure (vigorous physical activity during the day vs. hours per week being physically active). In addition, we particularly highlight differences in measurement lags (one day vs. one year), as the (physiological) impact of physical activity on sleep is probably more immediate (i.e. days or weeks rather than a year) and, therefore, more likely to be detectable across shorter lags.

### The influence of sleep on later mental wellbeing and physical activity (hypothesis 2)

Our prediction that better sleep quality would lead to higher levels of later physical activity (H2A) was not supported for either gender. Although the literature studying these associations longitudinally is scarce, our findings contrast with those of Semplonius and Willoughby [53], who found that better sleep quality predicted increased physical activity (albeit indirectly, through interim emotion regulation) in a sample of university students (using CLPM), and Pesonen et al. [54], who found that both sleep duration and quality predicted later vigorous physical activity in adults (using RI-CLPM). Apart from differences in measures and time lags, the common feature of these two studies potentially explaining the lack of convergence with our findings is the use of adult samples, which immediately prompts consideration of developmental differences. Both sleep duration [85] and physical activity levels [86, 87] change significantly from adolescence to adulthood, and it is possible that the relationship between them also evolves over time.

In direct contrast, the hypothesis that better sleep quality would predict higher later mental wellbeing (H2B) was fully supported for boys and girls. Observed effects were in the small (i.e., β = 0.05, *p* <.05, girls, T2→T3) to medium (i.e., β = 0.08, *p* <.01, boys, T2→T3) range in the empirical distribution of cross-lagged effects [77], and were somewhat sensitive to sample inclusion criteria (i.e., effects replicated at T2→T3 (but not T1→T2) for boys; and, at T1→T2 (but not T2→T3) for girls). Our findings align with previous longitudinal studies that identified sleep difficulties in adolescence as predictors of later psychiatric issues [40], and sleep duration as a predictor of subjective well-being [41]. However, unlike some earlier research [42, 43], we did not observe gender differences in this association. Collectively, findings pertaining to H2B provide robust evidence of the importance of sleep for later wellbeing of both boys and girls, a point to which we return when considering the implications of the current study (see *Implications* section below).

### The influence of mental wellbeing on later sleep and physical activity (hypothesis 3)

The hypothesis that higher mental wellbeing would predict higher levels of later physical activity (H3A) was rejected in our main analysis (Model A). However, it was partly supported for boys in Model B at T2→T3, with a small observed effect size (β = 0.05; *p* <.05), relative to the empirical distribution of cross-lagged effects [77] (i.e. just above the 25th percentile and the smallest effect size of interest in the current study). This sensitivity analysis finding aligns with those of studies that report lower levels of physical activity are predicted by prior levels of depressive symptoms [31, 34] and lower health-related quality of life [32]. However, we found no WB→PA association for girls, contradicting earlier research on the longitudinal links between physical activity and depression levels in female samples [33]. Collectively, the lack of support for WB→PA cross-lagged effects in both girls and boys in our main analysis (and girls in our sensitivity analysis) may, as above (see discussion of Hypothesis 1) reflect our focus on mental wellbeing as opposed to depression, which likely has significantly more deleterious effects on later physical activity [88].

The prediction that higher mental wellbeing would lead to better sleep quality one year later (H3B) was partly supported, but only for girls (at T1→T2). The observed effect was moderate-to-large (β = 0.11; *p* <.05), sitting just under the 75th percentile in the empirical distribution of cross-lagged effects [77], and was robust to changes in model specification (i.e., replicated in Model B). Our finding is in line with those of Kelly and El-Sheikh [49], whose CLPM study provided evidence that both internalising and externalising symptoms predict later sleep problems in children (albeit without exploring gender differences). However, it contrasts with Narmandakh et al.‘s [50] study, which employed RI-CLPM and found no longitudinal link between anxiety symptoms and the later onset of sleep problems in adolescence. It is unlikely that these contrasting findings result from measurement differences (i.e., mental wellbeing as opposed to symptoms, and sleep quality as opposed to problems) given the alignment with Kelly and El-Sheikh [49]. Accordingly, more research is needed to further elucidate if/how wellbeing influences later sleep, and given our findings, the potential moderating role played by gender.

### Temporal stability of sleep, physical activity and wellbeing (hypothesis 4)

Consistent with developmental cascades theory [1], we found stability/autoregressive effects for sleep quality, physical activity, and mental wellbeing, with notable gender differences. For girls, the autoregressive effects were of similar magnitude across all three variables (i.e., β range 0.15 to 0.22), with mental wellbeing and physical activity effects comparable to those observed in boys (i.e., β range 0.15 to 0.23). However, the autoregressive effects for sleep quality in boys (β range 0.45 to 0.47) were more than twice as large as those in girls (β range 0.16 to 0.23), for which there are important implications discussed in more detail later (see *Implications* subsection).

### Reciprocal relationships between sleep, physical activity and wellbeing over time (hypothesis 5)

Of the three potential reciprocal relationships (i.e., SL↔WB, SL↔PA, PA↔WB), we found partial evidence only for the association between sleep and wellbeing, specifically for girl, at T1→Τ2. These findings, which were not sensitive to sample inclusion criteria (i.e., replicated in Model B), align with prior evidence of temporal reciprocity between sleep disturbances or duration and anxiety and/or depression in adolescents [44–47, 49], and contribute to the evidence base by addressing methodological limitations that have previously hindered firm conclusions from being drawn [48], demonstrating that this relationship extends to mental wellbeing, and providing robust indications in support of some prior work that it is gendered in nature [47]. The timing of this reciprocal association (specifically, from age 12/13 to 13/14) and the fact that it was identified only for girls is particularly noteworthy, given existing evidence regarding peak age of onset of lifetime mental health disorders at age 14.5 [89] and the gendered prevalence of internalising problems [90] and related decline in wellbeing [91].This prompts consideration of the preventative potential of improving sleep quality for girls during the transition from early- to mid-adolescence (see *Implications*).

### Concurrent relationships (no hypotheses)

We found consistent associations whereby those who reported getting enough sleep tended to experience higher levels of mental wellbeing and report undertaking more physical activity, and those reporting more physical activity tended to experience higher levels of mental wellbeing. These effects were mostly small (except for the relationship between sleep and wellbeing) [76], did not vary by gender, and were insensitive to sample inclusion criteria (Model B). Although our primary interest in within-person processes means that we made no specific predictions regarding these between-person differences, these still warrant mention given that they help to determine who would likely benefit from intervention [17, 19], while also helping to contextualise our focal cross-lagged effects. With regard to the latter point, we argue that the within-person effects noted in preceding sections are all the more salient given their presence in the context of significant between-person and autoregressive effects.

### Strengths and limitations

The current study has several strengths that prompt confidence in our results. We used a very large sample, nearly twice the size of the largest in Orth et al.‘s [77] meta-analysis. Our longitudinal design and use of an advanced statistical method (RI-CLPM) that allowed us to separate differences between individuals and changes within individuals (while accounting for the influence of relevant covariates) increased the accuracy and precision of our estimates. The analysis was pre-registered, informed by relevant theory and evidence on developmental processes, and considered the sensitivity of findings to sample inclusion criteria (i.e., including participants who took the survey at least once, Model A, or at least twice, Model B). Missing data were accounted for using multiple imputation.

However, our sample was not nationally representative. Greater Manchester, where the study took place, has higher levels of socio-economic deprivation and more ethnic diversity than the rest of England. This means that we should be cautious with regard to generalisability (although we did of course adjust for ethnicity and free school meal eligibility in our analysis). In terms of measurement, our three main variables (sleep quality, physical activity, and mental wellbeing) were all self-reported. This may have affected the strength of the links we observed due to common method variance. However, much of this is likely captured by the between-person effects in the RI-CLPM, which accounts for stable differences between individuals. It is also important to note that self-reporting is arguably the optimal method for measuring constructs like mental wellbeing [64], and so using multiple sources of information (e.g. parents, teachers) may not necessarily be better in this case. However, there are clearly more objective and accurate methods for capturing both sleep quality and physical activity (e.g., accelerometers and sleep-tracking devices often found in so-called ‘wearables’) [92, 93], though of course their use would have been impractical in a study as large as #BeeWell without considerable additional resource. Finally, as noted above, the use of annual measurement lags in #BeeWell may have masked more immediate effects, particularly of physical activity.

### Implications

We start with the implications of findings that led to the rejection of hypotheses. For those null results involving mental wellbeing (e.g. H1A), the plausibility of the null hypothesis has important implications regarding the distinction between this and related constructs, such as depression. For example, the fact that physical activity did not lead to higher mental wellbeing in our study, but has been shown to consistently predict fewer depressive symptoms in other work [29, 30], implies that its cascading benefits may be confined to reducing later distress and do not extend to feeling good and functioning well. For those null results involving physical activity and sleep (e.g., H2A), the plausibility of the null hypothesis prompts consideration that the salience of these health behaviours varies across different developmental phases, given contrasting findings with adult samples [53, 54]. Accordingly, policy and practice responses pertaining to adolescents should target those factors for which we have more robust, consistent evidence of their primacy (e.g., improving sleep quality to promote mental wellbeing, as described below).

However, it is important that these theorisations are empirically tested. This being the case, the main research implication is the need for future work that address the above-noted limitations to elucidate if, how, for whom, and when these predictive associations manifest. For example, a study with more measurement points and shorter lags between them would help to better understand whether physical activity simply does not predict better sleep quality in adolescence (H1B), or only does so within a shorter time horizon than that used here. Similarly, analysis of a longitudinal dataset with measurements throughout adolescence and into adulthood would help to clarify whether the nature and magnitude of temporal associations between sleep and physical activity do indeed evolve over developmental transitions.

In relation to the hypotheses that were supported, our findings highlight the importance of sleep quality as a driver of wellbeing in the crucial transition from early- to mid-adolescence. It is therefore important that young people are empowered with the knowledge, practices and benefits of good sleep habits, in addition to raising awareness of the immediate and longer-term consequences of sleep deprivation [94]. The current evidence base is promising in this regard. For example, Illingworth et al. reported on ‘Teensleep’: a novel, teacher-led programme, comprising ten flexibly delivered lessons (using cognitive behavioural principles focusing on the science of sleep, sleep hygiene, and stress management), which led to improvements in sleep knowledge, quality, and hygiene [95]. This illustrative example reflects broader meta-analytic evidence that such interventions appear to be effective, but that further high-quality randomised trials are needed [96]. Beyond direct intervention, better consolidated evidence regarding which factors protect or harm adolescent sleep will also be helpful in order to help them strike a healthy balance in their lives. This is particularly the case in relation to factors such as social media use and videogaming, which provide important sources of connection and catharsis, but also interfere with sleep when used to excess [97].

Extending the above, the gender-specific reciprocal association between sleep and wellbeing identified at T1→Τ2 indicates that efficacious interventions targeting better sleep quality for girls could help to reduce the gender gap in mental health and wellbeing that grows during this development period [90, 91]. Our autoregression findings (Hypothesis 4) revealed considerably lower temporal stability in sleep quality for girls compared to boys, indicating that this may be an amenable avenue for intervention. As above though, one would need to consider the evidence regarding which factors protect or harm sleep of adolescent girls in particular (e.g., menstrual problems) [98], or those that are common but for which the exposure is higher for girls (e.g., excessive social media use) [99], or indeed where the exposure may be similar but the magnitude of its effects may differ by gender (e.g., bullying victimisation) [100]. However, consideration also needs to be given to the fact that for girls, wellbeing influenced later sleep. Accordingly, emphasis on sleep *and* wellbeing promotion for girls may be fruitful. To this end, relaxation practices (e.g., deep breathing, progressive muscle relaxation) offer particular promise given the evidence of their potential to improve both of these important outcomes [101].

## Conclusion

This study addressed a significant developmental cascade research problem by testing a series of theory-driven hypotheses to explore how the interaction between sleep, physical activity, and wellbeing evolves over time within individuals during the crucial transition from early- to mid-adolescence, and how this varies by gender. Our findings revealed that sleep was reciprocally associated with wellbeing for girls but not boys (for whom sleep predicted wellbeing but not the other way around); and, wellbeing positively predicted later physical activity for boys but not girls (though only in our pre-planned sensitivity analysis). Our results highlight the importance of sleep as a driver of adolescent wellbeing, and the role of gender in developmental cascade processes.

## Electronic supplementary material

Below is the link to the electronic supplementary material.

Supplementary Material 1

Supplementary Material 2

## Footnotes

1 The linked administrative data used refers to ‘female’ and ‘male’, language which is recognised as being more strongly associated with biological sex than gender. We use ‘girls’ and ‘boys’ here in alignment with recommended nomenclature for gender [102], and given that our hypothesis pertains to participants’ gender (i.e., social construction and intensification of attitudes, feelings and behaviours based on a person’s biological sex by family, schools, peer groups, and the media) as opposed to their sex. In other words, it is our assumption that any differences observed in within-person effects result from gender socialisation and intensification as opposed to underpinning biological and/or chromosomal factors.

2
https://osf.io/preprints/osf/wkytd_v1.
