# Correction: Longitudinal relationships across sleep, physical activity, and mental wellbeing in early-to-mid-adolescence: a developmental cascades investigation

**DOI:** 10.1007/s11136-025-03921-2

**Published:** 2025-03-20

**Authors:** Jose Marquez, Margarita Panayiotou, Reihaneh Farzinnia, Qiqi Cheng, Neil Humphrey

**Affiliations:** Manchester Institute of Education, University of Manchester, Manchester, M13 9PL UK

**Correction to: Quality of Life Research**

10.1007/s11136-025-03894-2

In the original published article, author’s name Margarita Panayiotou was incorrectly written as Margarita Panayioutou.

Also footnote 2 “https://osf.io/preprints/osf/wkytd_v1” is missing in the section “**Data analyses**”.

The original article has been corrected.

