# Supplementary material for: Longitudinal relationships across sleep, physical activity, and mental wellbeing in early-to-mid-adolescence: a developmental cascades investigation
Source: Qual Life Res. 2025 Jan 28;34(5):1233–50. doi: 10.1007/s11136-025-03894-2 (PMC12064606; doi:10.1007/s11136-025-03894-2)
Supplement: Supplementary file 1 — Supplementary Material 1 [file 11136_2025_3894_MOESM1_ESM.docx]

**Supplementary Materials**

**Appendix 1: MPlus Syntax for the analysis**

**Syntax: Random-intercept panel model in Mplus**

**TITLE:** PA-SL-WB;

**DATA**: file is missimp(WB-PA-SL)100_list.dat;

**TYPE**=IMPUTATION;

**VARIABLE:**

**NAMES** =

SLT1 SLT2 SLT3

WB1T1 WB2T1 WB3T1 WB4T1 WB5T1 WB6T1 WB7T1

WB1T2 WB2T2 WB3T2 WB4T2 WB5T2 WB6T2 WB7T2

WB1T3 WB2T3 WB3T3 WB4T3 WB5T3 WB6T3 WB7T3

ETH FSM SEN PAT1 PAT2 PAT3 SCHOOL SEX;

**USEVARIABLES** =

WB1T1 WB2T1 WB3T1 WB4T1 WB5T1 WB6T1 WB7T1

WB1T2 WB2T2 WB3T2 WB4T2 WB5T2 WB6T2 WB7T2

WB1T3 WB2T3 WB3T3 WB4T3 WB5T3 WB6T3 WB7T3

SLT1 SLT2 SLT3

ETH FSM SEN SEX

PAT1 PAT2 PAT3;

**CATEGORICAL** =

SLT1 SLT2 SLT3

WB1T1 WB2T1 WB3T1 WB4T1 WB5T1 WB6T1 WB7T1

WB1T2 WB2T2 WB3T2 WB4T2 WB5T2 WB6T2 WB7T2

WB1T3 WB2T3 WB3T3 WB4T3 WB5T3 WB6T3 WB7T3;

**MISSING** =.;

**CLUSTER** = SCHOOL;

!ADDED TO EXPLORE THE EQUALITY OF PATHS USING WALD TEST BETWEEN GENDERS

**GROUPING** = SEX (0 = male 1 = female);

**DEFINE**:

PAT1=PAT1/100;

PAT2=PAT2/100;

PAT3=PAT3/100;

! Continous variables can be rescaled to /10 in

! order to get a variance! under 10 and thus avoid convergence issues

**ANALYSIS**:

**TYPE** = COMPLEX;

**ESTIMATOR** = WLSMV;

**PARAMETERIZATION** = THETA;

**MODEL** = NOCOVARIANCES;

**MODEL:**

! FACTOR MODELS for WB at 3 waves

WBT1 by WB1T1* WB2T1 WB3T1@1 WB4T1 WB5T1 WB6T1 WB7T1 (L1-L7);

WBT2 by WB1T2* WB2T2 WB3T2@1 WB4T2 WB5T2 WB6T2 WB7T2 (L1-L7);

WBT3 by WB1T3* WB2T3 WB3T3@1 WB4T3 WB5T3 WB6T3 WB7T3 (L1-L7);

[WBT2* WBT3*];

! RESIDUAL COVARIANCES

WB1T1 with WB1T2 WB1T3;

WB1T2 with WB1T3;

WB2T1 with WB2T2 WB2T3;

WB2T2 with WB2T3;

WB3T1 with WB3T2 WB3T3;

WB3T2 with WB3T3;

WB4T1 with WB4T2 WB4T3;

WB4T2 with WB4T3;

WB5T1 with WB5T2 WB5T3;

WB5T2 with WB5T3;

WB6T1 with WB6T2 WB6T3;

WB6T2 with WB6T3;

WB7T1 with WB7T2 WB7T3;

WB7T2 with WB7T3;

! EQUAL THRESHOLDS

[WB1T1$1 WB1T2$1 WB1T3$1](thr11);

[WB2T1$1 WB2T2$1 WB2T3$1](thr21);

[WB3T1$1 WB3T2$1 WB3T3$1](thr31);

[WB4T1$1 WB4T2$1 WB4T3$1](thr41);

[WB5T1$1 WB5T2$1 WB5T3$1](thr51);

[WB6T1$1 WB6T2$1 WB6T3$1](thr61);

[WB7T1$1 WB7T2$1 WB7T3$1](thr71);

[WB1T1$2 WB1T2$2 WB1T3$2](thr12);

[WB2T1$2 WB2T2$2 WB2T3$2](thr22);

[WB3T1$2 WB3T2$2 WB3T3$2](thr32);

[WB4T1$2 WB4T2$2 WB4T3$2](thr42);

[WB5T1$2 WB5T2$2 WB5T3$2](thr52);

[WB6T1$2 WB6T2$2 WB6T3$2](thr62);

[WB7T1$2 WB7T2$2 WB7T3$2](thr72);

[WB1T1$3 WB1T2$3 WB1T3$3](thr13);

[WB2T1$3 WB2T2$3 WB2T3$3](thr23);

[WB3T1$3 WB3T2$3 WB3T3$3](thr33);

[WB4T1$3 WB4T2$3 WB4T3$3](thr43);

[WB5T1$3 WB5T2$3 WB5T3$3](thr53);

[WB6T1$3 WB6T2$3 WB6T3$3](thr63);

[WB7T1$3 WB7T2$3 WB7T3$3](thr73);

[WB1T1$4 WB1T2$4 WB1T3$4](thr14);

[WB2T1$4 WB2T2$4 WB2T3$4](thr24);

[WB3T1$4 WB3T2$4 WB3T3$4](thr34);

[WB4T1$4 WB4T2$4 WB4T3$4](thr44);

[WB5T1$4 WB5T2$4 WB5T3$4](thr54);

[WB6T1$4 WB6T2$4 WB6T3$4](thr64);

[WB7T1$4 WB7T2$4 WB7T3$4](thr74);

!!!!!!!!!!!!!!!!!!!!!!!!!!!!!!

!!!!!!!!!!!!RI-CLPM!!!!!!!!!!!

!!!!!!!!!!!!!!!!!!!!!!!!!!!!!!

! Create three individual factors (random intercepts)

RI_PA by PAT1@1 PAT2@1 PAT3@1;

RI_SL by SLT1@1 SLT2@1 SLT3@1;

RI_WB by WBT1@1 WBT2@1 WBT3@1;

! Create within-person centered variables

W_PAT1 by PAT1@1;

W_PAT2 by PAT2@1;

W_PAT3 by PAT3@1;

W_SLT1 by SLT1@1;

W_SLT2 by SLT2@1;

W_SLT3 by SLT3@1;

W_WBT1 by WBT1@1;

W_WBT2 by WBT2@1;

W_WBT3 by WBT3@1;

! Constrain the measurement error variances to zero

PAT1-PAT3@0;

SLT1-SLT3@0;

WBT1-WBT3@0;

! Covariates

PAT1-PAT3 on ETH (b1);

PAT1-PAT3 on FSM (b2);

PAT1-PAT3 on SEN (b3);

SLT1-SLT3 on ETH (b5);

SLT1-SLT3 on FSM (b6);

SLT1-SLT3 on SEN (b7);

WBT1-WBT3 on ETH (b9);

WBT1-WBT3 on FSM (b10);

WBT1-WBT3 on SEN (b11);

**MODEL male:**

WB1T2* WB2T2* WB3T2* WB4T2* WB5T2* WB6T2* WB7T2*;

WB1T3* WB2T3* WB3T3* WB4T3* WB5T3* WB6T3* WB7T3*;

SLT1-SLT3@0;

[WBT2* WBT3*];

! *EDITS TO EXPLORE THE EQUALITY OF PATHS USING WALD TEST BETWEEN GENDERS

RI_PA with RI_SL (m1);

RI_PA with RI_WB (m2);

RI_SL with RI_WB (m3);

! *EDITS TO EXPLORE THE EQUALITY OF PATHS USING WALD TEST BETWEEN GENDERS

! Estimate the lagged effects between

! the within-person centered variables

W_PAT2 on W_PAT1 (m4);

W_PAT2 on W_SLT1 (m5);

W_PAT2 on W_WBT1 (m6);

W_SLT2 on W_PAT1 (m7);

W_SLT2 on W_SLT1 (m8);

W_SLT2 on W_WBT1 (m9);

W_WBT2 on W_PAT1 (m10);

W_WBT2 on W_SLT1 (m11);

W_WBT2 on W_WBT1 (m12);

W_PAT3 on W_PAT2 (m13);

W_PAT3 on W_SLT2 (m14);

W_PAT3 on W_WBT2 (m15);

W_SLT3 on W_PAT2 (m16);

W_SLT3 on W_SLT2 (m17);

W_SLT3 on W_WBT2 (m18);

W_WBT3 on W_PAT2 (m19);

W_WBT3 on W_SLT2 (m20);

W_WBT3 on W_WBT2 (m21);

W_SLT1-W_SLT3@1;

! *EDITS TO EXPLORE THE EQUALITY OF PATHS USING WALD TEST BETWEEN GENDERS

! Estimate the covariance between the within-person

! centered variables at the first wave

W_PAT1 with W_WBT1 (m22);

W_PAT1 with W_SLT1 (m23);

W_WBT1 with W_SLT1 (m24);

! Estimate the covariances between the residuals of

! the within-person centered variables (the innovations)

W_PAT2 with W_SLT2 (m25);

W_PAT2 with W_WBT2 (m26);

W_SLT2 with W_WBT2 (m27);

W_PAT3 with W_SLT3 (m28);

W_PAT3 with W_WBT3 (m29);

W_SLT3 with W_WBT3 (m30);

! *EDITS TO EXPLORE THE EQUALITY OF PATHS USING WALD TEST BETWEEN GENDERS

**MODEL female**:

[W_PAT1- W_PAT3@0];

[W_SLT1- W_SLT3@0];

[W_WBT1- W_WBT3@0];

[RI_PA@0];

[RI_SL@0];

[RI_WB@0];

RI_PA with RI_SL (f1);

RI_PA with RI_WB (f2);

RI_SL with RI_WB (f3);

W_PAT2 on W_PAT1 (f4);

W_PAT2 on W_SLT1 (f5);

W_PAT2 on W_WBT1 (f6);

W_SLT2 on W_PAT1 (f7);

W_SLT2 on W_SLT1 (f8);

W_SLT2 on W_WBT1 (f9);

W_WBT2 on W_PAT1 (f10);

W_WBT2 on W_SLT1 (f11);

W_WBT2 on W_WBT1 (f12);

W_PAT3 on W_PAT2 (f13);

W_PAT3 on W_SLT2 (f14);

W_PAT3 on W_WBT2 (f15);

W_SLT3 on W_PAT2 (f16);

W_SLT3 on W_SLT2 (f17);

W_SLT3 on W_WBT2 (f18);

W_WBT3 on W_PAT2 (f19);

W_WBT3 on W_SLT2 (f20);

W_WBT3 on W_WBT2 (f21);

W_PAT1 with W_WBT1 (f22);

W_PAT1 with W_SLT1 (f23);

W_WBT1 with W_SLT1 (f24);

W_PAT2 with W_SLT2 (f25);

W_PAT2 with W_WBT2 (f26);

W_SLT2 with W_WBT2 (f27);

W_PAT3 with W_SLT3 (f28);

W_PAT3 with W_WBT3 (f29);

W_SLT3 with W_WBT3 (f30);

W_SLT1-W_SLT3@1;

**MODEL TEST**:

0 = m1-f1;

0 = m2-f2;

0 = m3-f3;

0 = m4-f4;

0 = m5-f5;

0 = m6-f6;

0 = m7-f7;

0 = m8-f8;

0 = m9-f9;

0 = m10-f10;

0 = m11-f11;

0 = m12-f12;

0 = m13-f13;

0 = m14-f14;

0 = m15-f15;

0 = m16-f16;

0 = m17-f17;

0 = m18-f18;

0 = m19-f19;

0 = m20-f20;

0 = m21-f21;

0 = m22-f22;

0 = m23-f23;

0 = m24-f24;

0 = m25-f25;

0 = m26-f26;

0 = m27-f27;

0 = m28-f28;

0 = m29-f29;

0 = m30-f30;

**output**: tech1 tech2 tech4 stdyx sampstat;

**Note**: to test gender differences for specific paths, we used the same approach to compare a multiple group RI-CLPM with no constraints across the two groups (boys and girls) with another model in which the regression coefficients for each specific path were constrained to be the same for boys and girls using the Wald test chi-square.

**Appendix 2: Data screening**

Data was screened for missing values, skewness and kurtosis (all below the recommended thresholds of 2.0 and 7.0, respectively [103, 104]). Results are reported in Table A2.1. We conducted Little’s test [105] and results indicated that data are not missing completely at random (MCAR; p-value=0.000). Thus, we conducted a binary logistic regression to determine whether data are missing at random (MAR; that is, conditional on other observed variables), finding that missingness is indeed conditional to the four covariates. This is indicated in Table A2.2, where we report odds ratios pertaining to missingness for gender, ethnicity, FSM, and SEN.

**Table A2.1**

Missing Data, Skeweness, and Kurtosis for Mental Wellbeing, Physical Activity, and Sleep Quality


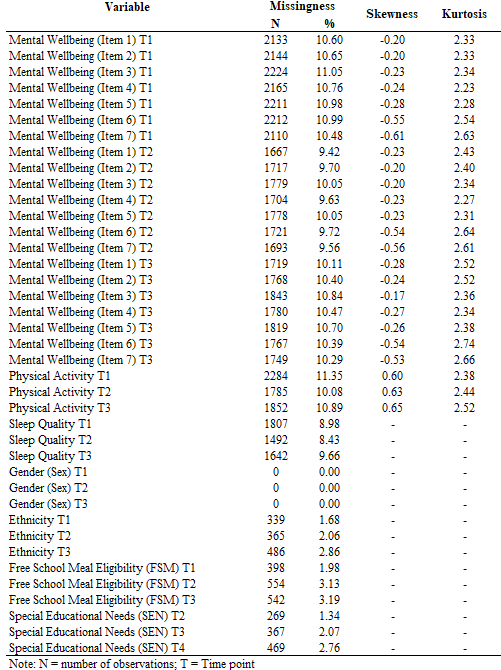


**Table A2.2**

Odds Ratios Pertaining to Missingness for Covariates for Mental Wellbeing, Physical Activity, and Sleep Quality


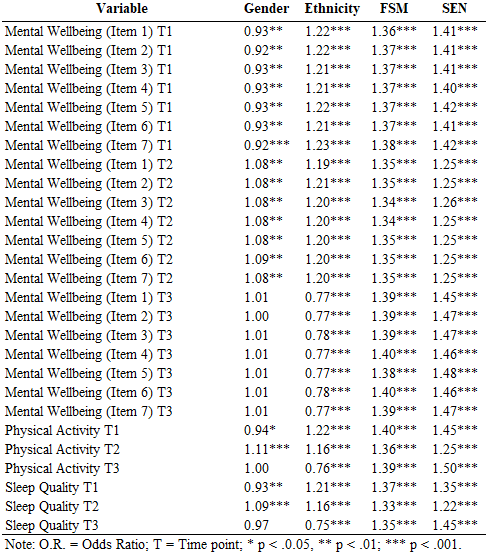


**Appendix 3: Covariate effects on mental wellbeing, physical activity and sleep**

In Stage Submission 1, we considered gender, ethnicity, FSM and SEN as covariates in our RI-CLPM. Table A3.1 and Table A3.2 show the standardised covariates effects on mental wellbeing, physical activity and sleep quality. After finding evidence of gender invariance (see Appendix 4), we ultimately estimated two separate models, one for boys and one for girls, and gender was not used as a covariate.

**Table A3.1**

Standardised covariates effects on mental wellbeing, physical activity and sleep quality, whole sample


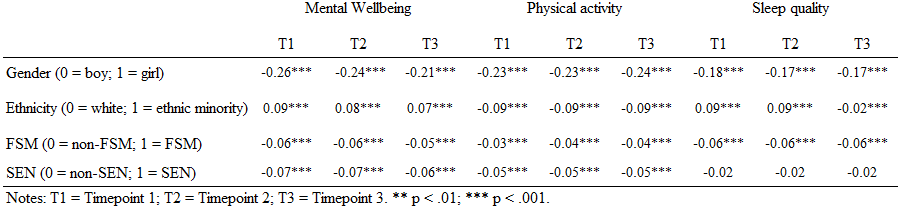


**Table A3.2**

Standardised covariates effects on mental wellbeing, physical activity and sleep quality, boy/girl


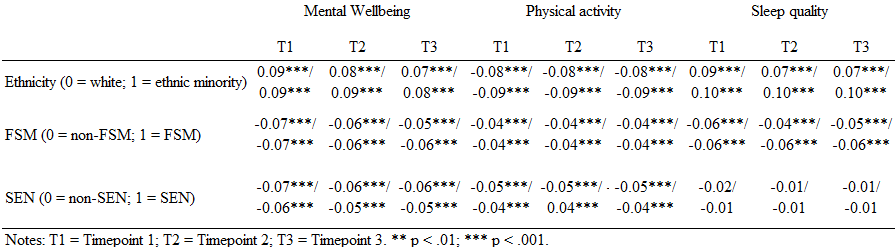


**Appendix 4: Measurement invariance**

Before testing the RI-CLPM model, we tested the longitudinal and gender measurement invariance of mental wellbeing as recommended for ordinal data [106], following a three-step method (baseline models, configural invariance, scalar invariance) using confirmatory factor analysis. Nested model fit indices are reported in Table A4.1 CFI changes are below .01, which supports the assumption of invariance across gender and time [107].

**Table A4.1.**

Gender and longitudinal measurement invariance for mental wellbeing


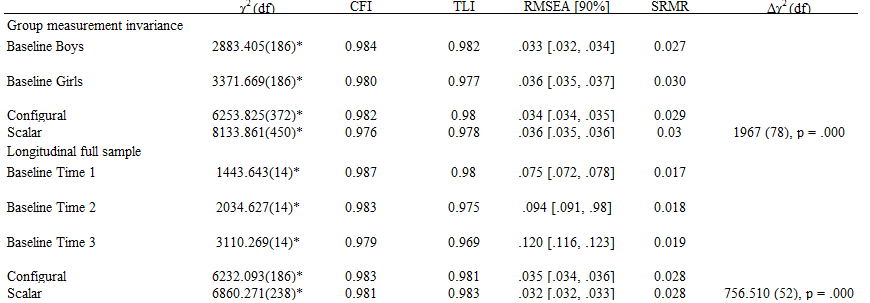


**Appendix 5: Sensitivity analysis (Model B)**

**Table A5.1.**

Developmental cascades model showing longitudinal reciprocal relationships between physical activity, sleep quality, and mental wellbeing (Model B)


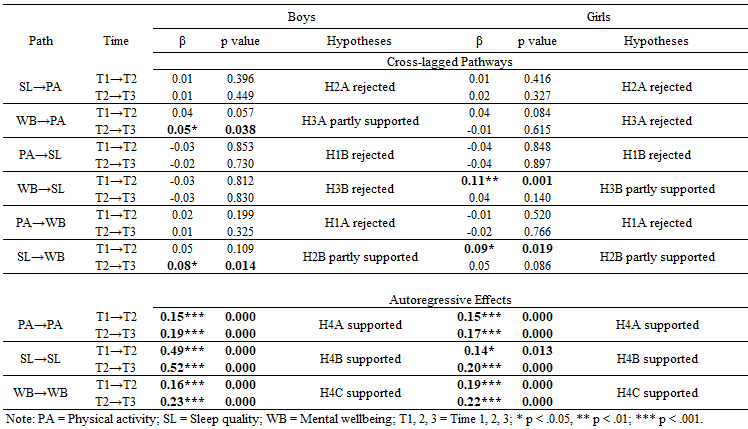


**Appendix 6: Mplus output**

**Model A: Multigroup model (participated in 1 wave at least):**

STANDARDIZED MODEL RESULTS

STDYX Standardization

Two-Tailed Rate of

Estimate S.E. Est./S.E. P-Value Missing

Group MALE

WBT1 BY

WB1T1 0.598 0.007 82.519 0.000 0.405

WB2T1 0.710 0.007 105.883 0.000 0.480

WB3T1 0.725 0.006 111.594 0.000 0.367

WB4T1 0.716 0.007 110.020 0.000 0.458

WB5T1 0.818 0.005 156.440 0.000 0.430

WB6T1 0.632 0.007 94.338 0.000 0.385

WB7T1 0.727 0.006 115.559 0.000 0.414

WBT2 BY

WB1T2 0.667 0.007 96.771 0.000 0.460

WB2T2 0.772 0.006 136.587 0.000 0.515

WB3T2 0.753 0.006 124.946 0.000 0.427

WB4T2 0.745 0.006 122.707 0.000 0.416

WB5T2 0.843 0.005 171.979 0.000 0.420

WB6T2 0.678 0.007 101.699 0.000 0.432

WB7T2 0.761 0.006 134.383 0.000 0.415

WBT3 BY

WB1T3 0.720 0.006 116.754 0.000 0.505

WB2T3 0.824 0.005 158.798 0.000 0.501

WB3T3 0.783 0.005 149.047 0.000 0.362

WB4T3 0.800 0.005 160.025 0.000 0.450

WB5T3 0.868 0.004 206.108 0.000 0.494

WB6T3 0.736 0.006 117.457 0.000 0.498

WB7T3 0.790 0.006 141.296 0.000 0.441

RI_PA BY

PAT1 0.615 0.011 55.733 0.000 0.388

PAT2 0.627 0.013 46.663 0.000 0.290

PAT3 0.632 0.012 53.581 0.000 0.383

RI_SL BY

SLT1 0.460 0.081 5.697 0.000 0.607

SLT2 0.422 0.086 4.904 0.000 0.619

SLT3 0.416 0.085 4.879 0.000 0.627

W_PAT1 BY

PAT1 0.782 0.009 89.970 0.000 0.379

W_PAT2 BY

PAT2 0.772 0.011 70.530 0.000 0.284

W_PAT3 BY

PAT3 0.768 0.010 79.442 0.000 0.377

W_SLT1 BY

SLT1 0.877 0.039 22.581 0.000 0.633

W_SLT2 BY

SLT2 0.897 0.038 23.478 0.000 0.641

W_SLT3 BY

SLT3 0.900 0.037 24.068 0.000 0.649

RI_WB BY

WBT1 0.608 0.018 33.047 0.000 0.568

WBT2 0.584 0.019 30.358 0.000 0.555

WBT3 0.546 0.017 32.444 0.000 0.582

W_WBT1 BY

WBT1 0.784 0.014 55.280 0.000 0.563

W_WBT2 BY

WBT2 0.802 0.014 57.486 0.000 0.549

W_WBT3 BY

WBT3 0.830 0.011 75.459 0.000 0.577

W_PAT2 ON

W_PAT1 0.161 0.025 6.534 0.000 0.542

W_SLT1 0.010 0.041 0.248 0.805 0.709

W_WBT1 0.043 0.027 1.628 0.103 0.600

W_SLT2 ON

W_PAT1 -0.033 0.032 -1.032 0.302 0.630

W_SLT1 0.447 0.062 7.180 0.000 0.646

W_WBT1 -0.011 0.034 -0.320 0.749 0.541

W_WBT2 ON

W_PAT1 0.014 0.026 0.553 0.580 0.619

W_SLT1 0.066 0.038 1.741 0.082 0.596

W_WBT1 0.181 0.033 5.527 0.000 0.555

W_PAT3 ON

W_PAT2 0.194 0.027 7.275 0.000 0.485

W_SLT2 0.007 0.041 0.180 0.857 0.701

W_WBT2 0.040 0.026 1.551 0.121 0.580

W_SLT3 ON

W_PAT2 -0.031 0.029 -1.087 0.277 0.696

W_SLT2 0.476 0.059 8.128 0.000 0.694

W_WBT2 -0.020 0.030 -0.660 0.509 0.637

W_WBT3 ON

W_PAT2 0.001 0.021 0.067 0.947 0.587

W_SLT2 0.084 0.033 2.525 0.012 0.509

W_WBT2 0.227 0.026 8.664 0.000 0.507

WBT1 ON

ETH 0.086 0.007 12.600 0.000 0.237

FSM -0.062 0.006 -9.813 0.000 0.343

SEN -0.065 0.007 -9.451 0.000 0.227

WBT2 ON

ETH 0.082 0.007 12.626 0.000 0.221

FSM -0.060 0.006 -9.855 0.000 0.344

SEN -0.063 0.007 -9.494 0.000 0.229

WBT3 ON

ETH 0.077 0.006 12.431 0.000 0.240

FSM -0.056 0.006 -9.807 0.000 0.342

SEN -0.059 0.006 -9.512 0.000 0.226

PAT1 ON

ETH -0.081 0.007 -12.233 0.000 0.224

FSM -0.035 0.006 -6.216 0.000 0.203

SEN -0.046 0.006 -7.463 0.000 0.226

PAT2 ON

ETH -0.083 0.007 -12.329 0.000 0.225

FSM -0.036 0.006 -6.238 0.000 0.203

SEN -0.047 0.006 -7.404 0.000 0.226

PAT3 ON

ETH -0.083 0.007 -12.348 0.000 0.228

FSM -0.036 0.006 -6.234 0.000 0.205

SEN -0.047 0.006 -7.442 0.000 0.224

SLT1 ON

ETH 0.099 0.009 10.926 0.000 0.269

FSM -0.065 0.008 -7.855 0.000 0.318

SEN -0.019 0.009 -2.227 0.026 0.283

SLT2 ON

ETH 0.090 0.008 11.619 0.000 0.201

FSM -0.060 0.007 -8.044 0.000 0.312

SEN -0.018 0.008 -2.248 0.025 0.273

SLT3 ON

ETH 0.089 0.008 11.566 0.000 0.216

FSM -0.059 0.007 -8.052 0.000 0.307

SEN -0.017 0.008 -2.244 0.025 0.273

RI_PA WITH

RI_SL 0.273 0.114 2.401 0.016 0.643

RI_WB 0.314 0.038 8.345 0.000 0.638

RI_SL WITH

RI_WB 0.913 0.201 4.547 0.000 0.518

W_PAT1 WITH

W_WBT1 0.123 0.025 4.933 0.000 0.575

W_SLT1 0.053 0.039 1.360 0.174 0.658

W_WBT1 WITH

W_SLT1 0.320 0.039 8.176 0.000 0.583

W_PAT2 WITH

W_SLT2 0.020 0.027 0.740 0.460 0.517

W_WBT2 0.126 0.023 5.572 0.000 0.575

W_SLT2 WITH

W_WBT2 0.267 0.026 10.137 0.000 0.472

W_PAT3 WITH

W_SLT3 0.056 0.021 2.607 0.009 0.478

W_WBT3 0.133 0.016 8.228 0.000 0.515

W_SLT3 WITH

W_WBT3 0.267 0.021 12.511 0.000 0.579

WB1T1 WITH

WB1T2 0.266 0.019 14.182 0.000 0.640

WB1T3 0.197 0.021 9.542 0.000 0.650

WB1T2 WITH

WB1T3 0.336 0.020 16.503 0.000 0.661

WB2T1 WITH

WB2T2 0.157 0.019 8.189 0.000 0.555

WB2T3 0.107 0.025 4.269 0.000 0.632

WB2T2 WITH

WB2T3 0.189 0.022 8.506 0.000 0.531

WB3T1 WITH

WB3T2 0.202 0.021 9.794 0.000 0.552

WB3T3 0.170 0.024 7.023 0.000 0.608

WB3T2 WITH

WB3T3 0.228 0.021 10.662 0.000 0.592

WB4T1 WITH

WB4T2 0.129 0.022 5.774 0.000 0.599

WB4T3 0.095 0.025 3.799 0.000 0.642

WB4T2 WITH

WB4T3 0.098 0.025 3.990 0.000 0.656

WB5T1 WITH

WB5T2 0.138 0.026 5.240 0.000 0.557

WB5T3 0.181 0.032 5.640 0.000 0.570

WB5T2 WITH

WB5T3 0.188 0.033 5.704 0.000 0.659

WB6T1 WITH

WB6T2 0.214 0.019 10.973 0.000 0.593

WB6T3 0.172 0.023 7.579 0.000 0.638

WB6T2 WITH

WB6T3 0.230 0.022 10.235 0.000 0.675

WB7T1 WITH

WB7T2 0.191 0.022 8.504 0.000 0.544

WB7T3 0.106 0.024 4.390 0.000 0.554

WB7T2 WITH

WB7T3 0.179 0.025 7.142 0.000 0.631

Means

RI_PA 0.000 0.000 0.000 1.000 0.000

RI_SL 0.000 0.000 0.000 1.000 0.000

RI_WB 0.000 0.000 0.000 1.000 0.000

W_PAT1 0.000 0.000 0.000 1.000 0.000

W_PAT2 0.000 0.000 0.000 1.000 0.000

W_PAT3 0.000 0.000 0.000 1.000 0.000

W_SLT1 0.000 0.000 0.000 1.000 0.000

W_SLT2 0.000 0.000 0.000 1.000 0.000

W_SLT3 0.000 0.000 0.000 1.000 0.000

W_WBT1 0.000 0.000 0.000 1.000 0.000

W_WBT2 0.000 0.000 0.000 1.000 0.000

W_WBT3 0.000 0.000 0.000 1.000 0.000

Intercepts

PAT1 1.621 0.024 67.498 0.000 0.174

PAT2 1.630 0.023 72.086 0.000 0.286

PAT3 1.590 0.023 68.575 0.000 0.216

WBT1 0.000 0.000 0.000 1.000 0.000

WBT2 -0.057 0.019 -3.030 0.002 0.339

WBT3 -0.052 0.021 -2.483 0.013 0.342

Thresholds

WB1T1$1 -1.514 0.023 -66.545 0.000 0.279

WB1T1$2 -0.809 0.016 -49.061 0.000 0.235

WB1T1$3 0.013 0.013 0.968 0.333 0.229

WB1T1$4 0.893 0.017 51.679 0.000 0.299

WB2T1$1 -1.518 0.021 -71.445 0.000 0.270

WB2T1$2 -0.792 0.016 -48.631 0.000 0.187

WB2T1$3 0.048 0.015 3.310 0.001 0.208

WB2T1$4 1.007 0.018 56.303 0.000 0.291

WB3T1$1 -1.623 0.020 -81.663 0.000 0.242

WB3T1$2 -0.800 0.016 -50.141 0.000 0.207

WB3T1$3 0.022 0.015 1.441 0.150 0.225

WB3T1$4 0.972 0.018 54.237 0.000 0.270

WB4T1$1 -1.450 0.022 -64.732 0.000 0.266

WB4T1$2 -0.776 0.017 -44.555 0.000 0.230

WB4T1$3 -0.004 0.015 -0.242 0.809 0.195

WB4T1$4 0.903 0.019 48.725 0.000 0.265

WB5T1$1 -1.597 0.022 -74.125 0.000 0.263

WB5T1$2 -0.858 0.018 -46.747 0.000 0.243

WB5T1$3 -0.058 0.017 -3.438 0.001 0.215

WB5T1$4 0.840 0.019 44.026 0.000 0.254

WB6T1$1 -1.736 0.024 -73.559 0.000 0.300

WB6T1$2 -1.115 0.019 -60.205 0.000 0.255

WB6T1$3 -0.372 0.015 -25.537 0.000 0.244

WB6T1$4 0.560 0.015 37.711 0.000 0.241

WB7T1$1 -1.809 0.024 -76.393 0.000 0.320

WB7T1$2 -1.167 0.019 -61.946 0.000 0.297

WB7T1$3 -0.406 0.016 -25.072 0.000 0.256

WB7T1$4 0.500 0.017 29.966 0.000 0.253

WB1T2$1 -1.625 0.022 -75.087 0.000 0.298

WB1T2$2 -0.869 0.017 -52.622 0.000 0.265

WB1T2$3 0.014 0.014 0.967 0.333 0.228

WB1T2$4 0.958 0.018 53.245 0.000 0.290

WB2T2$1 -1.585 0.022 -73.258 0.000 0.287

WB2T2$2 -0.827 0.017 -49.072 0.000 0.215

WB2T2$3 0.050 0.015 3.306 0.001 0.205

WB2T2$4 1.052 0.019 54.602 0.000 0.267

WB3T2$1 -1.620 0.021 -76.977 0.000 0.343

WB3T2$2 -0.798 0.017 -48.115 0.000 0.285

WB3T2$3 0.022 0.015 1.442 0.149 0.223

WB3T2$4 0.970 0.017 57.402 0.000 0.192

WB4T2$1 -1.449 0.022 -66.549 0.000 0.301

WB4T2$2 -0.775 0.017 -44.932 0.000 0.255

WB4T2$3 -0.004 0.015 -0.243 0.808 0.195

WB4T2$4 0.902 0.018 49.920 0.000 0.260

WB5T2$1 -1.582 0.023 -68.618 0.000 0.309

WB5T2$2 -0.850 0.019 -44.530 0.000 0.279

WB5T2$3 -0.057 0.017 -3.430 0.001 0.217

WB5T2$4 0.832 0.019 44.911 0.000 0.239

WB6T2$1 -1.788 0.023 -78.291 0.000 0.291

WB6T2$2 -1.149 0.019 -61.802 0.000 0.300

WB6T2$3 -0.383 0.015 -25.590 0.000 0.270

WB6T2$4 0.577 0.015 38.678 0.000 0.198

WB7T2$1 -1.818 0.024 -76.525 0.000 0.303

WB7T2$2 -1.173 0.019 -60.922 0.000 0.297

WB7T2$3 -0.408 0.016 -24.944 0.000 0.257

WB7T2$4 0.502 0.017 30.317 0.000 0.248

WB1T3$1 -1.636 0.023 -71.276 0.000 0.234

WB1T3$2 -0.875 0.017 -51.958 0.000 0.196

WB1T3$3 0.014 0.015 0.967 0.334 0.230

WB1T3$4 0.965 0.018 53.746 0.000 0.263

WB2T3$1 -1.580 0.021 -74.849 0.000 0.226

WB2T3$2 -0.824 0.017 -49.789 0.000 0.191

WB2T3$3 0.050 0.015 3.303 0.001 0.207

WB2T3$4 1.048 0.019 53.977 0.000 0.286

WB3T3$1 -1.575 0.021 -76.287 0.000 0.271

WB3T3$2 -0.776 0.016 -48.537 0.000 0.241

WB3T3$3 0.021 0.015 1.441 0.150 0.224

WB3T3$4 0.943 0.017 53.906 0.000 0.266

WB4T3$1 -1.452 0.021 -70.212 0.000 0.274

WB4T3$2 -0.777 0.017 -46.135 0.000 0.238

WB4T3$3 -0.004 0.015 -0.242 0.808 0.195

WB4T3$4 0.904 0.018 49.397 0.000 0.280

WB5T3$1 -1.521 0.022 -69.456 0.000 0.277

WB5T3$2 -0.817 0.018 -44.686 0.000 0.264

WB5T3$3 -0.055 0.016 -3.436 0.001 0.216

WB5T3$4 0.800 0.019 43.084 0.000 0.259

WB6T3$1 -1.814 0.023 -79.089 0.000 0.342

WB6T3$2 -1.166 0.018 -64.173 0.000 0.287

WB6T3$3 -0.388 0.015 -26.425 0.000 0.251

WB6T3$4 0.585 0.016 36.337 0.000 0.246

WB7T3$1 -1.764 0.023 -77.222 0.000 0.265

WB7T3$2 -1.138 0.018 -62.541 0.000 0.248

WB7T3$3 -0.396 0.016 -25.373 0.000 0.235

WB7T3$4 0.487 0.016 29.572 0.000 0.283

SLT1$1 -0.374 0.021 -17.928 0.000 0.435

SLT2$1 -0.194 0.021 -9.368 0.000 0.551

SLT3$1 -0.086 0.019 -4.579 0.000 0.428

Variances

RI_PA 1.000 0.000 ********* 0.000 0.000

RI_SL 1.000 0.000 ********* 0.000 0.000

RI_WB 1.000 0.000 ********* 0.000 0.000

W_PAT1 1.000 0.000 ********* 0.000 0.000

W_PAT2 0.968 0.009 105.677 0.000 0.516

W_PAT3 0.956 0.011 86.229 0.000 0.467

W_SLT1 1.000 0.000 ********* 0.000 0.000

W_SLT2 0.799 0.053 14.999 0.000 0.633

W_SLT3 0.774 0.052 14.760 0.000 0.689

W_WBT1 1.000 0.000 ********* 0.000 0.000

W_WBT2 0.952 0.015 62.408 0.000 0.568

W_WBT3 0.929 0.015 62.965 0.000 0.553

Residual Variances

WB1T1 0.643 0.009 74.282 0.000 0.405

WB2T1 0.495 0.010 51.992 0.000 0.480

WB3T1 0.475 0.009 50.512 0.000 0.367

WB4T1 0.487 0.009 52.166 0.000 0.458

WB5T1 0.331 0.009 38.716 0.000 0.430

WB6T1 0.600 0.008 70.818 0.000 0.385

WB7T1 0.471 0.009 51.440 0.000 0.413

WB1T2 0.554 0.009 60.201 0.000 0.460

WB2T2 0.404 0.009 46.338 0.000 0.514

WB3T2 0.433 0.009 47.785 0.000 0.427

WB4T2 0.445 0.009 49.102 0.000 0.416

WB5T2 0.289 0.008 34.930 0.000 0.420

WB6T2 0.540 0.009 59.812 0.000 0.432

WB7T2 0.421 0.009 48.928 0.000 0.415

WB1T3 0.482 0.009 54.344 0.000 0.505

WB2T3 0.321 0.009 37.605 0.000 0.501

WB3T3 0.387 0.008 46.980 0.000 0.362

WB4T3 0.361 0.008 45.139 0.000 0.450

WB5T3 0.247 0.007 33.776 0.000 0.494

WB6T3 0.458 0.009 49.641 0.000 0.498

WB7T3 0.376 0.009 42.544 0.000 0.441

SLT1 0.000 999.000 999.000 999.000 0.000

SLT2 0.000 999.000 999.000 999.000 0.000

SLT3 0.000 999.000 999.000 999.000 0.000

PAT1 0.000 999.000 999.000 999.000 0.000

PAT2 0.000 999.000 999.000 999.000 0.000

PAT3 0.000 999.000 999.000 999.000 0.000

WBT1 0.000 999.000 999.000 999.000 0.000

WBT2 0.000 999.000 999.000 999.000 0.000

WBT3 0.000 999.000 999.000 999.000 0.000

Group FEMALE

WBT1 BY

WB1T1 0.604 0.008 75.117 0.000 0.364

WB2T1 0.708 0.007 104.155 0.000 0.386

WB3T1 0.739 0.006 132.240 0.000 0.439

WB4T1 0.723 0.007 108.821 0.000 0.390

WB5T1 0.842 0.004 191.368 0.000 0.486

WB6T1 0.634 0.007 87.633 0.000 0.378

WB7T1 0.724 0.006 115.851 0.000 0.438

WBT2 BY

WB1T2 0.655 0.008 83.302 0.000 0.461

WB2T2 0.753 0.006 118.933 0.000 0.407

WB3T2 0.755 0.006 128.750 0.000 0.455

WB4T2 0.745 0.006 123.821 0.000 0.443

WB5T2 0.859 0.004 195.527 0.000 0.510

WB6T2 0.661 0.007 100.694 0.000 0.394

WB7T2 0.736 0.007 111.007 0.000 0.470

WBT3 BY

WB1T3 0.704 0.007 105.696 0.000 0.491

WB2T3 0.797 0.005 149.993 0.000 0.498

WB3T3 0.774 0.006 138.203 0.000 0.516

WB4T3 0.790 0.005 144.844 0.000 0.526

WB5T3 0.872 0.004 215.215 0.000 0.550

WB6T3 0.697 0.006 109.233 0.000 0.456

WB7T3 0.759 0.006 127.149 0.000 0.446

RI_PA BY

PAT1 0.616 0.011 53.931 0.000 0.458

PAT2 0.634 0.014 45.253 0.000 0.400

PAT3 0.634 0.012 54.608 0.000 0.427

RI_SL BY

SLT1 0.665 0.018 37.645 0.000 0.552

SLT2 0.654 0.021 31.632 0.000 0.549

SLT3 0.654 0.021 31.566 0.000 0.544

W_PAT1 BY

PAT1 0.780 0.009 86.003 0.000 0.448

W_PAT2 BY

PAT2 0.766 0.012 65.584 0.000 0.390

W_PAT3 BY

PAT3 0.766 0.010 79.521 0.000 0.415

W_SLT1 BY

SLT1 0.740 0.016 47.121 0.000 0.554

W_SLT2 BY

SLT2 0.750 0.018 41.884 0.000 0.550

W_SLT3 BY

SLT3 0.750 0.018 41.859 0.000 0.546

RI_WB BY

WBT1 0.599 0.017 34.540 0.000 0.518

WBT2 0.594 0.018 33.225 0.000 0.491

WBT3 0.573 0.016 35.123 0.000 0.481

W_WBT1 BY

WBT1 0.791 0.013 60.474 0.000 0.522

W_WBT2 BY

WBT2 0.795 0.013 59.716 0.000 0.493

W_WBT3 BY

WBT3 0.811 0.011 70.542 0.000 0.485

W_PAT2 ON

W_PAT1 0.152 0.026 5.949 0.000 0.501

W_SLT1 0.016 0.038 0.419 0.676 0.595

W_WBT1 0.044 0.030 1.463 0.144 0.596

W_SLT2 ON

W_PAT1 -0.032 0.032 -0.990 0.322 0.593

W_SLT1 0.162 0.051 3.190 0.001 0.531

W_WBT1 0.112 0.041 2.718 0.007 0.578

W_WBT2 ON

W_PAT1 -0.001 0.024 -0.027 0.978 0.539

W_SLT1 0.080 0.037 2.188 0.029 0.560

W_WBT1 0.193 0.037 5.213 0.000 0.648

W_PAT3 ON

W_PAT2 0.174 0.028 6.105 0.000 0.538

W_SLT2 0.012 0.036 0.341 0.733 0.607

W_WBT2 0.010 0.030 0.350 0.727 0.608

W_SLT3 ON

W_PAT2 -0.048 0.028 -1.698 0.090 0.480

W_SLT2 0.226 0.044 5.158 0.000 0.489

W_WBT2 0.025 0.034 0.725 0.468 0.580

W_WBT3 ON

W_PAT2 -0.011 0.021 -0.527 0.598 0.534

W_SLT2 0.054 0.031 1.723 0.085 0.525

W_WBT2 0.221 0.027 8.090 0.000 0.526

WBT1 ON

ETH 0.087 0.007 12.676 0.000 0.227

FSM -0.063 0.007 -9.732 0.000 0.350

SEN -0.053 0.006 -9.411 0.000 0.222

WBT2 ON

ETH 0.086 0.007 12.569 0.000 0.225

FSM -0.063 0.006 -9.787 0.000 0.335

SEN -0.053 0.006 -9.468 0.000 0.225

WBT3 ON

ETH 0.083 0.007 12.602 0.000 0.227

FSM -0.061 0.006 -9.803 0.000 0.343

SEN -0.051 0.005 -9.455 0.000 0.227

PAT1 ON

ETH -0.088 0.007 -12.289 0.000 0.233

FSM -0.038 0.006 -6.224 0.000 0.205

SEN -0.040 0.005 -7.423 0.000 0.230

PAT2 ON

ETH -0.090 0.007 -12.237 0.000 0.228

FSM -0.039 0.006 -6.230 0.000 0.204

SEN -0.041 0.005 -7.439 0.000 0.228

PAT3 ON

ETH -0.090 0.007 -12.347 0.000 0.225

FSM -0.039 0.006 -6.202 0.000 0.206

SEN -0.041 0.005 -7.479 0.000 0.229

SLT1 ON

ETH 0.084 0.007 11.487 0.000 0.205

FSM -0.056 0.007 -8.061 0.000 0.306

SEN -0.013 0.006 -2.247 0.025 0.274

SLT2 ON

ETH 0.083 0.007 11.551 0.000 0.203

FSM -0.055 0.007 -8.079 0.000 0.305

SEN -0.013 0.006 -2.248 0.025 0.273

SLT3 ON

ETH 0.083 0.007 11.553 0.000 0.203

FSM -0.055 0.007 -8.082 0.000 0.305

SEN -0.013 0.006 -2.249 0.025 0.273

RI_PA WITH

RI_SL 0.169 0.037 4.594 0.000 0.520

RI_WB 0.271 0.035 7.746 0.000 0.533

RI_SL WITH

RI_WB 0.695 0.031 22.450 0.000 0.513

W_PAT1 WITH

W_WBT1 0.134 0.022 5.982 0.000 0.531

W_SLT1 0.099 0.029 3.431 0.001 0.497

W_WBT1 WITH

W_SLT1 0.460 0.027 16.877 0.000 0.469

W_PAT2 WITH

W_SLT2 0.020 0.028 0.723 0.470 0.492

W_WBT2 0.089 0.022 3.997 0.000 0.466

W_SLT2 WITH

W_WBT2 0.354 0.027 13.186 0.000 0.515

W_PAT3 WITH

W_SLT3 0.053 0.024 2.211 0.027 0.530

W_WBT3 0.090 0.018 4.976 0.000 0.448

W_SLT3 WITH

W_WBT3 0.292 0.022 13.458 0.000 0.503

WB1T1 WITH

WB1T2 0.262 0.017 14.959 0.000 0.622

WB1T3 0.193 0.023 8.392 0.000 0.681

WB1T2 WITH

WB1T3 0.318 0.021 15.293 0.000 0.653

WB2T1 WITH

WB2T2 0.148 0.019 7.885 0.000 0.565

WB2T3 0.123 0.023 5.252 0.000 0.599

WB2T2 WITH

WB2T3 0.166 0.025 6.746 0.000 0.673

WB3T1 WITH

WB3T2 0.217 0.024 9.228 0.000 0.672

WB3T3 0.162 0.022 7.477 0.000 0.559

WB3T2 WITH

WB3T3 0.234 0.022 10.663 0.000 0.570

WB4T1 WITH

WB4T2 0.141 0.022 6.499 0.000 0.605

WB4T3 0.079 0.026 3.067 0.002 0.661

WB4T2 WITH

WB4T3 0.106 0.024 4.371 0.000 0.652

WB5T1 WITH

WB5T2 0.183 0.027 6.801 0.000 0.516

WB5T3 0.186 0.033 5.693 0.000 0.589

WB5T2 WITH

WB5T3 0.205 0.033 6.170 0.000 0.634

WB6T1 WITH

WB6T2 0.202 0.019 10.698 0.000 0.573

WB6T3 0.160 0.021 7.785 0.000 0.637

WB6T2 WITH

WB6T3 0.222 0.020 10.928 0.000 0.630

WB7T1 WITH

WB7T2 0.203 0.021 9.441 0.000 0.592

WB7T3 0.136 0.025 5.513 0.000 0.617

WB7T2 WITH

WB7T3 0.204 0.023 8.881 0.000 0.642

Means

RI_PA 0.000 0.000 0.000 1.000 0.000

RI_SL 0.000 0.000 0.000 1.000 0.000

RI_WB 0.000 0.000 0.000 1.000 0.000

W_PAT1 0.000 0.000 0.000 1.000 0.000

W_PAT2 0.000 0.000 0.000 1.000 0.000

W_PAT3 0.000 0.000 0.000 1.000 0.000

W_SLT1 0.000 0.000 0.000 1.000 0.000

W_SLT2 0.000 0.000 0.000 1.000 0.000

W_SLT3 0.000 0.000 0.000 1.000 0.000

W_WBT1 0.000 0.000 0.000 1.000 0.000

W_WBT2 0.000 0.000 0.000 1.000 0.000

W_WBT3 0.000 0.000 0.000 1.000 0.000

Intercepts

PAT1 1.741 0.025 69.997 0.000 0.159

PAT2 1.765 0.027 64.654 0.000 0.249

PAT3 1.709 0.025 69.474 0.000 0.227

WBT1 -0.406 0.026 -15.381 0.000 0.145

WBT2 -0.498 0.029 -17.030 0.000 0.213

WBT3 -0.432 0.027 -16.113 0.000 0.242

Thresholds

WB1T1$1 -1.550 0.022 -70.039 0.000 0.300

WB1T1$2 -0.829 0.016 -50.542 0.000 0.247

WB1T1$3 0.013 0.014 0.967 0.333 0.229

WB1T1$4 0.914 0.017 53.639 0.000 0.269

WB2T1$1 -1.531 0.021 -71.448 0.000 0.329

WB2T1$2 -0.798 0.016 -48.607 0.000 0.232

WB2T1$3 0.049 0.015 3.310 0.001 0.206

WB2T1$4 1.016 0.018 55.882 0.000 0.286

WB3T1$1 -1.677 0.021 -79.985 0.000 0.241

WB3T1$2 -0.826 0.017 -48.929 0.000 0.226

WB3T1$3 0.023 0.016 1.442 0.149 0.224

WB3T1$4 1.004 0.018 55.747 0.000 0.242

WB4T1$1 -1.482 0.022 -68.608 0.000 0.280

WB4T1$2 -0.793 0.018 -44.746 0.000 0.263

WB4T1$3 -0.004 0.015 -0.243 0.808 0.195

WB4T1$4 0.923 0.017 52.855 0.000 0.234

WB5T1$1 -1.664 0.023 -71.961 0.000 0.280

WB5T1$2 -0.894 0.020 -45.454 0.000 0.253

WB5T1$3 -0.060 0.017 -3.432 0.001 0.215

WB5T1$4 0.875 0.019 45.422 0.000 0.245

WB6T1$1 -1.764 0.024 -72.832 0.000 0.423

WB6T1$2 -1.133 0.018 -61.401 0.000 0.347

WB6T1$3 -0.378 0.015 -25.584 0.000 0.282

WB6T1$4 0.569 0.015 37.721 0.000 0.217

WB7T1$1 -1.824 0.023 -77.756 0.000 0.303

WB7T1$2 -1.177 0.019 -62.458 0.000 0.261

WB7T1$3 -0.409 0.016 -25.046 0.000 0.245

WB7T1$4 0.504 0.017 30.418 0.000 0.262

WB1T2$1 -1.666 0.022 -76.478 0.000 0.296

WB1T2$2 -0.891 0.017 -52.953 0.000 0.236

WB1T2$3 0.014 0.015 0.967 0.333 0.229

WB1T2$4 0.982 0.019 52.665 0.000 0.304

WB2T2$1 -1.615 0.022 -74.178 0.000 0.273

WB2T2$2 -0.842 0.017 -48.625 0.000 0.218

WB2T2$3 0.051 0.015 3.311 0.001 0.206

WB2T2$4 1.072 0.019 56.447 0.000 0.276

WB3T2$1 -1.697 0.022 -76.953 0.000 0.313

WB3T2$2 -0.836 0.018 -47.373 0.000 0.260

WB3T2$3 0.023 0.016 1.442 0.149 0.223

WB3T2$4 1.016 0.018 57.116 0.000 0.225

WB4T2$1 -1.513 0.021 -70.607 0.000 0.296

WB4T2$2 -0.809 0.018 -45.698 0.000 0.254

WB4T2$3 -0.004 0.015 -0.243 0.808 0.195

WB4T2$4 0.942 0.018 52.603 0.000 0.259

WB5T2$1 -1.684 0.023 -72.459 0.000 0.258

WB5T2$2 -0.904 0.020 -44.796 0.000 0.262

WB5T2$3 -0.061 0.018 -3.429 0.001 0.216

WB5T2$4 0.886 0.019 45.700 0.000 0.241

WB6T2$1 -1.822 0.023 -80.518 0.000 0.314

WB6T2$2 -1.171 0.018 -63.766 0.000 0.308

WB6T2$3 -0.390 0.015 -25.938 0.000 0.261

WB6T2$4 0.588 0.016 37.211 0.000 0.239

WB7T2$1 -1.838 0.023 -80.588 0.000 0.311

WB7T2$2 -1.185 0.019 -61.630 0.000 0.310

WB7T2$3 -0.412 0.017 -24.806 0.000 0.267

WB7T2$4 0.508 0.016 30.853 0.000 0.247

WB1T3$1 -1.728 0.023 -74.782 0.000 0.332

WB1T3$2 -0.924 0.018 -51.606 0.000 0.287

WB1T3$3 0.015 0.015 0.967 0.333 0.228

WB1T3$4 1.019 0.018 56.598 0.000 0.256

WB2T3$1 -1.651 0.024 -69.910 0.000 0.341

WB2T3$2 -0.861 0.018 -47.687 0.000 0.234

WB2T3$3 0.052 0.016 3.311 0.001 0.207

WB2T3$4 1.095 0.019 56.276 0.000 0.304

WB3T3$1 -1.680 0.022 -76.472 0.000 0.340

WB3T3$2 -0.828 0.017 -48.001 0.000 0.286

WB3T3$3 0.023 0.016 1.442 0.149 0.222

WB3T3$4 1.006 0.018 56.758 0.000 0.224

WB4T3$1 -1.549 0.022 -71.608 0.000 0.286

WB4T3$2 -0.828 0.018 -46.129 0.000 0.248

WB4T3$3 -0.004 0.016 -0.243 0.808 0.195

WB4T3$4 0.964 0.019 51.913 0.000 0.284

WB5T3$1 -1.650 0.023 -70.894 0.000 0.309

WB5T3$2 -0.886 0.020 -44.839 0.000 0.287

WB5T3$3 -0.059 0.017 -3.432 0.001 0.216

WB5T3$4 0.867 0.019 45.058 0.000 0.266

WB6T3$1 -1.853 0.022 -82.750 0.000 0.310

WB6T3$2 -1.191 0.018 -66.049 0.000 0.292

WB6T3$3 -0.397 0.015 -26.405 0.000 0.248

WB6T3$4 0.598 0.016 37.230 0.000 0.245

WB7T3$1 -1.831 0.022 -82.120 0.000 0.276

WB7T3$2 -1.181 0.019 -62.891 0.000 0.287

WB7T3$3 -0.411 0.016 -25.279 0.000 0.247

WB7T3$4 0.505 0.017 30.357 0.000 0.273

SLT1$1 -0.316 0.017 -18.338 0.000 0.372

SLT2$1 -0.176 0.019 -9.494 0.000 0.538

SLT3$1 -0.079 0.017 -4.599 0.000 0.422

Variances

RI_PA 1.000 0.000 ********* 0.000 0.000

RI_SL 1.000 0.000 ********* 0.000 0.000

RI_WB 1.000 0.000 ********* 0.000 0.000

W_PAT1 1.000 0.000 ********* 0.000 0.000

W_PAT2 0.970 0.009 109.122 0.000 0.481

W_PAT3 0.967 0.010 92.766 0.000 0.522

W_SLT1 1.000 0.000 ********* 0.000 0.000

W_SLT2 0.943 0.020 48.217 0.000 0.540

W_SLT3 0.941 0.020 47.580 0.000 0.514

W_WBT1 1.000 0.000 ********* 0.000 0.000

W_WBT2 0.940 0.015 62.832 0.000 0.574

W_WBT3 0.938 0.013 70.784 0.000 0.503

Residual Variances

WB1T1 0.635 0.010 65.407 0.000 0.363

WB2T1 0.499 0.010 51.954 0.000 0.386

WB3T1 0.453 0.008 54.846 0.000 0.439

WB4T1 0.477 0.010 49.647 0.000 0.390

WB5T1 0.292 0.007 39.401 0.000 0.486

WB6T1 0.597 0.009 65.030 0.000 0.378

WB7T1 0.475 0.009 52.531 0.000 0.438

WB1T2 0.570 0.010 55.337 0.000 0.461

WB2T2 0.433 0.010 45.361 0.000 0.407

WB3T2 0.430 0.009 48.482 0.000 0.455

WB4T2 0.445 0.009 49.696 0.000 0.443

WB5T2 0.261 0.008 34.575 0.000 0.510

WB6T2 0.563 0.009 64.741 0.000 0.394

WB7T2 0.458 0.010 46.832 0.000 0.471

WB1T3 0.505 0.009 53.859 0.000 0.491

WB2T3 0.364 0.008 43.006 0.000 0.498

WB3T3 0.401 0.009 46.268 0.000 0.516

WB4T3 0.376 0.009 43.676 0.000 0.527

WB5T3 0.240 0.007 33.947 0.000 0.550

WB6T3 0.514 0.009 57.857 0.000 0.456

WB7T3 0.423 0.009 46.658 0.000 0.446

SLT1 0.000 999.000 999.000 999.000 0.000

SLT2 0.000 999.000 999.000 999.000 0.000

SLT3 0.000 999.000 999.000 999.000 0.000

PAT1 0.000 999.000 999.000 999.000 0.000

PAT2 0.000 999.000 999.000 999.000 0.000

PAT3 0.000 999.000 999.000 999.000 0.000

WBT1 0.000 999.000 999.000 999.000 0.000

WBT2 0.000 999.000 999.000 999.000 0.000

WBT3 0.000 999.000 999.000 999.000 0.000

UNSTANDARDIZED MODEL RESULTS

Two-Tailed Rate of

Estimate S.E. Est./S.E. P-Value Missing

Group MALE

WBT1 BY

WB1T1 0.709 0.018 40.433 0.000 0.432

WB2T1 0.960 0.021 44.726 0.000 0.468

WB3T1 1.000 0.000 999.000 999.000 0.000

WB4T1 0.977 0.025 39.182 0.000 0.484

WB5T1 1.352 0.031 43.431 0.000 0.462

WB6T1 0.776 0.018 43.464 0.000 0.406

WB7T1 1.008 0.023 43.015 0.000 0.435

WBT2 BY

WB1T2 0.709 0.018 40.433 0.000 0.432

WB2T2 0.960 0.021 44.726 0.000 0.468

WB3T2 1.000 0.000 999.000 999.000 0.000

WB4T2 0.977 0.025 39.182 0.000 0.484

WB5T2 1.352 0.031 43.431 0.000 0.462

WB6T2 0.776 0.018 43.464 0.000 0.406

WB7T2 1.008 0.023 43.015 0.000 0.435

WBT3 BY

WB1T3 0.709 0.018 40.433 0.000 0.432

WB2T3 0.960 0.021 44.726 0.000 0.468

WB3T3 1.000 0.000 999.000 999.000 0.000

WB4T3 0.977 0.025 39.182 0.000 0.484

WB5T3 1.352 0.031 43.431 0.000 0.462

WB6T3 0.776 0.018 43.464 0.000 0.406

WB7T3 1.008 0.023 43.015 0.000 0.435

RI_PA BY

PAT1 1.000 0.000 999.000 999.000 0.000

PAT2 1.000 0.000 999.000 999.000 0.000

PAT3 1.000 0.000 999.000 999.000 0.000

RI_SL BY

SLT1 1.000 0.000 999.000 999.000 0.000

SLT2 1.000 0.000 999.000 999.000 0.000

SLT3 1.000 0.000 999.000 999.000 0.000

W_PAT1 BY

PAT1 1.000 0.000 999.000 999.000 0.000

W_PAT2 BY

PAT2 1.000 0.000 999.000 999.000 0.000

W_PAT3 BY

PAT3 1.000 0.000 999.000 999.000 0.000

W_SLT1 BY

SLT1 1.000 0.000 999.000 999.000 0.000

W_SLT2 BY

SLT2 1.000 0.000 999.000 999.000 0.000

W_SLT3 BY

SLT3 1.000 0.000 999.000 999.000 0.000

RI_WB BY

WBT1 1.000 0.000 999.000 999.000 0.000

WBT2 1.000 0.000 999.000 999.000 0.000

WBT3 1.000 0.000 999.000 999.000 0.000

W_WBT1 BY

WBT1 1.000 0.000 999.000 999.000 0.000

W_WBT2 BY

WBT2 1.000 0.000 999.000 999.000 0.000

W_WBT3 BY

WBT3 1.000 0.000 999.000 999.000 0.000

W_PAT2 ON

W_PAT1 0.156 0.025 6.287 0.000 0.506

W_SLT1 0.018 0.073 0.247 0.805 0.708

W_WBT1 0.094 0.058 1.622 0.105 0.598

W_SLT2 ON

W_PAT1 -0.020 0.019 -1.031 0.303 0.631

W_SLT1 0.502 0.086 5.823 0.000 0.641

W_WBT1 -0.015 0.046 -0.328 0.743 0.544

W_WBT2 ON

W_PAT1 0.007 0.012 0.554 0.580 0.620

W_SLT1 0.058 0.033 1.731 0.083 0.596

W_WBT1 0.193 0.036 5.435 0.000 0.542

W_PAT3 ON

W_PAT2 0.191 0.026 7.360 0.000 0.510

W_SLT2 0.012 0.064 0.181 0.856 0.700

W_WBT2 0.081 0.052 1.544 0.123 0.585

W_SLT3 ON

W_PAT2 -0.020 0.018 -1.087 0.277 0.694

W_SLT2 0.484 0.062 7.809 0.000 0.697

W_WBT2 -0.026 0.039 -0.661 0.508 0.644

W_WBT3 ON

W_PAT2 0.001 0.011 0.067 0.947 0.588

W_SLT2 0.073 0.028 2.562 0.010 0.513

W_WBT2 0.251 0.028 8.951 0.000 0.504

WBT1 ON

ETH 0.189 0.015 12.233 0.000 0.252

FSM -0.146 0.015 -9.795 0.000 0.340

SEN -0.169 0.018 -9.441 0.000 0.213

WBT2 ON

ETH 0.189 0.015 12.233 0.000 0.252

FSM -0.146 0.015 -9.795 0.000 0.340

SEN -0.169 0.018 -9.441 0.000 0.213

WBT3 ON

ETH 0.189 0.015 12.233 0.000 0.252

FSM -0.146 0.015 -9.795 0.000 0.340

SEN -0.169 0.018 -9.441 0.000 0.213

PAT1 ON

ETH -0.400 0.033 -12.296 0.000 0.229

FSM -0.184 0.030 -6.239 0.000 0.206

SEN -0.266 0.036 -7.452 0.000 0.229

PAT2 ON

ETH -0.400 0.033 -12.296 0.000 0.229

FSM -0.184 0.030 -6.239 0.000 0.206

SEN -0.266 0.036 -7.452 0.000 0.229

PAT3 ON

ETH -0.400 0.033 -12.296 0.000 0.229

FSM -0.184 0.030 -6.239 0.000 0.206

SEN -0.266 0.036 -7.452 0.000 0.229

SLT1 ON

ETH 0.237 0.021 11.427 0.000 0.211

FSM -0.166 0.021 -7.944 0.000 0.320

SEN -0.054 0.024 -2.245 0.025 0.273

SLT2 ON

ETH 0.237 0.021 11.427 0.000 0.211

FSM -0.166 0.021 -7.944 0.000 0.320

SEN -0.054 0.024 -2.245 0.025 0.273

SLT3 ON

ETH 0.237 0.021 11.427 0.000 0.211

FSM -0.166 0.021 -7.944 0.000 0.320

SEN -0.054 0.024 -2.245 0.025 0.273

RI_PA WITH

RI_SL 0.202 0.071 2.858 0.004 0.704

RI_WB 0.290 0.037 7.809 0.000 0.603

RI_SL WITH

RI_WB 0.301 0.041 7.335 0.000 0.556

W_PAT1 WITH

W_WBT1 0.187 0.040 4.703 0.000 0.559

W_SLT1 0.097 0.071 1.360 0.174 0.656

W_WBT1 WITH

W_SLT1 0.264 0.035 7.595 0.000 0.576

W_PAT2 WITH

W_SLT2 0.035 0.047 0.739 0.460 0.517

W_WBT2 0.189 0.035 5.376 0.000 0.564

W_SLT2 WITH

W_WBT2 0.229 0.024 9.464 0.000 0.464

W_PAT3 WITH

W_SLT3 0.095 0.037 2.608 0.009 0.476

W_WBT3 0.215 0.028 7.795 0.000 0.476

W_SLT3 WITH

W_WBT3 0.250 0.021 11.743 0.000 0.575

WB1T1 WITH

WB1T2 0.230 0.017 13.449 0.000 0.643

WB1T3 0.158 0.017 9.235 0.000 0.648

WB1T2 WITH

WB1T3 0.233 0.016 14.783 0.000 0.594

WB2T1 WITH

WB2T2 0.136 0.017 7.944 0.000 0.553

WB2T3 0.083 0.020 4.250 0.000 0.620

WB2T2 WITH

WB2T3 0.126 0.015 8.169 0.000 0.532

WB3T1 WITH

WB3T2 0.193 0.020 9.533 0.000 0.550

WB3T3 0.159 0.023 6.762 0.000 0.616

WB3T2 WITH

WB3T3 0.203 0.020 10.150 0.000 0.571

WB4T1 WITH

WB4T2 0.123 0.022 5.690 0.000 0.609

WB4T3 0.081 0.021 3.793 0.000 0.641

WB4T2 WITH

WB4T3 0.081 0.020 3.957 0.000 0.658

WB5T1 WITH

WB5T2 0.130 0.025 5.155 0.000 0.559

WB5T3 0.164 0.030 5.561 0.000 0.570

WB5T2 WITH

WB5T3 0.161 0.028 5.723 0.000 0.650

WB6T1 WITH

WB6T2 0.197 0.019 10.632 0.000 0.591

WB6T3 0.144 0.019 7.418 0.000 0.634

WB6T2 WITH

WB6T3 0.177 0.018 9.943 0.000 0.612

WB7T1 WITH

WB7T2 0.180 0.022 8.247 0.000 0.545

WB7T3 0.097 0.022 4.333 0.000 0.547

WB7T2 WITH

WB7T3 0.154 0.022 6.992 0.000 0.631

Means

RI_PA 0.000 0.000 999.000 999.000 0.000

RI_SL 0.000 0.000 999.000 999.000 0.000

RI_WB 0.000 0.000 999.000 999.000 0.000

W_PAT1 0.000 0.000 999.000 999.000 0.000

W_PAT2 0.000 0.000 999.000 999.000 0.000

W_PAT3 0.000 0.000 999.000 999.000 0.000

W_SLT1 0.000 0.000 999.000 999.000 0.000

W_SLT2 0.000 0.000 999.000 999.000 0.000

W_SLT3 0.000 0.000 999.000 999.000 0.000

W_WBT1 0.000 0.000 999.000 999.000 0.000

W_WBT2 0.000 0.000 999.000 999.000 0.000

W_WBT3 0.000 0.000 999.000 999.000 0.000

Intercepts

PAT1 3.805 0.034 111.719 0.000 0.325

PAT2 3.754 0.035 106.786 0.000 0.464

PAT3 3.634 0.034 106.610 0.000 0.422

WBT1 0.000 0.000 999.000 999.000 0.000

WBT2 -0.062 0.021 -3.017 0.003 0.340

WBT3 -0.060 0.024 -2.483 0.013 0.340

Thresholds

WB1T1$1 -1.888 0.036 -53.084 0.000 0.320

WB1T1$2 -1.009 0.024 -42.359 0.000 0.272

WB1T1$3 0.016 0.017 0.968 0.333 0.229

WB1T1$4 1.113 0.024 46.449 0.000 0.329

WB2T1$1 -2.157 0.040 -53.269 0.000 0.399

WB2T1$2 -1.125 0.027 -41.211 0.000 0.281

WB2T1$3 0.068 0.021 3.319 0.001 0.207

WB2T1$4 1.431 0.030 48.419 0.000 0.387

WB3T1$1 -2.355 0.039 -60.882 0.000 0.314

WB3T1$2 -1.160 0.026 -43.860 0.000 0.245

WB3T1$3 0.032 0.022 1.442 0.149 0.225

WB3T1$4 1.410 0.029 48.053 0.000 0.330

WB4T1$1 -2.079 0.042 -49.009 0.000 0.356

WB4T1$2 -1.112 0.029 -38.240 0.000 0.288

WB4T1$3 -0.005 0.021 -0.242 0.809 0.195

WB4T1$4 1.294 0.031 41.779 0.000 0.330

WB5T1$1 -2.776 0.053 -52.754 0.000 0.458

WB5T1$2 -1.491 0.037 -40.553 0.000 0.358

WB5T1$3 -0.100 0.029 -3.439 0.001 0.219

WB5T1$4 1.460 0.038 38.557 0.000 0.305

WB6T1$1 -2.240 0.039 -57.624 0.000 0.352

WB6T1$2 -1.440 0.029 -50.004 0.000 0.301

WB6T1$3 -0.480 0.020 -24.115 0.000 0.255

WB6T1$4 0.723 0.020 36.686 0.000 0.274

WB7T1$1 -2.637 0.046 -57.136 0.000 0.406

WB7T1$2 -1.701 0.034 -50.299 0.000 0.384

WB7T1$3 -0.591 0.025 -23.806 0.000 0.299

WB7T1$4 0.728 0.025 29.123 0.000 0.242

WB1T2$1 -1.888 0.036 -53.084 0.000 0.320

WB1T2$2 -1.009 0.024 -42.359 0.000 0.272

WB1T2$3 0.016 0.017 0.968 0.333 0.229

WB1T2$4 1.113 0.024 46.449 0.000 0.329

WB2T2$1 -2.157 0.040 -53.269 0.000 0.399

WB2T2$2 -1.125 0.027 -41.211 0.000 0.281

WB2T2$3 0.068 0.021 3.319 0.001 0.207

WB2T2$4 1.431 0.030 48.419 0.000 0.387

WB3T2$1 -2.355 0.039 -60.882 0.000 0.314

WB3T2$2 -1.160 0.026 -43.860 0.000 0.245

WB3T2$3 0.032 0.022 1.442 0.149 0.225

WB3T2$4 1.410 0.029 48.053 0.000 0.330

WB4T2$1 -2.079 0.042 -49.009 0.000 0.356

WB4T2$2 -1.112 0.029 -38.240 0.000 0.288

WB4T2$3 -0.005 0.021 -0.242 0.809 0.195

WB4T2$4 1.294 0.031 41.779 0.000 0.330

WB5T2$1 -2.776 0.053 -52.754 0.000 0.458

WB5T2$2 -1.491 0.037 -40.553 0.000 0.358

WB5T2$3 -0.100 0.029 -3.439 0.001 0.219

WB5T2$4 1.460 0.038 38.557 0.000 0.305

WB6T2$1 -2.240 0.039 -57.624 0.000 0.352

WB6T2$2 -1.440 0.029 -50.004 0.000 0.301

WB6T2$3 -0.480 0.020 -24.115 0.000 0.255

WB6T2$4 0.723 0.020 36.686 0.000 0.274

WB7T2$1 -2.637 0.046 -57.136 0.000 0.406

WB7T2$2 -1.701 0.034 -50.299 0.000 0.384

WB7T2$3 -0.591 0.025 -23.806 0.000 0.299

WB7T2$4 0.728 0.025 29.123 0.000 0.242

WB1T3$1 -1.888 0.036 -53.084 0.000 0.320

WB1T3$2 -1.009 0.024 -42.359 0.000 0.272

WB1T3$3 0.016 0.017 0.968 0.333 0.229

WB1T3$4 1.113 0.024 46.449 0.000 0.329

WB2T3$1 -2.157 0.040 -53.269 0.000 0.399

WB2T3$2 -1.125 0.027 -41.211 0.000 0.281

WB2T3$3 0.068 0.021 3.319 0.001 0.207

WB2T3$4 1.431 0.030 48.419 0.000 0.387

WB3T3$1 -2.355 0.039 -60.882 0.000 0.314

WB3T3$2 -1.160 0.026 -43.860 0.000 0.245

WB3T3$3 0.032 0.022 1.442 0.149 0.225

WB3T3$4 1.410 0.029 48.053 0.000 0.330

WB4T3$1 -2.079 0.042 -49.009 0.000 0.356

WB4T3$2 -1.112 0.029 -38.240 0.000 0.288

WB4T3$3 -0.005 0.021 -0.242 0.809 0.195

WB4T3$4 1.294 0.031 41.779 0.000 0.330

WB5T3$1 -2.776 0.053 -52.754 0.000 0.458

WB5T3$2 -1.491 0.037 -40.553 0.000 0.358

WB5T3$3 -0.100 0.029 -3.439 0.001 0.219

WB5T3$4 1.460 0.038 38.557 0.000 0.305

WB6T3$1 -2.240 0.039 -57.624 0.000 0.352

WB6T3$2 -1.440 0.029 -50.004 0.000 0.301

WB6T3$3 -0.480 0.020 -24.115 0.000 0.255

WB6T3$4 0.723 0.020 36.686 0.000 0.274

WB7T3$1 -2.637 0.046 -57.136 0.000 0.406

WB7T3$2 -1.701 0.034 -50.299 0.000 0.384

WB7T3$3 -0.591 0.025 -23.806 0.000 0.299

WB7T3$4 0.728 0.025 29.123 0.000 0.242

SLT1$1 -0.426 0.024 -17.999 0.000 0.380

SLT2$1 -0.242 0.026 -9.427 0.000 0.541

SLT3$1 -0.108 0.024 -4.559 0.000 0.430

Variances

RI_PA 2.084 0.087 24.040 0.000 0.317

RI_SL 0.285 0.113 2.516 0.012 0.640

RI_WB 0.409 0.029 14.155 0.000 0.521

W_PAT1 3.368 0.103 32.633 0.000 0.215

W_PAT2 3.062 0.100 30.606 0.000 0.160

W_PAT3 2.948 0.084 35.255 0.000 0.216

W_SLT1 1.000 0.000 999.000 999.000 0.000

W_SLT2 1.000 0.000 999.000 999.000 0.000

W_SLT3 1.000 0.000 999.000 999.000 0.000

W_WBT1 0.679 0.036 18.925 0.000 0.452

W_WBT2 0.734 0.035 21.006 0.000 0.415

W_WBT3 0.878 0.038 23.274 0.000 0.397

Residual Variances

WB1T1 1.000 0.000 999.000 999.000 0.000

WB2T1 1.000 0.000 999.000 999.000 0.000

WB3T1 1.000 0.000 999.000 999.000 0.000

WB4T1 1.000 0.000 999.000 999.000 0.000

WB5T1 1.000 0.000 999.000 999.000 0.000

WB6T1 1.000 0.000 999.000 999.000 0.000

WB7T1 1.000 0.000 999.000 999.000 0.000

WB1T2 0.749 0.031 24.406 0.000 0.472

WB2T2 0.748 0.033 22.860 0.000 0.503

WB3T2 0.916 0.035 26.162 0.000 0.478

WB4T2 0.915 0.044 20.666 0.000 0.473

WB5T2 0.889 0.041 21.854 0.000 0.509

WB6T2 0.849 0.035 24.420 0.000 0.439

WB7T2 0.886 0.037 24.227 0.000 0.414

WB1T3 0.642 0.027 23.755 0.000 0.484

WB2T3 0.599 0.028 21.209 0.000 0.559

WB3T3 0.865 0.035 24.841 0.000 0.450

WB4T3 0.739 0.032 23.139 0.000 0.511

WB5T3 0.822 0.038 21.864 0.000 0.494

WB6T3 0.699 0.030 23.433 0.000 0.534

WB7T3 0.840 0.035 23.879 0.000 0.437

SLT1 0.000 0.000 999.000 999.000 0.000

SLT2 0.000 0.000 999.000 999.000 0.000

SLT3 0.000 0.000 999.000 999.000 0.000

PAT1 0.000 0.000 999.000 999.000 0.000

PAT2 0.000 0.000 999.000 999.000 0.000

PAT3 0.000 0.000 999.000 999.000 0.000

WBT1 0.000 0.000 999.000 999.000 0.000

WBT2 0.000 0.000 999.000 999.000 0.000

WBT3 0.000 0.000 999.000 999.000 0.000

Group FEMALE

WBT1 BY

WB1T1 0.709 0.018 40.433 0.000 0.432

WB2T1 0.960 0.021 44.726 0.000 0.468

WB3T1 1.000 0.000 999.000 999.000 0.000

WB4T1 0.977 0.025 39.182 0.000 0.484

WB5T1 1.352 0.031 43.431 0.000 0.462

WB6T1 0.776 0.018 43.464 0.000 0.406

WB7T1 1.008 0.023 43.015 0.000 0.435

WBT2 BY

WB1T2 0.709 0.018 40.433 0.000 0.432

WB2T2 0.960 0.021 44.726 0.000 0.468

WB3T2 1.000 0.000 999.000 999.000 0.000

WB4T2 0.977 0.025 39.182 0.000 0.484

WB5T2 1.352 0.031 43.431 0.000 0.462

WB6T2 0.776 0.018 43.464 0.000 0.406

WB7T2 1.008 0.023 43.015 0.000 0.435

WBT3 BY

WB1T3 0.709 0.018 40.433 0.000 0.432

WB2T3 0.960 0.021 44.726 0.000 0.468

WB3T3 1.000 0.000 999.000 999.000 0.000

WB4T3 0.977 0.025 39.182 0.000 0.484

WB5T3 1.352 0.031 43.431 0.000 0.462

WB6T3 0.776 0.018 43.464 0.000 0.406

WB7T3 1.008 0.023 43.015 0.000 0.435

RI_PA BY

PAT1 1.000 0.000 999.000 999.000 0.000

PAT2 1.000 0.000 999.000 999.000 0.000

PAT3 1.000 0.000 999.000 999.000 0.000

RI_SL BY

SLT1 1.000 0.000 999.000 999.000 0.000

SLT2 1.000 0.000 999.000 999.000 0.000

SLT3 1.000 0.000 999.000 999.000 0.000

W_PAT1 BY

PAT1 1.000 0.000 999.000 999.000 0.000

W_PAT2 BY

PAT2 1.000 0.000 999.000 999.000 0.000

W_PAT3 BY

PAT3 1.000 0.000 999.000 999.000 0.000

W_SLT1 BY

SLT1 1.000 0.000 999.000 999.000 0.000

W_SLT2 BY

SLT2 1.000 0.000 999.000 999.000 0.000

W_SLT3 BY

SLT3 1.000 0.000 999.000 999.000 0.000

RI_WB BY

WBT1 1.000 0.000 999.000 999.000 0.000

WBT2 1.000 0.000 999.000 999.000 0.000

WBT3 1.000 0.000 999.000 999.000 0.000

W_WBT1 BY

WBT1 1.000 0.000 999.000 999.000 0.000

W_WBT2 BY

WBT2 1.000 0.000 999.000 999.000 0.000

W_WBT3 BY

WBT3 1.000 0.000 999.000 999.000 0.000

W_PAT2 ON

W_PAT1 0.145 0.026 5.682 0.000 0.480

W_SLT1 0.026 0.062 0.418 0.676 0.595

W_WBT1 0.087 0.060 1.463 0.143 0.594

W_SLT2 ON

W_PAT1 -0.019 0.019 -0.990 0.322 0.593

W_SLT1 0.167 0.054 3.114 0.002 0.531

W_WBT1 0.140 0.051 2.741 0.006 0.573

W_WBT2 ON

W_PAT1 0.000 0.012 -0.026 0.980 0.538

W_SLT1 0.067 0.031 2.184 0.029 0.553

W_WBT1 0.196 0.038 5.195 0.000 0.640

W_PAT3 ON

W_PAT2 0.174 0.028 6.280 0.000 0.546

W_SLT2 0.020 0.058 0.342 0.733 0.605

W_WBT2 0.020 0.058 0.348 0.728 0.612

W_SLT3 ON

W_PAT2 -0.030 0.018 -1.691 0.091 0.478

W_SLT2 0.226 0.045 5.036 0.000 0.494

W_WBT2 0.031 0.042 0.727 0.467 0.579

W_WBT3 ON

W_PAT2 -0.006 0.012 -0.526 0.599 0.534

W_SLT2 0.046 0.027 1.723 0.085 0.525

W_WBT2 0.233 0.028 8.211 0.000 0.528

WBT1 ON

ETH 0.189 0.015 12.233 0.000 0.252

FSM -0.146 0.015 -9.795 0.000 0.340

SEN -0.169 0.018 -9.441 0.000 0.213

WBT2 ON

ETH 0.189 0.015 12.233 0.000 0.252

FSM -0.146 0.015 -9.795 0.000 0.340

SEN -0.169 0.018 -9.441 0.000 0.213

WBT3 ON

ETH 0.189 0.015 12.233 0.000 0.252

FSM -0.146 0.015 -9.795 0.000 0.340

SEN -0.169 0.018 -9.441 0.000 0.213

PAT1 ON

ETH -0.400 0.033 -12.296 0.000 0.229

FSM -0.184 0.030 -6.239 0.000 0.206

SEN -0.266 0.036 -7.452 0.000 0.229

PAT2 ON

ETH -0.400 0.033 -12.296 0.000 0.229

FSM -0.184 0.030 -6.239 0.000 0.206

SEN -0.266 0.036 -7.452 0.000 0.229

PAT3 ON

ETH -0.400 0.033 -12.296 0.000 0.229

FSM -0.184 0.030 -6.239 0.000 0.206

SEN -0.266 0.036 -7.452 0.000 0.229

SLT1 ON

ETH 0.237 0.021 11.427 0.000 0.211

FSM -0.166 0.021 -7.944 0.000 0.320

SEN -0.054 0.024 -2.245 0.025 0.273

SLT2 ON

ETH 0.237 0.021 11.427 0.000 0.211

FSM -0.166 0.021 -7.944 0.000 0.320

SEN -0.054 0.024 -2.245 0.025 0.273

SLT3 ON

ETH 0.237 0.021 11.427 0.000 0.211

FSM -0.166 0.021 -7.944 0.000 0.320

SEN -0.054 0.024 -2.245 0.025 0.273

RI_PA WITH

RI_SL 0.204 0.046 4.463 0.000 0.513

RI_WB 0.228 0.032 7.200 0.000 0.522

RI_SL WITH

RI_WB 0.389 0.032 12.207 0.000 0.532

W_PAT1 WITH

W_WBT1 0.188 0.033 5.642 0.000 0.549

W_SLT1 0.169 0.049 3.418 0.001 0.497

W_WBT1 WITH

W_SLT1 0.378 0.026 14.317 0.000 0.489

W_PAT2 WITH

W_SLT2 0.033 0.045 0.724 0.469 0.492

W_WBT2 0.116 0.030 3.898 0.000 0.465

W_SLT2 WITH

W_WBT2 0.286 0.024 12.058 0.000 0.522

W_PAT3 WITH

W_SLT3 0.085 0.039 2.206 0.027 0.529

W_WBT3 0.123 0.025 4.845 0.000 0.447

W_SLT3 WITH

W_WBT3 0.248 0.020 12.506 0.000 0.502

WB1T1 WITH

WB1T2 0.218 0.017 13.061 0.000 0.527

WB1T3 0.145 0.017 8.318 0.000 0.640

WB1T2 WITH

WB1T3 0.212 0.015 13.875 0.000 0.546

WB2T1 WITH

WB2T2 0.130 0.017 7.786 0.000 0.562

WB2T3 0.096 0.019 5.152 0.000 0.620

WB2T2 WITH

WB2T3 0.115 0.018 6.486 0.000 0.687

WB3T1 WITH

WB3T2 0.187 0.021 9.074 0.000 0.647

WB3T3 0.136 0.018 7.365 0.000 0.561

WB3T2 WITH

WB3T3 0.189 0.018 10.558 0.000 0.542

WB4T1 WITH

WB4T2 0.125 0.020 6.391 0.000 0.607

WB4T3 0.063 0.021 3.079 0.002 0.663

WB4T2 WITH

WB4T3 0.080 0.018 4.343 0.000 0.654

WB5T1 WITH

WB5T2 0.139 0.021 6.680 0.000 0.529

WB5T3 0.138 0.024 5.825 0.000 0.571

WB5T2 WITH

WB5T3 0.142 0.023 6.111 0.000 0.629

WB6T1 WITH

WB6T2 0.183 0.018 10.228 0.000 0.542

WB6T3 0.137 0.018 7.749 0.000 0.611

WB6T2 WITH

WB6T3 0.178 0.017 10.657 0.000 0.570

WB7T1 WITH

WB7T2 0.196 0.022 8.998 0.000 0.588

WB7T3 0.127 0.023 5.441 0.000 0.621

WB7T2 WITH

WB7T3 0.186 0.021 8.661 0.000 0.620

Means

RI_PA 0.000 0.000 999.000 999.000 0.000

RI_SL 0.000 0.000 999.000 999.000 0.000

RI_WB 0.000 0.000 999.000 999.000 0.000

W_PAT1 0.000 0.000 999.000 999.000 0.000

W_PAT2 0.000 0.000 999.000 999.000 0.000

W_PAT3 0.000 0.000 999.000 999.000 0.000

W_SLT1 0.000 0.000 999.000 999.000 0.000

W_SLT2 0.000 0.000 999.000 999.000 0.000

W_SLT3 0.000 0.000 999.000 999.000 0.000

W_WBT1 0.000 0.000 999.000 999.000 0.000

W_WBT2 0.000 0.000 999.000 999.000 0.000

W_WBT3 0.000 0.000 999.000 999.000 0.000

Intercepts

PAT1 3.805 0.034 111.719 0.000 0.325

PAT2 3.754 0.035 106.786 0.000 0.464

PAT3 3.634 0.034 106.610 0.000 0.422

WBT1 -0.421 0.028 -14.962 0.000 0.153

WBT2 -0.522 0.031 -16.883 0.000 0.210

WBT3 -0.469 0.029 -15.995 0.000 0.231

Thresholds

WB1T1$1 -1.888 0.036 -53.084 0.000 0.320

WB1T1$2 -1.009 0.024 -42.359 0.000 0.272

WB1T1$3 0.016 0.017 0.968 0.333 0.229

WB1T1$4 1.113 0.024 46.449 0.000 0.329

WB2T1$1 -2.157 0.040 -53.269 0.000 0.399

WB2T1$2 -1.125 0.027 -41.211 0.000 0.281

WB2T1$3 0.068 0.021 3.319 0.001 0.207

WB2T1$4 1.431 0.030 48.419 0.000 0.387

WB3T1$1 -2.355 0.039 -60.882 0.000 0.314

WB3T1$2 -1.160 0.026 -43.860 0.000 0.245

WB3T1$3 0.032 0.022 1.442 0.149 0.225

WB3T1$4 1.410 0.029 48.053 0.000 0.330

WB4T1$1 -2.079 0.042 -49.009 0.000 0.356

WB4T1$2 -1.112 0.029 -38.240 0.000 0.288

WB4T1$3 -0.005 0.021 -0.242 0.809 0.195

WB4T1$4 1.294 0.031 41.779 0.000 0.330

WB5T1$1 -2.776 0.053 -52.754 0.000 0.458

WB5T1$2 -1.491 0.037 -40.553 0.000 0.358

WB5T1$3 -0.100 0.029 -3.439 0.001 0.219

WB5T1$4 1.460 0.038 38.557 0.000 0.305

WB6T1$1 -2.240 0.039 -57.624 0.000 0.352

WB6T1$2 -1.440 0.029 -50.004 0.000 0.301

WB6T1$3 -0.480 0.020 -24.115 0.000 0.255

WB6T1$4 0.723 0.020 36.686 0.000 0.274

WB7T1$1 -2.637 0.046 -57.136 0.000 0.406

WB7T1$2 -1.701 0.034 -50.299 0.000 0.384

WB7T1$3 -0.591 0.025 -23.806 0.000 0.299

WB7T1$4 0.728 0.025 29.123 0.000 0.242

WB1T2$1 -1.888 0.036 -53.084 0.000 0.320

WB1T2$2 -1.009 0.024 -42.359 0.000 0.272

WB1T2$3 0.016 0.017 0.968 0.333 0.229

WB1T2$4 1.113 0.024 46.449 0.000 0.329

WB2T2$1 -2.157 0.040 -53.269 0.000 0.399

WB2T2$2 -1.125 0.027 -41.211 0.000 0.281

WB2T2$3 0.068 0.021 3.319 0.001 0.207

WB2T2$4 1.431 0.030 48.419 0.000 0.387

WB3T2$1 -2.355 0.039 -60.882 0.000 0.314

WB3T2$2 -1.160 0.026 -43.860 0.000 0.245

WB3T2$3 0.032 0.022 1.442 0.149 0.225

WB3T2$4 1.410 0.029 48.053 0.000 0.330

WB4T2$1 -2.079 0.042 -49.009 0.000 0.356

WB4T2$2 -1.112 0.029 -38.240 0.000 0.288

WB4T2$3 -0.005 0.021 -0.242 0.809 0.195

WB4T2$4 1.294 0.031 41.779 0.000 0.330

WB5T2$1 -2.776 0.053 -52.754 0.000 0.458

WB5T2$2 -1.491 0.037 -40.553 0.000 0.358

WB5T2$3 -0.100 0.029 -3.439 0.001 0.219

WB5T2$4 1.460 0.038 38.557 0.000 0.305

WB6T2$1 -2.240 0.039 -57.624 0.000 0.352

WB6T2$2 -1.440 0.029 -50.004 0.000 0.301

WB6T2$3 -0.480 0.020 -24.115 0.000 0.255

WB6T2$4 0.723 0.020 36.686 0.000 0.274

WB7T2$1 -2.637 0.046 -57.136 0.000 0.406

WB7T2$2 -1.701 0.034 -50.299 0.000 0.384

WB7T2$3 -0.591 0.025 -23.806 0.000 0.299

WB7T2$4 0.728 0.025 29.123 0.000 0.242

WB1T3$1 -1.888 0.036 -53.084 0.000 0.320

WB1T3$2 -1.009 0.024 -42.359 0.000 0.272

WB1T3$3 0.016 0.017 0.968 0.333 0.229

WB1T3$4 1.113 0.024 46.449 0.000 0.329

WB2T3$1 -2.157 0.040 -53.269 0.000 0.399

WB2T3$2 -1.125 0.027 -41.211 0.000 0.281

WB2T3$3 0.068 0.021 3.319 0.001 0.207

WB2T3$4 1.431 0.030 48.419 0.000 0.387

WB3T3$1 -2.355 0.039 -60.882 0.000 0.314

WB3T3$2 -1.160 0.026 -43.860 0.000 0.245

WB3T3$3 0.032 0.022 1.442 0.149 0.225

WB3T3$4 1.410 0.029 48.053 0.000 0.330

WB4T3$1 -2.079 0.042 -49.009 0.000 0.356

WB4T3$2 -1.112 0.029 -38.240 0.000 0.288

WB4T3$3 -0.005 0.021 -0.242 0.809 0.195

WB4T3$4 1.294 0.031 41.779 0.000 0.330

WB5T3$1 -2.776 0.053 -52.754 0.000 0.458

WB5T3$2 -1.491 0.037 -40.553 0.000 0.358

WB5T3$3 -0.100 0.029 -3.439 0.001 0.219

WB5T3$4 1.460 0.038 38.557 0.000 0.305

WB6T3$1 -2.240 0.039 -57.624 0.000 0.352

WB6T3$2 -1.440 0.029 -50.004 0.000 0.301

WB6T3$3 -0.480 0.020 -24.115 0.000 0.255

WB6T3$4 0.723 0.020 36.686 0.000 0.274

WB7T3$1 -2.637 0.046 -57.136 0.000 0.406

WB7T3$2 -1.701 0.034 -50.299 0.000 0.384

WB7T3$3 -0.591 0.025 -23.806 0.000 0.299

WB7T3$4 0.728 0.025 29.123 0.000 0.242

SLT1$1 -0.426 0.024 -17.999 0.000 0.380

SLT2$1 -0.242 0.026 -9.427 0.000 0.541

SLT3$1 -0.108 0.024 -4.559 0.000 0.430

Variances

RI_PA 1.816 0.076 23.739 0.000 0.398

RI_SL 0.808 0.077 10.514 0.000 0.553

RI_WB 0.387 0.025 15.483 0.000 0.422

W_PAT1 2.911 0.086 33.811 0.000 0.294

W_PAT2 2.575 0.095 27.203 0.000 0.252

W_PAT3 2.565 0.067 38.048 0.000 0.235

W_SLT1 1.000 0.000 999.000 999.000 0.000

W_SLT2 1.000 0.000 999.000 999.000 0.000

W_SLT3 1.000 0.000 999.000 999.000 0.000

W_WBT1 0.674 0.035 19.110 0.000 0.473

W_WBT2 0.653 0.030 21.496 0.000 0.419

W_WBT3 0.726 0.032 22.365 0.000 0.459

Residual Variances

WB1T1 0.943 0.044 21.206 0.000 0.322

WB2T1 0.991 0.043 22.848 0.000 0.397

WB3T1 0.894 0.034 26.677 0.000 0.319

WB4T1 0.939 0.042 22.276 0.000 0.269

WB5T1 0.811 0.034 24.098 0.000 0.398

WB6T1 0.964 0.040 24.360 0.000 0.345

WB7T1 0.993 0.040 24.781 0.000 0.347

WB1T2 0.733 0.035 20.927 0.000 0.412

WB2T2 0.772 0.036 21.315 0.000 0.472

WB3T2 0.827 0.034 24.143 0.000 0.427

WB4T2 0.841 0.040 20.768 0.000 0.452

WB5T2 0.710 0.033 21.534 0.000 0.481

WB6T2 0.851 0.036 23.856 0.000 0.393

WB7T2 0.942 0.042 22.237 0.000 0.473

WB1T3 0.603 0.028 21.582 0.000 0.436

WB2T3 0.622 0.030 21.094 0.000 0.534

WB3T3 0.788 0.032 24.556 0.000 0.437

WB4T3 0.678 0.031 21.661 0.000 0.408

WB5T3 0.679 0.033 20.341 0.000 0.571

WB6T3 0.752 0.033 23.021 0.000 0.471

WB7T3 0.878 0.036 24.064 0.000 0.396

SLT1 0.000 0.000 999.000 999.000 0.000

SLT2 0.000 0.000 999.000 999.000 0.000

SLT3 0.000 0.000 999.000 999.000 0.000

PAT1 0.000 0.000 999.000 999.000 0.000

PAT2 0.000 0.000 999.000 999.000 0.000

PAT3 0.000 0.000 999.000 999.000 0.000

WBT1 0.000 0.000 999.000 999.000 0.000

WBT2 0.000 0.000 999.000 999.000 0.000

WBT3 0.000 0.000 999.000 999.000 0.000

**Model B: Multigroup model (participated in 2 waves at least):**

STANDARDIZED MODEL RESULTS

STDYX Standardization

Two-Tailed Rate of

Estimate S.E. Est./S.E. P-Value Missing

Group MALE

WBT1 BY

WB1T1 0.591 0.009 68.372 0.000 0.304

WB2T1 0.705 0.007 98.415 0.000 0.252

WB3T1 0.711 0.008 87.958 0.000 0.264

WB4T1 0.713 0.007 102.495 0.000 0.258

WB5T1 0.805 0.006 130.190 0.000 0.279

WB6T1 0.627 0.007 86.818 0.000 0.271

WB7T1 0.718 0.007 100.767 0.000 0.308

WBT2 BY

WB1T2 0.665 0.007 89.603 0.000 0.245

WB2T2 0.771 0.006 128.088 0.000 0.279

WB3T2 0.745 0.007 113.937 0.000 0.215

WB4T2 0.739 0.007 112.440 0.000 0.219

WB5T2 0.836 0.005 155.072 0.000 0.272

WB6T2 0.673 0.007 90.787 0.000 0.307

WB7T2 0.754 0.006 117.065 0.000 0.252

WBT3 BY

WB1T3 0.719 0.006 113.586 0.000 0.273

WB2T3 0.826 0.005 152.551 0.000 0.264

WB3T3 0.781 0.006 127.781 0.000 0.267

WB4T3 0.800 0.005 148.439 0.000 0.266

WB5T3 0.865 0.004 195.229 0.000 0.309

WB6T3 0.747 0.006 118.579 0.000 0.288

WB7T3 0.790 0.006 128.499 0.000 0.267

RI_PA BY

PAT1 0.606 0.014 44.533 0.000 0.347

PAT2 0.613 0.016 38.438 0.000 0.276

PAT3 0.614 0.014 43.957 0.000 0.364

RI_SL BY

SLT1 0.375 0.131 2.867 0.004 0.360

SLT2 0.336 0.126 2.664 0.008 0.380

SLT3 0.331 0.124 2.666 0.008 0.385

W_PAT1 BY

PAT1 0.787 0.011 74.846 0.000 0.339

W_PAT2 BY

PAT2 0.782 0.013 62.136 0.000 0.270

W_PAT3 BY

PAT3 0.781 0.011 71.128 0.000 0.359

W_SLT1 BY

SLT1 0.918 0.047 19.627 0.000 0.476

W_SLT2 BY

SLT2 0.934 0.041 22.574 0.000 0.481

W_SLT3 BY

SLT3 0.936 0.040 23.299 0.000 0.486

RI_WB BY

WBT1 0.602 0.020 30.342 0.000 0.383

WBT2 0.571 0.020 28.217 0.000 0.366

WBT3 0.523 0.017 30.390 0.000 0.381

W_WBT1 BY

WBT1 0.788 0.015 52.151 0.000 0.379

W_WBT2 BY

WBT2 0.813 0.014 57.097 0.000 0.364

W_WBT3 BY

WBT3 0.845 0.011 79.260 0.000 0.376

W_PAT2 ON

W_PAT1 0.148 0.027 5.488 0.000 0.438

W_SLT1 0.011 0.043 0.263 0.792 0.573

W_WBT1 0.041 0.026 1.585 0.113 0.361

W_SLT2 ON

W_PAT1 -0.028 0.030 -0.944 0.345 0.399

W_SLT1 0.490 0.060 8.194 0.000 0.449

W_WBT1 -0.032 0.036 -0.884 0.377 0.405

W_WBT2 ON

W_PAT1 0.021 0.025 0.846 0.398 0.311

W_SLT1 0.053 0.043 1.231 0.218 0.506

W_WBT1 0.164 0.037 4.483 0.000 0.405

W_PAT3 ON

W_PAT2 0.188 0.028 6.630 0.000 0.417

W_SLT2 0.005 0.039 0.127 0.899 0.530

W_WBT2 0.048 0.027 1.784 0.075 0.394

W_SLT3 ON

W_PAT2 -0.017 0.028 -0.611 0.541 0.532

W_SLT2 0.515 0.056 9.215 0.000 0.509

W_WBT2 -0.028 0.030 -0.954 0.340 0.488

W_WBT3 ON

W_PAT2 0.009 0.020 0.453 0.651 0.363

W_SLT2 0.081 0.037 2.183 0.029 0.413

W_WBT2 0.227 0.028 8.111 0.000 0.337

WBT1 ON

ETH 0.086 0.008 10.932 0.000 0.096

FSM -0.058 0.007 -8.048 0.000 0.140

SEN -0.065 0.008 -7.991 0.000 0.132

WBT2 ON

ETH 0.081 0.008 10.744 0.000 0.108

FSM -0.055 0.007 -8.041 0.000 0.141

SEN -0.062 0.008 -8.069 0.000 0.129

WBT3 ON

ETH 0.075 0.007 10.694 0.000 0.095

FSM -0.050 0.006 -7.994 0.000 0.137

SEN -0.056 0.007 -8.098 0.000 0.137

PAT1 ON

ETH -0.087 0.008 -10.969 0.000 0.165

FSM -0.039 0.007 -5.626 0.000 0.140

SEN -0.055 0.007 -7.561 0.000 0.101

PAT2 ON

ETH -0.088 0.008 -11.062 0.000 0.167

FSM -0.039 0.007 -5.645 0.000 0.139

SEN -0.056 0.007 -7.495 0.000 0.100

PAT3 ON

ETH -0.088 0.008 -11.122 0.000 0.165

FSM -0.039 0.007 -5.647 0.000 0.138

SEN -0.056 0.007 -7.559 0.000 0.099

SLT1 ON

ETH 0.096 0.011 8.974 0.000 0.146

FSM -0.064 0.009 -6.951 0.000 0.142

SEN -0.026 0.010 -2.521 0.012 0.157

SLT2 ON

ETH 0.085 0.009 9.263 0.000 0.120

FSM -0.057 0.008 -7.092 0.000 0.142

SEN -0.023 0.009 -2.555 0.011 0.142

SLT3 ON

ETH 0.084 0.009 9.317 0.000 0.123

FSM -0.056 0.008 -7.093 0.000 0.141

SEN -0.022 0.009 -2.552 0.011 0.140

RI_PA WITH

RI_SL 0.293 0.442 0.663 0.507 0.091

RI_WB 0.276 0.035 7.852 0.000 0.317

RI_SL WITH

RI_WB 1.142 1.819 0.628 0.530 0.050

W_PAT1 WITH

W_WBT1 0.116 0.026 4.502 0.000 0.305

W_SLT1 0.045 0.041 1.093 0.274 0.498

W_WBT1 WITH

W_SLT1 0.281 0.047 5.982 0.000 0.499

W_PAT2 WITH

W_SLT2 0.021 0.026 0.824 0.410 0.294

W_WBT2 0.141 0.021 6.649 0.000 0.241

W_SLT2 WITH

W_WBT2 0.261 0.029 8.964 0.000 0.325

W_PAT3 WITH

W_SLT3 0.067 0.024 2.788 0.005 0.349

W_WBT3 0.144 0.016 9.058 0.000 0.247

W_SLT3 WITH

W_WBT3 0.269 0.020 13.190 0.000 0.340

WB1T1 WITH

WB1T2 0.256 0.018 14.513 0.000 0.357

WB1T3 0.190 0.020 9.353 0.000 0.441

WB1T2 WITH

WB1T3 0.334 0.019 17.367 0.000 0.424

WB2T1 WITH

WB2T2 0.158 0.022 7.214 0.000 0.481

WB2T3 0.096 0.025 3.844 0.000 0.424

WB2T2 WITH

WB2T3 0.194 0.025 7.882 0.000 0.416

WB3T1 WITH

WB3T2 0.190 0.022 8.690 0.000 0.365

WB3T3 0.168 0.024 7.069 0.000 0.378

WB3T2 WITH

WB3T3 0.215 0.023 9.439 0.000 0.456

WB4T1 WITH

WB4T2 0.123 0.023 5.339 0.000 0.391

WB4T3 0.093 0.023 3.990 0.000 0.377

WB4T2 WITH

WB4T3 0.090 0.024 3.800 0.000 0.437

WB5T1 WITH

WB5T2 0.117 0.027 4.362 0.000 0.372

WB5T3 0.168 0.034 4.952 0.000 0.399

WB5T2 WITH

WB5T3 0.181 0.033 5.452 0.000 0.479

WB6T1 WITH

WB6T2 0.219 0.020 10.796 0.000 0.412

WB6T3 0.174 0.023 7.624 0.000 0.437

WB6T2 WITH

WB6T3 0.233 0.021 11.167 0.000 0.417

WB7T1 WITH

WB7T2 0.186 0.024 7.871 0.000 0.357

WB7T3 0.098 0.028 3.526 0.000 0.479

WB7T2 WITH

WB7T3 0.169 0.025 6.731 0.000 0.436

Means

RI_PA 0.000 0.000 0.000 1.000 0.000

RI_SL 0.000 0.000 0.000 1.000 0.000

RI_WB 0.000 0.000 0.000 1.000 0.000

W_PAT1 0.000 0.000 0.000 1.000 0.000

W_PAT2 0.000 0.000 0.000 1.000 0.000

W_PAT3 0.000 0.000 0.000 1.000 0.000

W_SLT1 0.000 0.000 0.000 1.000 0.000

W_SLT2 0.000 0.000 0.000 1.000 0.000

W_SLT3 0.000 0.000 0.000 1.000 0.000

W_WBT1 0.000 0.000 0.000 1.000 0.000

W_WBT2 0.000 0.000 0.000 1.000 0.000

W_WBT3 0.000 0.000 0.000 1.000 0.000

Intercepts

PAT1 1.626 0.028 57.267 0.000 0.124

PAT2 1.627 0.027 61.081 0.000 0.149

PAT3 1.580 0.028 55.582 0.000 0.108

WBT1 0.000 0.000 0.000 1.000 0.000

WBT2 -0.035 0.022 -1.600 0.110 0.273

WBT3 -0.040 0.022 -1.833 0.067 0.156

Thresholds

WB1T1$1 -1.577 0.028 -57.213 0.000 0.160

WB1T1$2 -0.852 0.020 -43.083 0.000 0.132

WB1T1$3 -0.014 0.016 -0.907 0.364 0.131

WB1T1$4 0.894 0.020 44.754 0.000 0.188

WB2T1$1 -1.583 0.026 -61.744 0.000 0.181

WB2T1$2 -0.839 0.019 -43.706 0.000 0.142

WB2T1$3 0.024 0.017 1.463 0.143 0.125

WB2T1$4 1.011 0.020 50.013 0.000 0.147

WB3T1$1 -1.673 0.025 -68.199 0.000 0.194

WB3T1$2 -0.840 0.019 -44.213 0.000 0.175

WB3T1$3 0.000 0.018 -0.014 0.989 0.151

WB3T1$4 0.974 0.020 48.192 0.000 0.145

WB4T1$1 -1.519 0.026 -57.316 0.000 0.188

WB4T1$2 -0.826 0.020 -41.845 0.000 0.158

WB4T1$3 -0.027 0.017 -1.628 0.104 0.123

WB4T1$4 0.909 0.022 41.422 0.000 0.114

WB5T1$1 -1.658 0.025 -65.687 0.000 0.219

WB5T1$2 -0.912 0.021 -44.273 0.000 0.175

WB5T1$3 -0.092 0.019 -4.945 0.000 0.124

WB5T1$4 0.832 0.021 38.997 0.000 0.105

WB6T1$1 -1.788 0.027 -65.093 0.000 0.227

WB6T1$2 -1.164 0.022 -52.614 0.000 0.208

WB6T1$3 -0.404 0.017 -24.174 0.000 0.141

WB6T1$4 0.554 0.017 31.967 0.000 0.138

WB7T1$1 -1.885 0.027 -69.839 0.000 0.195

WB7T1$2 -1.227 0.021 -59.466 0.000 0.190

WB7T1$3 -0.443 0.018 -24.842 0.000 0.166

WB7T1$4 0.494 0.019 26.451 0.000 0.122

WB1T2$1 -1.683 0.025 -67.287 0.000 0.176

WB1T2$2 -0.909 0.019 -48.007 0.000 0.170

WB1T2$3 -0.015 0.017 -0.909 0.364 0.133

WB1T2$4 0.953 0.021 45.098 0.000 0.148

WB2T2$1 -1.641 0.025 -66.023 0.000 0.157

WB2T2$2 -0.870 0.019 -46.315 0.000 0.114

WB2T2$3 0.025 0.017 1.458 0.145 0.125

WB2T2$4 1.049 0.023 45.274 0.000 0.137

WB3T2$1 -1.661 0.023 -73.116 0.000 0.189

WB3T2$2 -0.834 0.018 -47.330 0.000 0.160

WB3T2$3 0.000 0.017 -0.014 0.989 0.151

WB3T2$4 0.968 0.021 45.692 0.000 0.154

WB4T2$1 -1.491 0.025 -60.542 0.000 0.130

WB4T2$2 -0.811 0.019 -42.606 0.000 0.130

WB4T2$3 -0.027 0.016 -1.628 0.103 0.122

WB4T2$4 0.893 0.021 41.765 0.000 0.112

WB5T2$1 -1.631 0.026 -62.030 0.000 0.147

WB5T2$2 -0.897 0.021 -43.188 0.000 0.126

WB5T2$3 -0.091 0.018 -4.945 0.000 0.118

WB5T2$4 0.818 0.022 37.649 0.000 0.148

WB6T2$1 -1.821 0.027 -68.238 0.000 0.158

WB6T2$2 -1.185 0.021 -56.188 0.000 0.157

WB6T2$3 -0.411 0.016 -25.076 0.000 0.111

WB6T2$4 0.564 0.018 31.943 0.000 0.129

WB7T2$1 -1.876 0.027 -69.841 0.000 0.162

WB7T2$2 -1.221 0.021 -57.944 0.000 0.163

WB7T2$3 -0.441 0.018 -24.859 0.000 0.141

WB7T2$4 0.492 0.018 26.581 0.000 0.133

WB1T3$1 -1.670 0.028 -59.809 0.000 0.158

WB1T3$2 -0.903 0.020 -44.413 0.000 0.146

WB1T3$3 -0.015 0.016 -0.908 0.364 0.132

WB1T3$4 0.946 0.021 44.709 0.000 0.148

WB2T3$1 -1.614 0.025 -63.285 0.000 0.214

WB2T3$2 -0.855 0.019 -45.148 0.000 0.159

WB2T3$3 0.025 0.017 1.460 0.144 0.124

WB2T3$4 1.031 0.022 46.433 0.000 0.123

WB3T3$1 -1.597 0.024 -66.772 0.000 0.205

WB3T3$2 -0.802 0.018 -44.734 0.000 0.182

WB3T3$3 0.000 0.017 -0.014 0.989 0.151

WB3T3$4 0.930 0.020 46.653 0.000 0.122

WB4T3$1 -1.481 0.024 -62.292 0.000 0.147

WB4T3$2 -0.806 0.019 -43.284 0.000 0.129

WB4T3$3 -0.027 0.016 -1.628 0.104 0.123

WB4T3$4 0.887 0.021 42.408 0.000 0.125

WB5T3$1 -1.548 0.025 -61.368 0.000 0.167

WB5T3$2 -0.852 0.020 -42.080 0.000 0.147

WB5T3$3 -0.086 0.017 -4.944 0.000 0.121

WB5T3$4 0.777 0.021 37.269 0.000 0.123

WB6T3$1 -1.852 0.026 -72.439 0.000 0.189

WB6T3$2 -1.205 0.021 -58.239 0.000 0.175

WB6T3$3 -0.418 0.016 -25.351 0.000 0.134

WB6T3$4 0.574 0.018 31.247 0.000 0.116

WB7T3$1 -1.804 0.026 -68.264 0.000 0.157

WB7T3$2 -1.174 0.021 -57.044 0.000 0.162

WB7T3$3 -0.424 0.017 -24.621 0.000 0.150

WB7T3$4 0.473 0.018 26.850 0.000 0.133

SLT1$1 -0.431 0.023 -18.365 0.000 0.329

SLT2$1 -0.248 0.022 -11.438 0.000 0.351

SLT3$1 -0.106 0.021 -4.949 0.000 0.226

Variances

RI_PA 1.000 0.000 ********* 0.000 0.000

RI_SL 1.000 0.000 ********* 0.000 0.000

RI_WB 1.000 0.000 ********* 0.000 0.000

W_PAT1 1.000 0.000 ********* 0.000 0.000

W_PAT2 0.973 0.009 103.345 0.000 0.416

W_PAT3 0.958 0.012 82.278 0.000 0.405

W_SLT1 1.000 0.000 ********* 0.000 0.000

W_SLT2 0.764 0.057 13.447 0.000 0.444

W_SLT3 0.739 0.054 13.646 0.000 0.506

W_WBT1 1.000 0.000 ********* 0.000 0.000

W_WBT2 0.962 0.014 66.635 0.000 0.351

W_WBT3 0.930 0.015 60.728 0.000 0.390

Residual Variances

WB1T1 0.651 0.010 63.811 0.000 0.304

WB2T1 0.504 0.010 49.914 0.000 0.252

WB3T1 0.494 0.012 42.939 0.000 0.264

WB4T1 0.492 0.010 49.538 0.000 0.258

WB5T1 0.352 0.010 35.357 0.000 0.278

WB6T1 0.607 0.009 67.101 0.000 0.271

WB7T1 0.485 0.010 47.371 0.000 0.308

WB1T2 0.558 0.010 56.561 0.000 0.244

WB2T2 0.405 0.009 43.683 0.000 0.279

WB3T2 0.444 0.010 45.537 0.000 0.215

WB4T2 0.454 0.010 46.754 0.000 0.219

WB5T2 0.301 0.009 33.435 0.000 0.272

WB6T2 0.547 0.010 54.763 0.000 0.307

WB7T2 0.432 0.010 44.493 0.000 0.252

WB1T3 0.482 0.009 52.945 0.000 0.273

WB2T3 0.317 0.009 35.444 0.000 0.264

WB3T3 0.389 0.010 40.735 0.000 0.267

WB4T3 0.360 0.009 41.775 0.000 0.267

WB5T3 0.252 0.008 32.848 0.000 0.309

WB6T3 0.442 0.009 47.013 0.000 0.288

WB7T3 0.376 0.010 38.651 0.000 0.267

SLT1 0.000 999.000 999.000 999.000 0.000

SLT2 0.000 999.000 999.000 999.000 0.000

SLT3 0.000 999.000 999.000 999.000 0.000

PAT1 0.000 999.000 999.000 999.000 0.000

PAT2 0.000 999.000 999.000 999.000 0.000

PAT3 0.000 999.000 999.000 999.000 0.000

WBT1 0.000 999.000 999.000 999.000 0.000

WBT2 0.000 999.000 999.000 999.000 0.000

WBT3 0.000 999.000 999.000 999.000 0.000

Group FEMALE

WBT1 BY

WB1T1 0.600 0.009 70.095 0.000 0.218

WB2T1 0.708 0.008 91.278 0.000 0.205

WB3T1 0.734 0.006 116.964 0.000 0.302

WB4T1 0.725 0.007 102.692 0.000 0.225

WB5T1 0.840 0.005 174.768 0.000 0.345

WB6T1 0.632 0.008 77.706 0.000 0.252

WB7T1 0.715 0.006 110.137 0.000 0.233

WBT2 BY

WB1T2 0.654 0.008 83.403 0.000 0.218

WB2T2 0.751 0.007 107.802 0.000 0.295

WB3T2 0.752 0.006 116.981 0.000 0.332

WB4T2 0.739 0.006 114.888 0.000 0.253

WB5T2 0.858 0.005 173.432 0.000 0.394

WB6T2 0.656 0.008 84.795 0.000 0.306

WB7T2 0.724 0.007 107.594 0.000 0.222

WBT3 BY

WB1T3 0.701 0.007 100.711 0.000 0.344

WB2T3 0.793 0.006 135.772 0.000 0.415

WB3T3 0.767 0.006 133.480 0.000 0.360

WB4T3 0.787 0.006 131.524 0.000 0.412

WB5T3 0.867 0.004 195.979 0.000 0.427

WB6T3 0.689 0.007 97.724 0.000 0.310

WB7T3 0.746 0.006 117.578 0.000 0.239

RI_PA BY

PAT1 0.616 0.013 46.442 0.000 0.358

PAT2 0.637 0.016 39.398 0.000 0.290

PAT3 0.632 0.014 44.860 0.000 0.369

RI_SL BY

SLT1 0.673 0.018 37.826 0.000 0.387

SLT2 0.663 0.021 31.861 0.000 0.389

SLT3 0.664 0.021 31.873 0.000 0.391

W_PAT1 BY

PAT1 0.779 0.011 73.467 0.000 0.350

W_PAT2 BY

PAT2 0.762 0.014 55.415 0.000 0.281

W_PAT3 BY

PAT3 0.766 0.012 64.974 0.000 0.361

W_SLT1 BY

SLT1 0.734 0.016 45.429 0.000 0.385

W_SLT2 BY

SLT2 0.743 0.018 40.306 0.000 0.389

W_SLT3 BY

SLT3 0.742 0.018 40.164 0.000 0.389

RI_WB BY

WBT1 0.599 0.019 30.869 0.000 0.361

WBT2 0.595 0.021 28.794 0.000 0.371

WBT3 0.579 0.019 30.218 0.000 0.362

W_WBT1 BY

WBT1 0.792 0.015 54.169 0.000 0.369

W_WBT2 BY

WBT2 0.795 0.015 51.311 0.000 0.377

W_WBT3 BY

WBT3 0.808 0.014 59.193 0.000 0.369

W_PAT2 ON

W_PAT1 0.145 0.029 4.983 0.000 0.370

W_SLT1 0.008 0.039 0.213 0.832 0.350

W_WBT1 0.045 0.033 1.382 0.167 0.426

W_SLT2 ON

W_PAT1 -0.035 0.034 -1.029 0.304 0.440

W_SLT1 0.136 0.055 2.479 0.013 0.376

W_WBT1 0.132 0.042 3.100 0.002 0.381

W_WBT2 ON

W_PAT1 -0.008 0.026 -0.306 0.759 0.404

W_SLT1 0.085 0.041 2.064 0.039 0.442

W_WBT1 0.187 0.039 4.841 0.000 0.492

W_PAT3 ON

W_PAT2 0.165 0.033 4.950 0.000 0.433

W_SLT2 0.016 0.037 0.448 0.654 0.364

W_WBT2 -0.009 0.032 -0.291 0.771 0.416

W_SLT3 ON

W_PAT2 -0.042 0.033 -1.263 0.206 0.345

W_SLT2 0.197 0.050 3.904 0.000 0.368

W_WBT2 0.039 0.037 1.079 0.281 0.431

W_WBT3 ON

W_PAT2 -0.018 0.024 -0.725 0.468 0.360

W_SLT2 0.046 0.034 1.366 0.172 0.349

W_WBT2 0.220 0.032 6.830 0.000 0.452

WBT1 ON

ETH 0.087 0.008 10.943 0.000 0.099

FSM -0.059 0.007 -8.011 0.000 0.141

SEN -0.051 0.006 -7.966 0.000 0.133

WBT2 ON

ETH 0.086 0.008 10.736 0.000 0.106

FSM -0.058 0.007 -7.979 0.000 0.137

SEN -0.051 0.006 -8.027 0.000 0.131

WBT3 ON

ETH 0.084 0.008 10.866 0.000 0.098

FSM -0.057 0.007 -8.028 0.000 0.137

SEN -0.049 0.006 -7.994 0.000 0.137

PAT1 ON

ETH -0.094 0.008 -11.102 0.000 0.172

FSM -0.042 0.007 -5.652 0.000 0.139

SEN -0.046 0.006 -7.525 0.000 0.103

PAT2 ON

ETH -0.097 0.009 -10.970 0.000 0.168

FSM -0.043 0.008 -5.625 0.000 0.138

SEN -0.048 0.006 -7.507 0.000 0.102

PAT3 ON

ETH -0.096 0.009 -11.074 0.000 0.168

FSM -0.043 0.008 -5.615 0.000 0.141

SEN -0.048 0.006 -7.553 0.000 0.105

SLT1 ON

ETH 0.077 0.008 9.156 0.000 0.133

FSM -0.052 0.007 -7.074 0.000 0.142

SEN -0.016 0.006 -2.548 0.011 0.143

SLT2 ON

ETH 0.076 0.008 9.198 0.000 0.131

FSM -0.051 0.007 -7.087 0.000 0.144

SEN -0.016 0.006 -2.550 0.011 0.142

SLT3 ON

ETH 0.076 0.008 9.204 0.000 0.131

FSM -0.051 0.007 -7.092 0.000 0.143

SEN -0.016 0.006 -2.550 0.011 0.143

RI_PA WITH

RI_SL 0.158 0.039 4.092 0.000 0.299

RI_WB 0.248 0.038 6.487 0.000 0.370

RI_SL WITH

RI_WB 0.680 0.034 20.204 0.000 0.404

W_PAT1 WITH

W_WBT1 0.135 0.025 5.361 0.000 0.382

W_SLT1 0.101 0.032 3.183 0.001 0.324

W_WBT1 WITH

W_SLT1 0.466 0.030 15.366 0.000 0.346

W_PAT2 WITH

W_SLT2 0.017 0.031 0.532 0.595 0.329

W_WBT2 0.072 0.026 2.757 0.006 0.343

W_SLT2 WITH

W_WBT2 0.367 0.029 12.791 0.000 0.309

W_PAT3 WITH

W_SLT3 0.065 0.024 2.669 0.008 0.290

W_WBT3 0.076 0.022 3.528 0.000 0.293

W_SLT3 WITH

W_WBT3 0.301 0.024 12.412 0.000 0.380

WB1T1 WITH

WB1T2 0.257 0.018 14.302 0.000 0.464

WB1T3 0.194 0.021 9.368 0.000 0.368

WB1T2 WITH

WB1T3 0.314 0.020 16.045 0.000 0.363

WB2T1 WITH

WB2T2 0.147 0.021 7.120 0.000 0.481

WB2T3 0.133 0.025 5.427 0.000 0.464

WB2T2 WITH

WB2T3 0.157 0.025 6.382 0.000 0.516

WB3T1 WITH

WB3T2 0.219 0.022 9.770 0.000 0.456

WB3T3 0.156 0.023 6.867 0.000 0.425

WB3T2 WITH

WB3T3 0.226 0.023 9.722 0.000 0.427

WB4T1 WITH

WB4T2 0.139 0.021 6.601 0.000 0.360

WB4T3 0.071 0.025 2.826 0.005 0.455

WB4T2 WITH

WB4T3 0.104 0.025 4.230 0.000 0.490

WB5T1 WITH

WB5T2 0.187 0.030 6.180 0.000 0.429

WB5T3 0.175 0.035 4.941 0.000 0.472

WB5T2 WITH

WB5T3 0.203 0.036 5.698 0.000 0.526

WB6T1 WITH

WB6T2 0.198 0.020 9.819 0.000 0.370

WB6T3 0.156 0.020 7.666 0.000 0.449

WB6T2 WITH

WB6T3 0.221 0.020 10.830 0.000 0.401

WB7T1 WITH

WB7T2 0.200 0.021 9.411 0.000 0.360

WB7T3 0.136 0.024 5.715 0.000 0.354

WB7T2 WITH

WB7T3 0.202 0.023 8.728 0.000 0.502

Means

RI_PA 0.000 0.000 0.000 1.000 0.000

RI_SL 0.000 0.000 0.000 1.000 0.000

RI_WB 0.000 0.000 0.000 1.000 0.000

W_PAT1 0.000 0.000 0.000 1.000 0.000

W_PAT2 0.000 0.000 0.000 1.000 0.000

W_PAT3 0.000 0.000 0.000 1.000 0.000

W_SLT1 0.000 0.000 0.000 1.000 0.000

W_SLT2 0.000 0.000 0.000 1.000 0.000

W_SLT3 0.000 0.000 0.000 1.000 0.000

W_WBT1 0.000 0.000 0.000 1.000 0.000

W_WBT2 0.000 0.000 0.000 1.000 0.000

W_WBT3 0.000 0.000 0.000 1.000 0.000

Intercepts

PAT1 1.747 0.030 57.411 0.000 0.108

PAT2 1.785 0.033 53.855 0.000 0.100

PAT3 1.718 0.031 56.226 0.000 0.112

WBT1 -0.437 0.031 -14.001 0.000 0.096

WBT2 -0.546 0.034 -16.210 0.000 0.088

WBT3 -0.495 0.032 -15.640 0.000 0.165

Thresholds

WB1T1$1 -1.610 0.024 -67.406 0.000 0.182

WB1T1$2 -0.870 0.019 -46.679 0.000 0.149

WB1T1$3 -0.014 0.016 -0.908 0.364 0.132

WB1T1$4 0.912 0.020 46.699 0.000 0.179

WB2T1$1 -1.597 0.024 -65.569 0.000 0.160

WB2T1$2 -0.846 0.019 -45.142 0.000 0.128

WB2T1$3 0.025 0.017 1.462 0.144 0.125

WB2T1$4 1.020 0.021 48.577 0.000 0.164

WB3T1$1 -1.732 0.025 -70.075 0.000 0.163

WB3T1$2 -0.870 0.020 -44.162 0.000 0.149

WB3T1$3 0.000 0.018 -0.014 0.989 0.151

WB3T1$4 1.009 0.021 48.983 0.000 0.169

WB4T1$1 -1.550 0.025 -60.934 0.000 0.160

WB4T1$2 -0.843 0.020 -41.813 0.000 0.136

WB4T1$3 -0.028 0.017 -1.625 0.104 0.122

WB4T1$4 0.928 0.020 46.590 0.000 0.127

WB5T1$1 -1.737 0.027 -65.141 0.000 0.165

WB5T1$2 -0.956 0.022 -42.956 0.000 0.128

WB5T1$3 -0.096 0.020 -4.924 0.000 0.118

WB5T1$4 0.872 0.022 40.232 0.000 0.146

WB6T1$1 -1.811 0.026 -69.174 0.000 0.232

WB6T1$2 -1.179 0.020 -58.617 0.000 0.162

WB6T1$3 -0.409 0.016 -24.944 0.000 0.126

WB6T1$4 0.561 0.018 31.822 0.000 0.143

WB7T1$1 -1.885 0.027 -69.336 0.000 0.199

WB7T1$2 -1.227 0.022 -56.487 0.000 0.179

WB7T1$3 -0.443 0.018 -24.181 0.000 0.148

WB7T1$4 0.494 0.018 27.745 0.000 0.127

WB1T2$1 -1.744 0.025 -70.372 0.000 0.175

WB1T2$2 -0.942 0.020 -47.113 0.000 0.160

WB1T2$3 -0.016 0.017 -0.908 0.364 0.132

WB1T2$4 0.988 0.021 46.287 0.000 0.158

WB2T2$1 -1.683 0.026 -64.987 0.000 0.188

WB2T2$2 -0.892 0.020 -45.125 0.000 0.123

WB2T2$3 0.026 0.018 1.462 0.144 0.126

WB2T2$4 1.076 0.022 48.585 0.000 0.187

WB3T2$1 -1.763 0.025 -69.750 0.000 0.166

WB3T2$2 -0.885 0.020 -43.639 0.000 0.145

WB3T2$3 0.000 0.019 -0.014 0.989 0.151

WB3T2$4 1.027 0.021 48.384 0.000 0.183

WB4T2$1 -1.571 0.025 -63.929 0.000 0.138

WB4T2$2 -0.855 0.020 -42.702 0.000 0.118

WB4T2$3 -0.028 0.017 -1.626 0.104 0.121

WB4T2$4 0.941 0.021 45.384 0.000 0.177

WB5T2$1 -1.762 0.027 -64.399 0.000 0.121

WB5T2$2 -0.969 0.023 -42.807 0.000 0.098

WB5T2$3 -0.098 0.020 -4.922 0.000 0.115

WB5T2$4 0.884 0.022 39.730 0.000 0.163

WB6T2$1 -1.868 0.026 -71.069 0.000 0.207

WB6T2$2 -1.216 0.021 -57.537 0.000 0.183

WB6T2$3 -0.422 0.017 -24.835 0.000 0.123

WB6T2$4 0.578 0.018 32.358 0.000 0.122

WB7T2$1 -1.897 0.025 -75.878 0.000 0.120

WB7T2$2 -1.234 0.021 -59.963 0.000 0.113

WB7T2$3 -0.446 0.018 -24.758 0.000 0.127

WB7T2$4 0.497 0.018 27.196 0.000 0.147

WB1T3$1 -1.815 0.028 -65.389 0.000 0.231

WB1T3$2 -0.981 0.022 -45.349 0.000 0.196

WB1T3$3 -0.016 0.018 -0.908 0.364 0.133

WB1T3$4 1.029 0.021 48.393 0.000 0.160

WB2T3$1 -1.727 0.027 -63.216 0.000 0.247

WB2T3$2 -0.915 0.021 -43.546 0.000 0.171

WB2T3$3 0.027 0.018 1.463 0.143 0.124

WB2T3$4 1.103 0.022 49.752 0.000 0.176

WB3T3$1 -1.747 0.025 -70.131 0.000 0.204

WB3T3$2 -0.877 0.019 -45.509 0.000 0.153

WB3T3$3 0.000 0.018 -0.014 0.989 0.151

WB3T3$4 1.018 0.021 48.435 0.000 0.188

WB4T3$1 -1.625 0.025 -63.815 0.000 0.187

WB4T3$2 -0.884 0.021 -42.670 0.000 0.138

WB4T3$3 -0.029 0.018 -1.625 0.104 0.122

WB4T3$4 0.973 0.021 46.418 0.000 0.181

WB5T3$1 -1.730 0.026 -65.589 0.000 0.181

WB5T3$2 -0.952 0.022 -43.355 0.000 0.143

WB5T3$3 -0.096 0.020 -4.924 0.000 0.119

WB5T3$4 0.868 0.022 40.160 0.000 0.160

WB6T3$1 -1.907 0.026 -73.713 0.000 0.238

WB6T3$2 -1.241 0.021 -60.196 0.000 0.191

WB6T3$3 -0.430 0.017 -25.115 0.000 0.132

WB6T3$4 0.590 0.018 32.371 0.000 0.126

WB7T3$1 -1.899 0.026 -73.416 0.000 0.217

WB7T3$2 -1.236 0.021 -58.698 0.000 0.189

WB7T3$3 -0.446 0.018 -24.554 0.000 0.155

WB7T3$4 0.498 0.018 27.689 0.000 0.137

SLT1$1 -0.345 0.018 -18.704 0.000 0.175

SLT2$1 -0.219 0.019 -11.579 0.000 0.329

SLT3$1 -0.096 0.019 -4.963 0.000 0.219

Variances

RI_PA 1.000 0.000 ********* 0.000 0.000

RI_SL 1.000 0.000 ********* 0.000 0.000

RI_WB 1.000 0.000 ********* 0.000 0.000

W_PAT1 1.000 0.000 ********* 0.000 0.000

W_PAT2 0.973 0.010 99.203 0.000 0.376

W_PAT3 0.971 0.011 85.943 0.000 0.395

W_SLT1 1.000 0.000 ********* 0.000 0.000

W_SLT2 0.947 0.020 47.121 0.000 0.386

W_SLT3 0.951 0.020 47.563 0.000 0.382

W_WBT1 1.000 0.000 ********* 0.000 0.000

W_WBT2 0.942 0.016 60.077 0.000 0.413

W_WBT3 0.941 0.015 60.800 0.000 0.429

Residual Variances

WB1T1 0.640 0.010 62.216 0.000 0.218

WB2T1 0.499 0.011 45.445 0.000 0.206

WB3T1 0.462 0.009 50.183 0.000 0.302

WB4T1 0.475 0.010 46.396 0.000 0.224

WB5T1 0.294 0.008 36.332 0.000 0.345

WB6T1 0.600 0.010 58.385 0.000 0.252

WB7T1 0.489 0.009 52.711 0.000 0.233

WB1T2 0.572 0.010 55.669 0.000 0.217

WB2T2 0.436 0.010 41.595 0.000 0.295

WB3T2 0.435 0.010 45.070 0.000 0.331

WB4T2 0.453 0.010 47.613 0.000 0.253

WB5T2 0.264 0.008 31.094 0.000 0.393

WB6T2 0.569 0.010 55.998 0.000 0.305

WB7T2 0.476 0.010 48.816 0.000 0.222

WB1T3 0.508 0.010 52.080 0.000 0.344

WB2T3 0.371 0.009 40.040 0.000 0.415

WB3T3 0.412 0.009 46.798 0.000 0.360

WB4T3 0.380 0.009 40.357 0.000 0.412

WB5T3 0.249 0.008 32.478 0.000 0.427

WB6T3 0.525 0.010 53.917 0.000 0.310

WB7T3 0.444 0.009 46.864 0.000 0.240

SLT1 0.000 999.000 999.000 999.000 0.000

SLT2 0.000 999.000 999.000 999.000 0.000

SLT3 0.000 999.000 999.000 999.000 0.000

PAT1 0.000 999.000 999.000 999.000 0.000

PAT2 0.000 999.000 999.000 999.000 0.000

PAT3 0.000 999.000 999.000 999.000 0.000

WBT1 0.000 999.000 999.000 999.000 0.000

WBT2 0.000 999.000 999.000 999.000 0.000

WBT3 0.000 999.000 999.000 999.000 0.000

UNSTANDARDIZED MODEL RESULTS

Two-Tailed Rate of

Estimate S.E. Est./S.E. P-Value Missing

Group MALE

WBT1 BY

WB1T1 0.723 0.020 35.385 0.000 0.274

WB2T1 0.981 0.025 39.835 0.000 0.321

WB3T1 1.000 0.000 999.000 999.000 0.000

WB4T1 1.005 0.027 36.674 0.000 0.328

WB5T1 1.341 0.034 39.466 0.000 0.359

WB6T1 0.795 0.021 38.646 0.000 0.338

WB7T1 1.019 0.027 38.110 0.000 0.347

WBT2 BY

WB1T2 0.723 0.020 35.385 0.000 0.274

WB2T2 0.981 0.025 39.835 0.000 0.321

WB3T2 1.000 0.000 999.000 999.000 0.000

WB4T2 1.005 0.027 36.674 0.000 0.328

WB5T2 1.341 0.034 39.466 0.000 0.359

WB6T2 0.795 0.021 38.646 0.000 0.338

WB7T2 1.019 0.027 38.110 0.000 0.347

WBT3 BY

WB1T3 0.723 0.020 35.385 0.000 0.274

WB2T3 0.981 0.025 39.835 0.000 0.321

WB3T3 1.000 0.000 999.000 999.000 0.000

WB4T3 1.005 0.027 36.674 0.000 0.328

WB5T3 1.341 0.034 39.466 0.000 0.359

WB6T3 0.795 0.021 38.646 0.000 0.338

WB7T3 1.019 0.027 38.110 0.000 0.347

RI_PA BY

PAT1 1.000 0.000 999.000 999.000 0.000

PAT2 1.000 0.000 999.000 999.000 0.000

PAT3 1.000 0.000 999.000 999.000 0.000

RI_SL BY

SLT1 1.000 0.000 999.000 999.000 0.000

SLT2 1.000 0.000 999.000 999.000 0.000

SLT3 1.000 0.000 999.000 999.000 0.000

W_PAT1 BY

PAT1 1.000 0.000 999.000 999.000 0.000

W_PAT2 BY

PAT2 1.000 0.000 999.000 999.000 0.000

W_PAT3 BY

PAT3 1.000 0.000 999.000 999.000 0.000

W_SLT1 BY

SLT1 1.000 0.000 999.000 999.000 0.000

W_SLT2 BY

SLT2 1.000 0.000 999.000 999.000 0.000

W_SLT3 BY

SLT3 1.000 0.000 999.000 999.000 0.000

RI_WB BY

WBT1 1.000 0.000 999.000 999.000 0.000

WBT2 1.000 0.000 999.000 999.000 0.000

WBT3 1.000 0.000 999.000 999.000 0.000

W_WBT1 BY

WBT1 1.000 0.000 999.000 999.000 0.000

W_WBT2 BY

WBT2 1.000 0.000 999.000 999.000 0.000

W_WBT3 BY

WBT3 1.000 0.000 999.000 999.000 0.000

W_PAT2 ON

W_PAT1 0.145 0.027 5.311 0.000 0.409

W_SLT1 0.020 0.077 0.264 0.792 0.573

W_WBT1 0.093 0.059 1.583 0.113 0.358

W_SLT2 ON

W_PAT1 -0.018 0.019 -0.945 0.345 0.395

W_SLT1 0.562 0.089 6.316 0.000 0.446

W_WBT1 -0.045 0.051 -0.885 0.376 0.404

W_WBT2 ON

W_PAT1 0.010 0.012 0.845 0.398 0.313

W_SLT1 0.046 0.038 1.228 0.219 0.504

W_WBT1 0.178 0.040 4.422 0.000 0.390

W_PAT3 ON

W_PAT2 0.188 0.028 6.748 0.000 0.437

W_SLT2 0.008 0.062 0.125 0.901 0.530

W_WBT2 0.100 0.056 1.784 0.074 0.395

W_SLT3 ON

W_PAT2 -0.011 0.018 -0.608 0.543 0.536

W_SLT2 0.523 0.059 8.857 0.000 0.510

W_WBT2 -0.038 0.040 -0.954 0.340 0.490

W_WBT3 ON

W_PAT2 0.005 0.011 0.452 0.651 0.364

W_SLT2 0.070 0.031 2.214 0.027 0.417

W_WBT2 0.257 0.031 8.319 0.000 0.343

WBT1 ON

ETH 0.183 0.017 10.713 0.000 0.111

FSM -0.136 0.017 -8.028 0.000 0.140

SEN -0.169 0.022 -7.856 0.000 0.132

WBT2 ON

ETH 0.183 0.017 10.713 0.000 0.111

FSM -0.136 0.017 -8.028 0.000 0.140

SEN -0.169 0.022 -7.856 0.000 0.132

WBT3 ON

ETH 0.183 0.017 10.713 0.000 0.111

FSM -0.136 0.017 -8.028 0.000 0.140

SEN -0.169 0.022 -7.856 0.000 0.132

PAT1 ON

ETH -0.426 0.039 -11.061 0.000 0.171

FSM -0.210 0.037 -5.642 0.000 0.140

SEN -0.331 0.044 -7.552 0.000 0.103

PAT2 ON

ETH -0.426 0.039 -11.061 0.000 0.171

FSM -0.210 0.037 -5.642 0.000 0.140

SEN -0.331 0.044 -7.552 0.000 0.103

PAT3 ON

ETH -0.426 0.039 -11.061 0.000 0.171

FSM -0.210 0.037 -5.642 0.000 0.140

SEN -0.331 0.044 -7.552 0.000 0.103

SLT1 ON

ETH 0.219 0.024 9.137 0.000 0.126

FSM -0.163 0.023 -7.030 0.000 0.149

SEN -0.072 0.028 -2.551 0.011 0.141

SLT2 ON

ETH 0.219 0.024 9.137 0.000 0.126

FSM -0.163 0.023 -7.030 0.000 0.149

SEN -0.072 0.028 -2.551 0.011 0.141

SLT3 ON

ETH 0.219 0.024 9.137 0.000 0.126

FSM -0.163 0.023 -7.030 0.000 0.149

SEN -0.072 0.028 -2.551 0.011 0.141

RI_PA WITH

RI_SL 0.160 0.073 2.198 0.028 0.553

RI_WB 0.239 0.033 7.271 0.000 0.291

RI_SL WITH

RI_WB 0.267 0.044 6.028 0.000 0.435

W_PAT1 WITH

W_WBT1 0.171 0.040 4.226 0.000 0.298

W_SLT1 0.083 0.076 1.091 0.275 0.497

W_WBT1 WITH

W_SLT1 0.224 0.039 5.750 0.000 0.477

W_PAT2 WITH

W_SLT2 0.038 0.046 0.823 0.410 0.294

W_WBT2 0.214 0.034 6.311 0.000 0.230

W_SLT2 WITH

W_WBT2 0.222 0.026 8.468 0.000 0.298

W_PAT3 WITH

W_SLT3 0.118 0.042 2.782 0.005 0.350

W_WBT3 0.242 0.030 8.120 0.000 0.236

W_SLT3 WITH

W_WBT3 0.255 0.021 12.286 0.000 0.321

WB1T1 WITH

WB1T2 0.222 0.016 13.527 0.000 0.394

WB1T3 0.154 0.017 9.012 0.000 0.439

WB1T2 WITH

WB1T3 0.236 0.016 14.614 0.000 0.373

WB2T1 WITH

WB2T2 0.136 0.019 7.124 0.000 0.452

WB2T3 0.075 0.020 3.812 0.000 0.413

WB2T2 WITH

WB2T3 0.131 0.017 7.532 0.000 0.406

WB3T1 WITH

WB3T2 0.181 0.021 8.494 0.000 0.363

WB3T3 0.157 0.023 6.857 0.000 0.377

WB3T2 WITH

WB3T3 0.191 0.021 9.282 0.000 0.409

WB4T1 WITH

WB4T2 0.120 0.022 5.343 0.000 0.394

WB4T3 0.082 0.020 4.000 0.000 0.375

WB4T2 WITH

WB4T3 0.077 0.020 3.805 0.000 0.428

WB5T1 WITH

WB5T2 0.110 0.025 4.334 0.000 0.379

WB5T3 0.152 0.031 4.868 0.000 0.401

WB5T2 WITH

WB5T3 0.154 0.029 5.395 0.000 0.489

WB6T1 WITH

WB6T2 0.204 0.019 10.552 0.000 0.420

WB6T3 0.144 0.019 7.466 0.000 0.436

WB6T2 WITH

WB6T3 0.179 0.017 10.378 0.000 0.364

WB7T1 WITH

WB7T2 0.177 0.023 7.670 0.000 0.353

WB7T3 0.090 0.026 3.479 0.001 0.467

WB7T2 WITH

WB7T3 0.147 0.022 6.697 0.000 0.428

Means

RI_PA 0.000 0.000 999.000 999.000 0.000

RI_SL 0.000 0.000 999.000 999.000 0.000

RI_WB 0.000 0.000 999.000 999.000 0.000

W_PAT1 0.000 0.000 999.000 999.000 0.000

W_PAT2 0.000 0.000 999.000 999.000 0.000

W_PAT3 0.000 0.000 999.000 999.000 0.000

W_SLT1 0.000 0.000 999.000 999.000 0.000

W_SLT2 0.000 0.000 999.000 999.000 0.000

W_SLT3 0.000 0.000 999.000 999.000 0.000

W_WBT1 0.000 0.000 999.000 999.000 0.000

W_WBT2 0.000 0.000 999.000 999.000 0.000

W_WBT3 0.000 0.000 999.000 999.000 0.000

Intercepts

PAT1 3.804 0.039 97.635 0.000 0.232

PAT2 3.764 0.039 95.684 0.000 0.320

PAT3 3.648 0.038 95.531 0.000 0.246

WBT1 0.000 0.000 999.000 999.000 0.000

WBT2 -0.037 0.023 -1.596 0.111 0.273

WBT3 -0.047 0.026 -1.828 0.067 0.155

Thresholds

WB1T1$1 -1.955 0.043 -45.609 0.000 0.202

WB1T1$2 -1.056 0.028 -37.156 0.000 0.156

WB1T1$3 -0.017 0.019 -0.906 0.365 0.131

WB1T1$4 1.108 0.028 39.844 0.000 0.252

WB2T1$1 -2.231 0.045 -49.049 0.000 0.214

WB2T1$2 -1.183 0.031 -37.585 0.000 0.168

WB2T1$3 0.034 0.023 1.467 0.142 0.125

WB2T1$4 1.425 0.030 46.906 0.000 0.227

WB3T1$1 -2.380 0.048 -49.934 0.000 0.242

WB3T1$2 -1.195 0.033 -36.745 0.000 0.204

WB3T1$3 0.000 0.025 -0.014 0.989 0.151

WB3T1$4 1.386 0.032 43.900 0.000 0.257

WB4T1$1 -2.166 0.047 -45.632 0.000 0.222

WB4T1$2 -1.179 0.033 -36.190 0.000 0.190

WB4T1$3 -0.039 0.024 -1.626 0.104 0.123

WB4T1$4 1.297 0.035 37.202 0.000 0.159

WB5T1$1 -2.794 0.054 -51.407 0.000 0.327

WB5T1$2 -1.537 0.040 -38.773 0.000 0.253

WB5T1$3 -0.155 0.031 -4.930 0.000 0.127

WB5T1$4 1.402 0.039 35.827 0.000 0.165

WB6T1$1 -2.295 0.043 -53.538 0.000 0.271

WB6T1$2 -1.493 0.033 -45.228 0.000 0.237

WB6T1$3 -0.518 0.023 -22.907 0.000 0.145

WB6T1$4 0.711 0.023 31.533 0.000 0.186

WB7T1$1 -2.708 0.050 -54.587 0.000 0.266

WB7T1$2 -1.763 0.035 -49.768 0.000 0.247

WB7T1$3 -0.637 0.026 -24.117 0.000 0.178

WB7T1$4 0.710 0.028 25.245 0.000 0.158

WB1T2$1 -1.955 0.043 -45.609 0.000 0.202

WB1T2$2 -1.056 0.028 -37.156 0.000 0.156

WB1T2$3 -0.017 0.019 -0.906 0.365 0.131

WB1T2$4 1.108 0.028 39.844 0.000 0.252

WB2T2$1 -2.231 0.045 -49.049 0.000 0.214

WB2T2$2 -1.183 0.031 -37.585 0.000 0.168

WB2T2$3 0.034 0.023 1.467 0.142 0.125

WB2T2$4 1.425 0.030 46.906 0.000 0.227

WB3T2$1 -2.380 0.048 -49.934 0.000 0.242

WB3T2$2 -1.195 0.033 -36.745 0.000 0.204

WB3T2$3 0.000 0.025 -0.014 0.989 0.151

WB3T2$4 1.386 0.032 43.900 0.000 0.257

WB4T2$1 -2.166 0.047 -45.632 0.000 0.222

WB4T2$2 -1.179 0.033 -36.190 0.000 0.190

WB4T2$3 -0.039 0.024 -1.626 0.104 0.123

WB4T2$4 1.297 0.035 37.202 0.000 0.159

WB5T2$1 -2.794 0.054 -51.407 0.000 0.327

WB5T2$2 -1.537 0.040 -38.773 0.000 0.253

WB5T2$3 -0.155 0.031 -4.930 0.000 0.127

WB5T2$4 1.402 0.039 35.827 0.000 0.165

WB6T2$1 -2.295 0.043 -53.538 0.000 0.271

WB6T2$2 -1.493 0.033 -45.228 0.000 0.237

WB6T2$3 -0.518 0.023 -22.907 0.000 0.145

WB6T2$4 0.711 0.023 31.533 0.000 0.186

WB7T2$1 -2.708 0.050 -54.587 0.000 0.266

WB7T2$2 -1.763 0.035 -49.768 0.000 0.247

WB7T2$3 -0.637 0.026 -24.117 0.000 0.178

WB7T2$4 0.710 0.028 25.245 0.000 0.158

WB1T3$1 -1.955 0.043 -45.609 0.000 0.202

WB1T3$2 -1.056 0.028 -37.156 0.000 0.156

WB1T3$3 -0.017 0.019 -0.906 0.365 0.131

WB1T3$4 1.108 0.028 39.844 0.000 0.252

WB2T3$1 -2.231 0.045 -49.049 0.000 0.214

WB2T3$2 -1.183 0.031 -37.585 0.000 0.168

WB2T3$3 0.034 0.023 1.467 0.142 0.125

WB2T3$4 1.425 0.030 46.906 0.000 0.227

WB3T3$1 -2.380 0.048 -49.934 0.000 0.242

WB3T3$2 -1.195 0.033 -36.745 0.000 0.204

WB3T3$3 0.000 0.025 -0.014 0.989 0.151

WB3T3$4 1.386 0.032 43.900 0.000 0.257

WB4T3$1 -2.166 0.047 -45.632 0.000 0.222

WB4T3$2 -1.179 0.033 -36.190 0.000 0.190

WB4T3$3 -0.039 0.024 -1.626 0.104 0.123

WB4T3$4 1.297 0.035 37.202 0.000 0.159

WB5T3$1 -2.794 0.054 -51.407 0.000 0.327

WB5T3$2 -1.537 0.040 -38.773 0.000 0.253

WB5T3$3 -0.155 0.031 -4.930 0.000 0.127

WB5T3$4 1.402 0.039 35.827 0.000 0.165

WB6T3$1 -2.295 0.043 -53.538 0.000 0.271

WB6T3$2 -1.493 0.033 -45.228 0.000 0.237

WB6T3$3 -0.518 0.023 -22.907 0.000 0.145

WB6T3$4 0.711 0.023 31.533 0.000 0.186

WB7T3$1 -2.708 0.050 -54.587 0.000 0.266

WB7T3$2 -1.763 0.035 -49.768 0.000 0.247

WB7T3$3 -0.637 0.026 -24.117 0.000 0.178

WB7T3$4 0.710 0.028 25.245 0.000 0.158

SLT1$1 -0.470 0.026 -17.904 0.000 0.229

SLT2$1 -0.303 0.027 -11.411 0.000 0.349

SLT3$1 -0.132 0.027 -4.931 0.000 0.227

Variances

RI_PA 2.008 0.103 19.576 0.000 0.284

RI_SL 0.175 0.120 1.467 0.143 0.482

RI_WB 0.372 0.030 12.529 0.000 0.392

W_PAT1 3.394 0.128 26.585 0.000 0.203

W_PAT2 3.182 0.120 26.432 0.000 0.146

W_PAT3 3.110 0.105 29.501 0.000 0.154

W_SLT1 1.000 0.000 999.000 999.000 0.000

W_SLT2 1.000 0.000 999.000 999.000 0.000

W_SLT3 1.000 0.000 999.000 999.000 0.000

W_WBT1 0.637 0.039 16.528 0.000 0.264

W_WBT2 0.725 0.039 18.414 0.000 0.251

W_WBT3 0.901 0.044 20.459 0.000 0.234

Residual Variances

WB1T1 1.000 0.000 999.000 999.000 0.000

WB2T1 1.000 0.000 999.000 999.000 0.000

WB3T1 1.000 0.000 999.000 999.000 0.000

WB4T1 1.000 0.000 999.000 999.000 0.000

WB5T1 1.000 0.000 999.000 999.000 0.000

WB6T1 1.000 0.000 999.000 999.000 0.000

WB7T1 1.000 0.000 999.000 999.000 0.000

WB1T2 0.753 0.034 21.884 0.000 0.338

WB2T2 0.749 0.034 22.173 0.000 0.263

WB3T2 0.912 0.037 24.655 0.000 0.354

WB4T2 0.958 0.045 21.261 0.000 0.197

WB5T2 0.885 0.042 20.974 0.000 0.379

WB6T2 0.869 0.038 22.677 0.000 0.337

WB7T2 0.900 0.041 21.746 0.000 0.294

WB1T3 0.661 0.031 21.155 0.000 0.355

WB2T3 0.606 0.029 21.171 0.000 0.353

WB3T3 0.864 0.040 21.473 0.000 0.365

WB4T3 0.771 0.034 22.748 0.000 0.294

WB5T3 0.820 0.040 20.272 0.000 0.388

WB6T3 0.679 0.029 23.757 0.000 0.354

WB7T3 0.847 0.040 21.103 0.000 0.339

SLT1 0.000 0.000 999.000 999.000 0.000

SLT2 0.000 0.000 999.000 999.000 0.000

SLT3 0.000 0.000 999.000 999.000 0.000

PAT1 0.000 0.000 999.000 999.000 0.000

PAT2 0.000 0.000 999.000 999.000 0.000

PAT3 0.000 0.000 999.000 999.000 0.000

WBT1 0.000 0.000 999.000 999.000 0.000

WBT2 0.000 0.000 999.000 999.000 0.000

WBT3 0.000 0.000 999.000 999.000 0.000

Group FEMALE

WBT1 BY

WB1T1 0.723 0.020 35.385 0.000 0.274

WB2T1 0.981 0.025 39.835 0.000 0.321

WB3T1 1.000 0.000 999.000 999.000 0.000

WB4T1 1.005 0.027 36.674 0.000 0.328

WB5T1 1.341 0.034 39.466 0.000 0.359

WB6T1 0.795 0.021 38.646 0.000 0.338

WB7T1 1.019 0.027 38.110 0.000 0.347

WBT2 BY

WB1T2 0.723 0.020 35.385 0.000 0.274

WB2T2 0.981 0.025 39.835 0.000 0.321

WB3T2 1.000 0.000 999.000 999.000 0.000

WB4T2 1.005 0.027 36.674 0.000 0.328

WB5T2 1.341 0.034 39.466 0.000 0.359

WB6T2 0.795 0.021 38.646 0.000 0.338

WB7T2 1.019 0.027 38.110 0.000 0.347

WBT3 BY

WB1T3 0.723 0.020 35.385 0.000 0.274

WB2T3 0.981 0.025 39.835 0.000 0.321

WB3T3 1.000 0.000 999.000 999.000 0.000

WB4T3 1.005 0.027 36.674 0.000 0.328

WB5T3 1.341 0.034 39.466 0.000 0.359

WB6T3 0.795 0.021 38.646 0.000 0.338

WB7T3 1.019 0.027 38.110 0.000 0.347

RI_PA BY

PAT1 1.000 0.000 999.000 999.000 0.000

PAT2 1.000 0.000 999.000 999.000 0.000

PAT3 1.000 0.000 999.000 999.000 0.000

RI_SL BY

SLT1 1.000 0.000 999.000 999.000 0.000

SLT2 1.000 0.000 999.000 999.000 0.000

SLT3 1.000 0.000 999.000 999.000 0.000

W_PAT1 BY

PAT1 1.000 0.000 999.000 999.000 0.000

W_PAT2 BY

PAT2 1.000 0.000 999.000 999.000 0.000

W_PAT3 BY

PAT3 1.000 0.000 999.000 999.000 0.000

W_SLT1 BY

SLT1 1.000 0.000 999.000 999.000 0.000

W_SLT2 BY

SLT2 1.000 0.000 999.000 999.000 0.000

W_SLT3 BY

SLT3 1.000 0.000 999.000 999.000 0.000

RI_WB BY

WBT1 1.000 0.000 999.000 999.000 0.000

WBT2 1.000 0.000 999.000 999.000 0.000

WBT3 1.000 0.000 999.000 999.000 0.000

W_WBT1 BY

WBT1 1.000 0.000 999.000 999.000 0.000

W_WBT2 BY

WBT2 1.000 0.000 999.000 999.000 0.000

W_WBT3 BY

WBT3 1.000 0.000 999.000 999.000 0.000

W_PAT2 ON

W_PAT1 0.137 0.029 4.762 0.000 0.348

W_SLT1 0.013 0.063 0.212 0.832 0.348

W_WBT1 0.091 0.066 1.380 0.168 0.425

W_SLT2 ON

W_PAT1 -0.021 0.021 -1.028 0.304 0.441

W_SLT1 0.140 0.057 2.437 0.015 0.376

W_WBT1 0.169 0.055 3.102 0.002 0.375

W_WBT2 ON

W_PAT1 -0.004 0.013 -0.307 0.759 0.405

W_SLT1 0.069 0.033 2.058 0.040 0.432

W_WBT1 0.189 0.040 4.765 0.000 0.496

W_PAT3 ON

W_PAT2 0.167 0.033 5.031 0.000 0.452

W_SLT2 0.026 0.058 0.448 0.654 0.362

W_WBT2 -0.019 0.065 -0.290 0.772 0.417

W_SLT3 ON

W_PAT2 -0.027 0.021 -1.259 0.208 0.344

W_SLT2 0.196 0.051 3.837 0.000 0.371

W_WBT2 0.050 0.046 1.081 0.280 0.436

W_WBT3 ON

W_PAT2 -0.009 0.013 -0.723 0.470 0.363

W_SLT2 0.038 0.028 1.368 0.171 0.346

W_WBT2 0.230 0.033 6.972 0.000 0.446

WBT1 ON

ETH 0.183 0.017 10.713 0.000 0.111

FSM -0.136 0.017 -8.028 0.000 0.140

SEN -0.169 0.022 -7.856 0.000 0.132

WBT2 ON

ETH 0.183 0.017 10.713 0.000 0.111

FSM -0.136 0.017 -8.028 0.000 0.140

SEN -0.169 0.022 -7.856 0.000 0.132

WBT3 ON

ETH 0.183 0.017 10.713 0.000 0.111

FSM -0.136 0.017 -8.028 0.000 0.140

SEN -0.169 0.022 -7.856 0.000 0.132

PAT1 ON

ETH -0.426 0.039 -11.061 0.000 0.171

FSM -0.210 0.037 -5.642 0.000 0.140

SEN -0.331 0.044 -7.552 0.000 0.103

PAT2 ON

ETH -0.426 0.039 -11.061 0.000 0.171

FSM -0.210 0.037 -5.642 0.000 0.140

SEN -0.331 0.044 -7.552 0.000 0.103

PAT3 ON

ETH -0.426 0.039 -11.061 0.000 0.171

FSM -0.210 0.037 -5.642 0.000 0.140

SEN -0.331 0.044 -7.552 0.000 0.103

SLT1 ON

ETH 0.219 0.024 9.137 0.000 0.126

FSM -0.163 0.023 -7.030 0.000 0.149

SEN -0.072 0.028 -2.551 0.011 0.141

SLT2 ON

ETH 0.219 0.024 9.137 0.000 0.126

FSM -0.163 0.023 -7.030 0.000 0.149

SEN -0.072 0.028 -2.551 0.011 0.141

SLT3 ON

ETH 0.219 0.024 9.137 0.000 0.126

FSM -0.163 0.023 -7.030 0.000 0.149

SEN -0.072 0.028 -2.551 0.011 0.141

RI_PA WITH

RI_SL 0.194 0.049 3.926 0.000 0.294

RI_WB 0.201 0.033 6.067 0.000 0.362

RI_SL WITH

RI_WB 0.377 0.034 11.237 0.000 0.394

W_PAT1 WITH

W_WBT1 0.184 0.036 5.159 0.000 0.387

W_SLT1 0.172 0.054 3.173 0.002 0.322

W_WBT1 WITH

W_SLT1 0.372 0.028 13.201 0.000 0.356

W_PAT2 WITH

W_SLT2 0.026 0.049 0.532 0.595 0.330

W_WBT2 0.089 0.033 2.705 0.007 0.337

W_SLT2 WITH

W_WBT2 0.287 0.024 11.793 0.000 0.299

W_PAT3 WITH

W_SLT3 0.104 0.039 2.655 0.008 0.289

W_WBT3 0.099 0.029 3.453 0.001 0.288

W_SLT3 WITH

W_WBT3 0.246 0.022 11.388 0.000 0.380

WB1T1 WITH

WB1T2 0.212 0.018 11.783 0.000 0.417

WB1T3 0.144 0.016 8.808 0.000 0.364

WB1T2 WITH

WB1T3 0.204 0.015 13.397 0.000 0.308

WB2T1 WITH

WB2T2 0.127 0.018 7.054 0.000 0.471

WB2T3 0.104 0.019 5.402 0.000 0.477

WB2T2 WITH

WB2T3 0.108 0.017 6.381 0.000 0.495

WB3T1 WITH

WB3T2 0.182 0.020 9.308 0.000 0.450

WB3T3 0.128 0.019 6.643 0.000 0.442

WB3T2 WITH

WB3T3 0.176 0.019 9.298 0.000 0.443

WB4T1 WITH

WB4T2 0.125 0.019 6.584 0.000 0.367

WB4T3 0.056 0.020 2.831 0.005 0.466

WB4T2 WITH

WB4T3 0.079 0.019 4.251 0.000 0.490

WB5T1 WITH

WB5T2 0.133 0.022 6.161 0.000 0.435

WB5T3 0.123 0.025 4.898 0.000 0.482

WB5T2 WITH

WB5T3 0.133 0.023 5.738 0.000 0.504

WB6T1 WITH

WB6T2 0.180 0.019 9.274 0.000 0.355

WB6T3 0.133 0.018 7.362 0.000 0.458

WB6T2 WITH

WB6T3 0.178 0.018 10.122 0.000 0.364

WB7T1 WITH

WB7T2 0.198 0.022 9.041 0.000 0.344

WB7T3 0.129 0.023 5.739 0.000 0.334

WB7T2 WITH

WB7T3 0.189 0.022 8.490 0.000 0.463

Means

RI_PA 0.000 0.000 999.000 999.000 0.000

RI_SL 0.000 0.000 999.000 999.000 0.000

RI_WB 0.000 0.000 999.000 999.000 0.000

W_PAT1 0.000 0.000 999.000 999.000 0.000

W_PAT2 0.000 0.000 999.000 999.000 0.000

W_PAT3 0.000 0.000 999.000 999.000 0.000

W_SLT1 0.000 0.000 999.000 999.000 0.000

W_SLT2 0.000 0.000 999.000 999.000 0.000

W_SLT3 0.000 0.000 999.000 999.000 0.000

W_WBT1 0.000 0.000 999.000 999.000 0.000

W_WBT2 0.000 0.000 999.000 999.000 0.000

W_WBT3 0.000 0.000 999.000 999.000 0.000

Intercepts

PAT1 3.804 0.039 97.635 0.000 0.232

PAT2 3.764 0.039 95.684 0.000 0.320

PAT3 3.648 0.038 95.531 0.000 0.246

WBT1 -0.440 0.033 -13.391 0.000 0.104

WBT2 -0.554 0.036 -15.561 0.000 0.110

WBT3 -0.517 0.034 -15.043 0.000 0.160

Thresholds

WB1T1$1 -1.955 0.043 -45.609 0.000 0.202

WB1T1$2 -1.056 0.028 -37.156 0.000 0.156

WB1T1$3 -0.017 0.019 -0.906 0.365 0.131

WB1T1$4 1.108 0.028 39.844 0.000 0.252

WB2T1$1 -2.231 0.045 -49.049 0.000 0.214

WB2T1$2 -1.183 0.031 -37.585 0.000 0.168

WB2T1$3 0.034 0.023 1.467 0.142 0.125

WB2T1$4 1.425 0.030 46.906 0.000 0.227

WB3T1$1 -2.380 0.048 -49.934 0.000 0.242

WB3T1$2 -1.195 0.033 -36.745 0.000 0.204

WB3T1$3 0.000 0.025 -0.014 0.989 0.151

WB3T1$4 1.386 0.032 43.900 0.000 0.257

WB4T1$1 -2.166 0.047 -45.632 0.000 0.222

WB4T1$2 -1.179 0.033 -36.190 0.000 0.190

WB4T1$3 -0.039 0.024 -1.626 0.104 0.123

WB4T1$4 1.297 0.035 37.202 0.000 0.159

WB5T1$1 -2.794 0.054 -51.407 0.000 0.327

WB5T1$2 -1.537 0.040 -38.773 0.000 0.253

WB5T1$3 -0.155 0.031 -4.930 0.000 0.127

WB5T1$4 1.402 0.039 35.827 0.000 0.165

WB6T1$1 -2.295 0.043 -53.538 0.000 0.271

WB6T1$2 -1.493 0.033 -45.228 0.000 0.237

WB6T1$3 -0.518 0.023 -22.907 0.000 0.145

WB6T1$4 0.711 0.023 31.533 0.000 0.186

WB7T1$1 -2.708 0.050 -54.587 0.000 0.266

WB7T1$2 -1.763 0.035 -49.768 0.000 0.247

WB7T1$3 -0.637 0.026 -24.117 0.000 0.178

WB7T1$4 0.710 0.028 25.245 0.000 0.158

WB1T2$1 -1.955 0.043 -45.609 0.000 0.202

WB1T2$2 -1.056 0.028 -37.156 0.000 0.156

WB1T2$3 -0.017 0.019 -0.906 0.365 0.131

WB1T2$4 1.108 0.028 39.844 0.000 0.252

WB2T2$1 -2.231 0.045 -49.049 0.000 0.214

WB2T2$2 -1.183 0.031 -37.585 0.000 0.168

WB2T2$3 0.034 0.023 1.467 0.142 0.125

WB2T2$4 1.425 0.030 46.906 0.000 0.227

WB3T2$1 -2.380 0.048 -49.934 0.000 0.242

WB3T2$2 -1.195 0.033 -36.745 0.000 0.204

WB3T2$3 0.000 0.025 -0.014 0.989 0.151

WB3T2$4 1.386 0.032 43.900 0.000 0.257

WB4T2$1 -2.166 0.047 -45.632 0.000 0.222

WB4T2$2 -1.179 0.033 -36.190 0.000 0.190

WB4T2$3 -0.039 0.024 -1.626 0.104 0.123

WB4T2$4 1.297 0.035 37.202 0.000 0.159

WB5T2$1 -2.794 0.054 -51.407 0.000 0.327

WB5T2$2 -1.537 0.040 -38.773 0.000 0.253

WB5T2$3 -0.155 0.031 -4.930 0.000 0.127

WB5T2$4 1.402 0.039 35.827 0.000 0.165

WB6T2$1 -2.295 0.043 -53.538 0.000 0.271

WB6T2$2 -1.493 0.033 -45.228 0.000 0.237

WB6T2$3 -0.518 0.023 -22.907 0.000 0.145

WB6T2$4 0.711 0.023 31.533 0.000 0.186

WB7T2$1 -2.708 0.050 -54.587 0.000 0.266

WB7T2$2 -1.763 0.035 -49.768 0.000 0.247

WB7T2$3 -0.637 0.026 -24.117 0.000 0.178

WB7T2$4 0.710 0.028 25.245 0.000 0.158

WB1T3$1 -1.955 0.043 -45.609 0.000 0.202

WB1T3$2 -1.056 0.028 -37.156 0.000 0.156

WB1T3$3 -0.017 0.019 -0.906 0.365 0.131

WB1T3$4 1.108 0.028 39.844 0.000 0.252

WB2T3$1 -2.231 0.045 -49.049 0.000 0.214

WB2T3$2 -1.183 0.031 -37.585 0.000 0.168

WB2T3$3 0.034 0.023 1.467 0.142 0.125

WB2T3$4 1.425 0.030 46.906 0.000 0.227

WB3T3$1 -2.380 0.048 -49.934 0.000 0.242

WB3T3$2 -1.195 0.033 -36.745 0.000 0.204

WB3T3$3 0.000 0.025 -0.014 0.989 0.151

WB3T3$4 1.386 0.032 43.900 0.000 0.257

WB4T3$1 -2.166 0.047 -45.632 0.000 0.222

WB4T3$2 -1.179 0.033 -36.190 0.000 0.190

WB4T3$3 -0.039 0.024 -1.626 0.104 0.123

WB4T3$4 1.297 0.035 37.202 0.000 0.159

WB5T3$1 -2.794 0.054 -51.407 0.000 0.327

WB5T3$2 -1.537 0.040 -38.773 0.000 0.253

WB5T3$3 -0.155 0.031 -4.930 0.000 0.127

WB5T3$4 1.402 0.039 35.827 0.000 0.165

WB6T3$1 -2.295 0.043 -53.538 0.000 0.271

WB6T3$2 -1.493 0.033 -45.228 0.000 0.237

WB6T3$3 -0.518 0.023 -22.907 0.000 0.145

WB6T3$4 0.711 0.023 31.533 0.000 0.186

WB7T3$1 -2.708 0.050 -54.587 0.000 0.266

WB7T3$2 -1.763 0.035 -49.768 0.000 0.247

WB7T3$3 -0.637 0.026 -24.117 0.000 0.178

WB7T3$4 0.710 0.028 25.245 0.000 0.158

SLT1$1 -0.470 0.026 -17.904 0.000 0.229

SLT2$1 -0.303 0.027 -11.411 0.000 0.349

SLT3$1 -0.132 0.027 -4.931 0.000 0.227

Variances

RI_PA 1.801 0.090 20.069 0.000 0.304

RI_SL 0.842 0.081 10.370 0.000 0.385

RI_WB 0.365 0.028 12.851 0.000 0.346

W_PAT1 2.879 0.103 27.892 0.000 0.213

W_PAT2 2.510 0.111 22.566 0.000 0.138

W_PAT3 2.569 0.086 29.860 0.000 0.202

W_SLT1 1.000 0.000 999.000 999.000 0.000

W_SLT2 1.000 0.000 999.000 999.000 0.000

W_SLT3 1.000 0.000 999.000 999.000 0.000

W_WBT1 0.637 0.037 17.382 0.000 0.308

W_WBT2 0.613 0.033 18.355 0.000 0.305

W_WBT3 0.669 0.033 20.122 0.000 0.301

Residual Variances

WB1T1 0.943 0.050 18.881 0.000 0.237

WB2T1 0.974 0.047 20.659 0.000 0.202

WB3T1 0.872 0.039 22.359 0.000 0.264

WB4T1 0.927 0.048 19.390 0.000 0.175

WB5T1 0.760 0.035 21.891 0.000 0.339

WB6T1 0.964 0.045 21.648 0.000 0.275

WB7T1 1.010 0.045 22.262 0.000 0.222

WB1T2 0.719 0.038 18.711 0.000 0.286

WB2T2 0.765 0.038 20.353 0.000 0.319

WB3T2 0.793 0.038 21.146 0.000 0.322

WB4T2 0.862 0.043 19.849 0.000 0.257

WB5T2 0.664 0.035 19.075 0.000 0.419

WB6T2 0.859 0.041 20.790 0.000 0.338

WB7T2 0.970 0.045 21.617 0.000 0.258

WB1T3 0.590 0.031 19.026 0.000 0.308

WB2T3 0.619 0.030 20.396 0.000 0.368

WB3T3 0.765 0.035 21.557 0.000 0.349

WB4T3 0.676 0.035 19.154 0.000 0.299

WB5T3 0.650 0.031 21.000 0.000 0.379

WB6T3 0.760 0.035 21.686 0.000 0.349

WB7T3 0.903 0.042 21.410 0.000 0.298

SLT1 0.000 0.000 999.000 999.000 0.000

SLT2 0.000 0.000 999.000 999.000 0.000

SLT3 0.000 0.000 999.000 999.000 0.000

PAT1 0.000 0.000 999.000 999.000 0.000

PAT2 0.000 0.000 999.000 999.000 0.000

PAT3 0.000 0.000 999.000 999.000 0.000

WBT1 0.000 0.000 999.000 999.000 0.000

WBT2 0.000 0.000 999.000 999.000 0.000

WBT3 0.000 0.000 999.000 999.000 0.000
